# Maximum likelihood estimators are ineffective for acoustic detection of rare bat species

**DOI:** 10.1371/journal.pone.0320646

**Published:** 2025-04-01

**Authors:** Bradley H. Hopp, Donald I. Solick, John Chenger, Christian M. Newman

**Affiliations:** 1 Electric Power Research Institute, Palo Alto, California, United States of America; 2 Vesper Bat Echolocation Specialists, Fort Collins, Colorado, United States of America,; 3 Bat Conservation and Management, Carlisle, Pennsylvania, United States of America; Bowling Green State University, UNITED STATES OF AMERICA

## Abstract

Acoustic monitoring is an important tool for determining presence or probable absence of threatened and endangered bats in the United States (US). Federal guidance requires the use of automated identification programs that classify audio files and calculate a Maximum Likelihood Estimator (MLE) for each bat species during each night of a survey. Acoustic presence or absence of species is based on a significant or non-significant MLE, which can have profound regulatory effects, positive or negative. Despite relying on this metric to determine presence of rare species for the past ten years, little is known about the number of files required by available programs to trigger significant MLE or the effect of species ratio on this calculation. We used 1,120 audio files containing echolocation calls from nine northeastern US bat species to simulate survey nights containing variable absolute counts and ratios of species’ audio files. We developed models to estimate the number of audio files that Kaleidoscope Pro (KPro) and SonoBat programs required to establish acoustic presence for each species, and we then applied our best model to a long-term acoustic dataset collected at the Fort Drum Military Installation in New York. Each program required a similar number of files to detect presence for some species, such as *Myotis septentrionalis* and *M. sodalis* (8 to 10 files), but differed in file requirements for other species, such as *Lasiurus cinereus* (KPro =  4; SonoBat =  7) and *Perimyotis subflavus* (KPro =  10; SonoBat =  6). Both programs performed poorly with determining presence for any species at low species ratio (<25%). Applying our model to the Fort Drum dataset revealed that the total number of audio files recorded within a night had a great effect on whether a rare species was correctly determined to be present. We conclude that MLE should be used with caution during surveys of rare species and could produce misleading results in certain conditions.

## Introduction

In the United States (US), several once-common species of bats are in severe decline. Range-wide summer occupancy has markedly decreased for northern long-eared bats (*Myotis septentrionalis*), little brown bats (*M. lucifugus*), and tricolored bats (*Perimyotis subflavus*; [1]) due to White Nose Syndrome (WNS), a disease caused by an invasive, pathogenic fungus (*Pseudogymnoascus destructans*) that impacts hibernating species [2]. The U.S. Fish and Wildlife Service (USFWS) recently upgraded the status of *M. septentrionalis* from threatened to endangered [3], proposed that *P. subflavus* be listed as endangered [4], and placed *M. lucifugus* on the National Domestic Listing Workplan for review in 2026 [5]. In addition to WNS, bats face other stressors. For example, hoary bats (*Lasiurus cinereus*) may be in decline due to collisions with wind turbine blades [6,7], and are on the National Domestic Listing Workplan for review in 2028 [7]. As these bats become less abundant on the landscape, traditional capture and hibernacula survey methods may become less adequate for tracking population trends or determining regulatory compliance.

Acoustic monitoring has become an important tool for surveys of rare species in general [8–10] and is already an important component for surveys of *M. septentrionalis* and Indiana bats (*M. sodalis*), another endangered US species [11]. During these surveys, ultrasonic detectors are deployed for a prescribed number of nights to record bat echolocation calls. Automated classification software programs, such as Kaleidoscope Pro (KPro; Wildlife Acoustics, Massachusetts) and SonoBat (Arcata, California), are used to classify recorded audio files to species and calculate nightly maximum likelihood estimator (MLE) values for each species detected [12]. This metric compares the absolute count of files classified as a species with the known cross-species misclassification error rate for other species that were identified during a night of recordings [13]. An MLE *P-*value ≤  0.05 indicates that the number of classified audio files exceeds the known misclassification rates for that species in the program’s classifier algorithm [14], and establishes statistical, acoustic presence for that night of the survey.

The MLE calculations are program-specific because manufacturers use different sets of echolocation calls to create cross-classification rates. Also, *P-*values can be affected by the number of audio files classified as a particular species, the ratio of bat species recorded on a particular night, and the accuracy of those classifications. Solick et al. [15] recently found that the file identification accuracy of KPro and SonoBat varies for northeastern US species, with lower accuracy for acoustically ambiguous species, such as *M. septentrionalis* and *M. sodalis* that can have a high degree of echolocation characteristic overlap with other species, and higher accuracy for species that produce more distinct calls, such as *L. cinereus* and *P. subflavus*. For example, with file identification sensitivity ranging between 0.38 and 0.52 for *M. septentrionalis* [15] acoustic surveys would likely need to record many high-quality bat calls by this species on a single night to be considered acoustically present (MLE *P* ≤  0.05), compared to *P. subflavus* (sensitivity 0.91-0.97; [15]). Recording enough echolocation calls to trigger a determination of presence by automated classifiers could be a challenge for rare species [16,17]. However, low sensitivity may not be an issue if a program can compensate for misclassification rates when calculating MLE.

Understanding the minimum number of audio files needed to yield accurate MLE-based acoustic presence for a species by software under a range of different species ratios would be valuable for making informed bat conservation and management decisions. We simulated survey nights containing variable counts and ratios of audio files for nine northeastern US bat species to develop models that estimated the number of audio files each program requires to establish acoustic presence for each species. We then applied our best model to a long-term acoustic dataset collected at the Fort Drum Military Installation in New York, USA, to evaluate the implications of using MLE to determine species presence in a real-world scenario.

## Methods

We used 1,120 full-spectrum audio files that contained known echolocation calls for nine bat species that occur in the northeastern US: big brown bat (*Eptesicus fuscus*), eastern red bat (*Lasiurus borealis*), hoary bat, silver-haired bat (*Lasionycteris noctivagans*), eastern small-footed bat (*Myotis leibii*), little brown bat, northern long-eared bat, Indiana bat, and tricolored bat. This species assemblage included endangered US species of interest, and consisted of the nine species found in New York, as well as the greater New England region including also New Hampshire, Vermont, Connecticut, Rhode Island, and Massachusetts. Collection methods for these audio files are described in Solick et al. 2024 [15]. All files were high-quality recordings of single, free-flying bats positively identified prior to release, and contained a minimum of five search phase pulses [18].

### Software parameters

We used the most recent version of SonoBat (30.1) [19], and version 5.4.7 for KPro, which is the most recent USFWS-approved version for stand-alone acoustic surveys of endangered species in the eastern U.S. [20]. SonoBat is currently a candidate for approval and must be used in conjunction with KPro for acoustic surveys of endangered species in the eastern U.S [11]. For SonoBat, we used the nE[c20240921] region pack and the NY-PA subregion pack within the SonoBat classifier for northeast North America. We used default settings for Sequence Decision Threshold (0.90), Acceptable Call Quality (0.6), and Maximum Number of Calls to Consider per File (32). For KPro, we used the New York set of species, limited to the nine species in this study, and the Bats of North America 5.4.0 classifier. We used default settings with the following modifications: we selected Advanced Signal Processing, Minimum and Maximum Frequency Range (8–120 kHz), Minimum and Maximum Length of Detected Pulses (2-500 ms), and Maximum Inter-syllable Gap (500 ms). We also selected 5 as the Minimum Number of Pulses and “-1 (More Sensitive)” as the Sensitivity setting, as per USFWS guidance [21].

### Simulated nights

**Fig 1:**
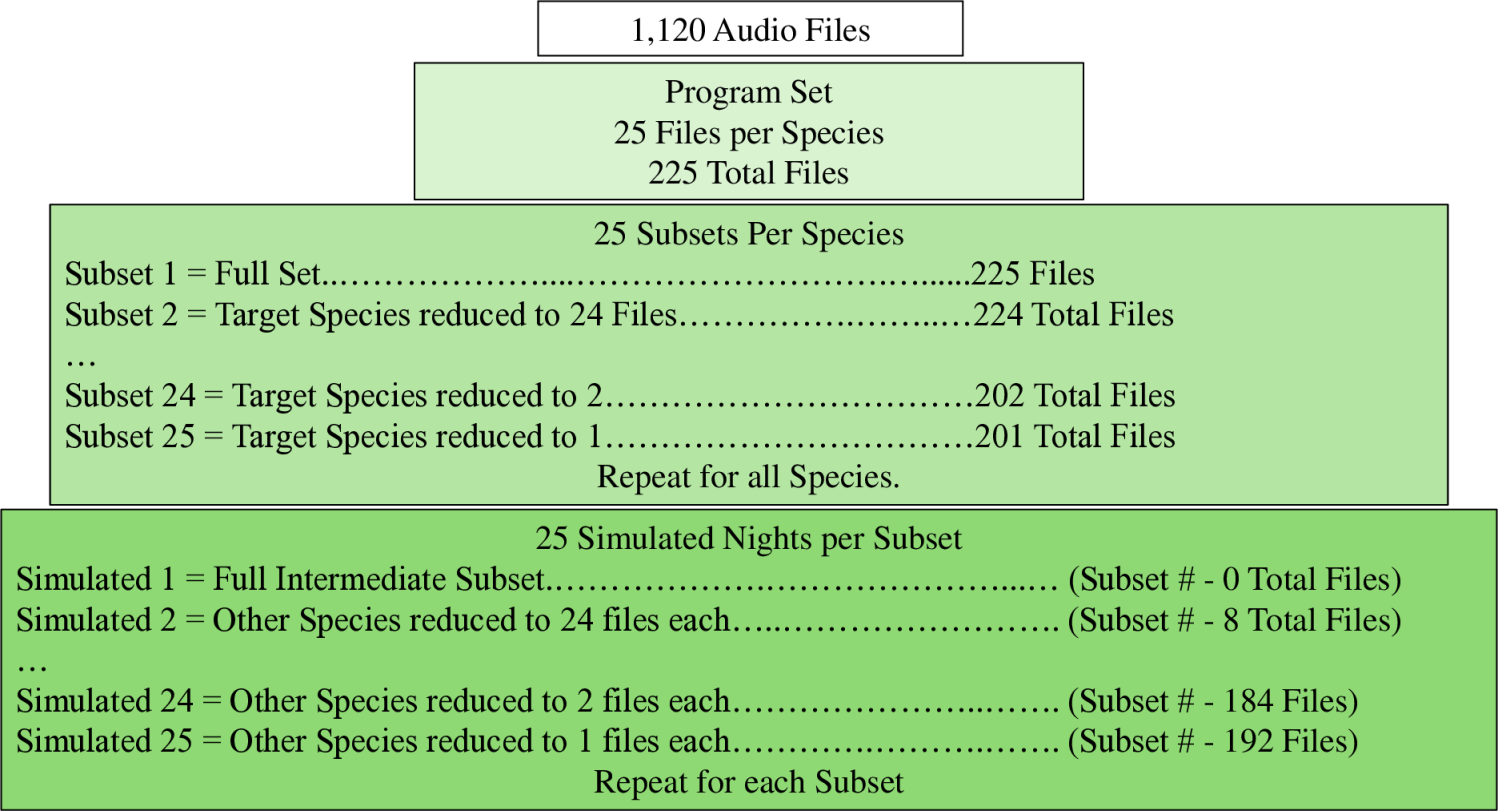
Diagram of simulated night generation. 1,120 acoustic files with known species identity were used to generate simulated nights for Kaleidoscope Pro and SonoBat. Subsets were used to assist in the creation of simulated nights but were not tested.

We designed nightly simulations for KPro and SonoBat that incorporated all nine northeastern US bat species at a range of species ratios. We define ‘species ratio’ as the proportion of echolocation calls recorded for a particular species within nights or across seasons and can either be manufactured for controlled simulations (this study) or based on a robust set of identified echolocation calls for a site or region. The study design for our simulations was based on the current USFWS protocol for testing of automated classifier software to detect acoustic presence of *M. septentrionalis* and *M. sodalis* [21]. To ensure we were evaluating the MLE algorithm of a program and not its identification algorithm, our simulations only used audio files that had previously been correctly classified to species by KPro and SonoBat automated classification programs [15]. However, due to a new release of the northeast US Region Pack (nE[c20240921]) by SonoBat we reran accuracy metrics using the methods outlined in Solick et al. 2024 ([15]; new SonoBat metrics found in S1 Table and S2 Table). This led to some species (*M. leibii* and *M. sodalis*) with duplicate audio files within a subset due to an insufficient number of correctly identified audio files (S3 Table). Preliminary analyses did not indicate an effect of duplicate files on MLE values for these species. For each program we created a set of 225 files with 25 randomly selected audio files for each species (Fig 1). Ford et al. [16] determined that 25 audio files consistently yielded correct MLE values for *M. septentrionalis* and *M. sodalis* using Kaleidoscope Pro (version 5.1.0), so we selected 25 audio files for each of the nine species.

For each program set we created 25 subsets per species, for a total of 225 subsets per program (Fig 1). The first subset for a species (hereby called ‘Target Species’) contained all 225 audio files from the set. For subsequent subsets, the number of audio files for the Target Species was reduced by one whereas the number of audio files for all other species (hereby called ‘Other Species’) remained 25. Subset 25 contained 1 audio file for the Target Species and 25 audio files for each Other Species. This process was repeated for each species until every species had been the Target Species once.

To better examine the effect of Other Species ratio on MLE we created 25 ‘simulated nights’ from each subset. Simulated nights had the same number of Target Species audio files as the subset they were derived from, but the number of audio files for each of the Other Species was reduced by one on each subsequent simulated night. A total of 5,625 simulated nights (9 species *  25 subsets per species *  25 simulated nights per subset) were used for each program during the study. This created sets whereby the mean relative abundance for all nights was 0.11 with a standard deviation of 0.05 (S1 Fig). Simulated nights were created using custom R scripts [22] (S1 Script and S2 Script). Each simulated night had nine MLE values, one for the Target Species and one for each of the Other Species, for a grand total of 50,625 MLE values. Hereafter, we use the term ‘Examined Species’ to refer to the species that MLE values pertain to in our analysis.

### Simulated nights dataset

Each simulated night processed by KPro and by SonoBat generated MLE values for each Examined Species. We considered MLE values with *P* ≤  0.05 to be a true positive (TP) indication of acoustic presence for that Examined Species because we knew at least one audio file was present for each species. We considered MLE values with *P* >  0.05 to be a false negative (FN) indication of acoustic presence for an Examined Species. TP/FN indications were then grouped by species and by the number of Examined Species audio files present in the simulated night for visualization in R [22].

To compare performance of the MLE algorithm between programs, we created the metric critical count (CC), or the minimum number of correctly classified audio files required for an automated classifier to accurately indicate statistical, acoustic presence of a species (MLE *P* ≤  0.05) at a given species ratio on a simulated night, knowing that it was actually present. We created another metric, real-world recordings (RWR), to estimate the number of field-recorded audio files each program would need for MLE *P* ≤  0.05. The RWR is the quotient of CC divided by the sensitivity for the species (provided in [15] for KPro and S1 Table for SonoBat). The RWR assumes that no other species were misclassified as the species of interest and that all species were either correctly classified or given no classification. For example, if many *M. lucifugus* and *M. sodalis* were recorded, a portion of each species would be expected to be misclassified as the other. For simplicity, RWR assumes this does not happen and therefore may overestimate the number of audio files needed since misclassified files could errantly contribute to a correct detection. In summary, CC informs how many correctly identified files are needed for a species to be declared present while RWR estimates how many files may need to be collected in the field to account for incorrect identification rates.

### Modeling

We trained general linear models on our simulated nights dataset to estimate program MLE values from species counts and species ratios not already explicitly tested in our simulated nights dataset. The simulated nights dataset for each program was run through custom R scripts (S3 Script–S5 Script). The script generated a model for each of the nine species in the study to estimate program MLE from the modeled species audio file count and species ratio (full output can be found in S1 File and S2 File). We used the R function ‘*glm*’ in the *‘stats*’ package [22] using family ‘*gaussian*’ and evaluated using ‘leave one out’ cross-validation. The different variable sets used are listed in S4 Table and coefficient, p-value, R^2^, root mean square error (RMSE) and mean absolute error (MAE) values can be found in S1 File and S2 File. All variables tested were derived from species count and species ratio and were tested to determine if different functions (i.e., logarithmic, exponential) better described the dataset. When visualizing the models the species ratio for all non-Examined Species were set equal. For example, if the Examined Species ratio was 0.20 then the eight non-Examined Species each had a species ratio of 0.10.

### Fort drum military installation case study

To understand how KPro and SonoBat might perform for determining acoustic presence at a site with known species counts and species ratio, we applied our model to acoustic survey data collected at the Fort Drum Military Installation (Fort Drum) in northwestern New York, U.S. [23,24]. Relative activity levels (bat passes per detector-night) for each of the nine species at Fort Drum were calculated during pre-WNS (summers, 2004-2007) and post-WNS (summers, 2008-2017; [24]). The two time periods allowed us to determine how population declines at a site might affect MLE values. The average number of bat audio files recorded on AnaBat detectors (including II, SD1, and SD2 models, Titley Electronics, Australia) across all species at Fort Drum was approximately 174 per night during pre-WNS and 73 during post-WNS (M. Ford, pers comm). For simplicity, we used 75 and 175 total bat audio files per night to represent the observed range of bat files recorded at Fort Drum. We also included arbitrary counts of 275 and 475 total bat audio files per night to show the effect of larger sample sizes on MLE presence. These larger numbers may also be more representative of nightly bat files recorded on full-spectrum detectors that typically detect more bats than zero-crossing AnaBat detectors [25]. We calculated the species ratio of each species during the pre- and post-WNS periods by dividing the mean activity level of each species reported in [23] by the sum of mean activity levels for all species by time period. We then determined the number of hypothetical audio files (count) for each species by multiplying the species ratio and the total number of representative audio files. We allowed for fractional counts, such as 0.1 bat calls in some cases. Though biologically unrealistic, our model does not distinguish between whole and fractional numbers, and this convention allowed us to estimate species ratios more precisely.

We applied known sensitivities and specificities for KPro and SonoBat ([15] and this study) to calculate the total positives (audio file true positives +  audio file false positives) for each species and program during pre- and post-WNS periods. For KPro, we used sensitivities and specificities reported in [15]. For SonoBat, the version of the Region Pack classifier used in Solick et al. 2024 ([15]; nE[c20231222]) has since been updated to version nE[c20240921], so we calculated the sensitivity and misclassification rates for nE[c20240921] (S1 Table and S2 Table). New species ratios were found based on each species’ total positive audio files, $\text{total positives derived species ratio }=\frac{\left(\text{total positive audio files for Examined Species}\right)}{\left(\text{total positive audio files for all nine species}\right)}$ . We input these total positive audio files and total positives derived species ratios into the nine species models to estimate MLE for KPro and SonoBat during each period. If the total positive audio files for a species was ≥  25 then the MLE was set to 0.00 regardless of the model’s prediction. This was due to our simulated nights dataset showing both programs yielded MLE *P* ≤  0.05 once or before the file count reached 25 for each Examined Species. We visualized resultant MLEs in R with the *ggplot2* package along with the ±  2 MAEs for each model. The R code can be found in S3 Script–S5 Script.

## Results

### Simulated nights dataset

**Fig 2:**
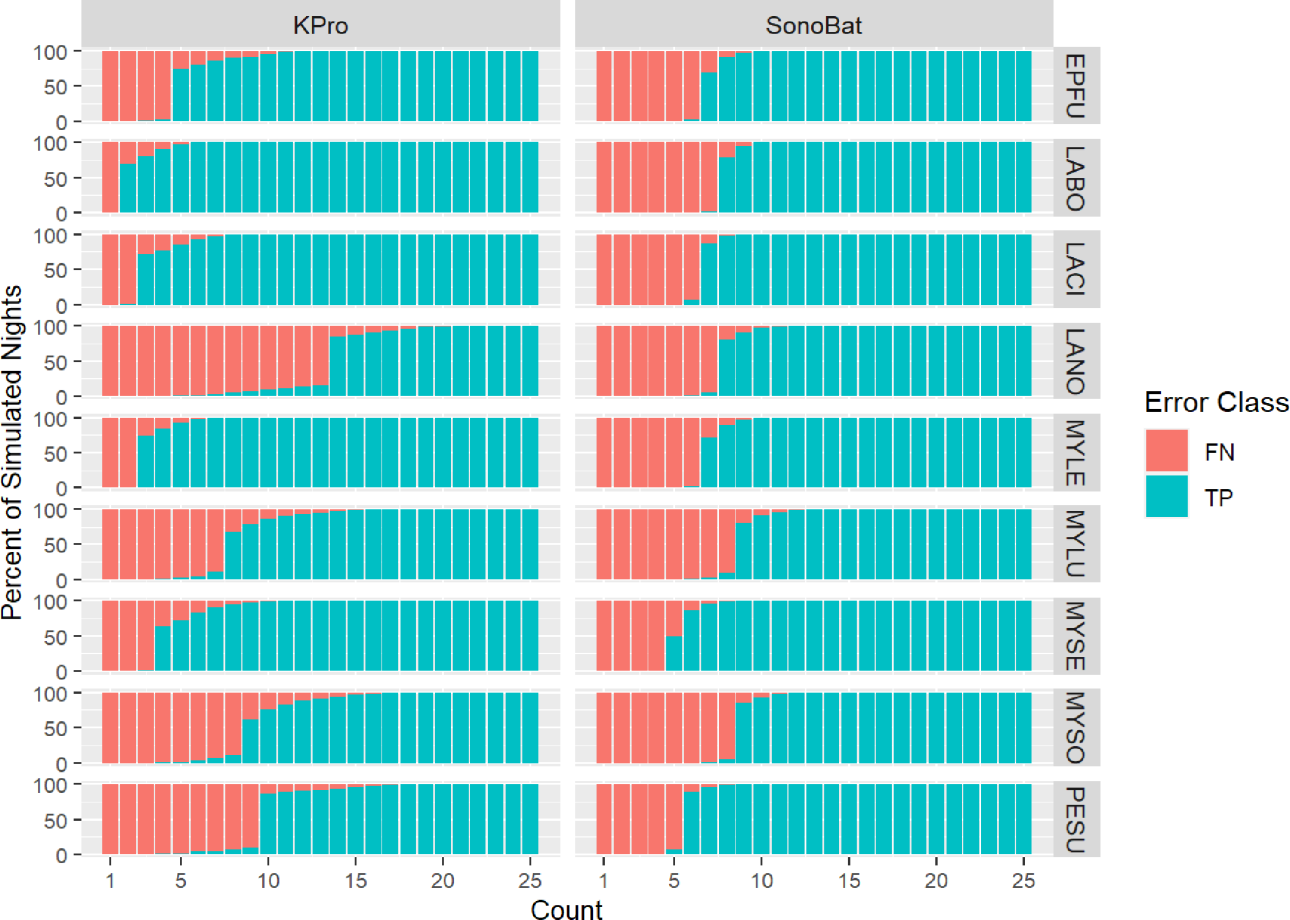
Percent of true positive (TP) and false negative (FN) simulated nights generated by Kaleidoscope Pro and SonoBat programs at different Counts for each species, using a MLE *P≤ * 0.05 threshold for acoustic presence. All Count categories were equal across species and had 225 observations each. EPFU *=  Eptesicus fuscus,* LABO =  *Lasiurus borealis*, LACI =  *Lasiurus cinereus*, LANO =  *Lasionycteris noctivagans*, MYLE =  *Myotis leibii*, MYLU =  *Myotis lucifugus*, MYSE =  *Myotis septentrionalis*, MYSO =  *Myotis sodalis*, PESU =  *Perimyotis subflavus*.

The proportion of simulated nights that Examined Species were correctly determined to be present (MLE *P* ≤  0.05; ‘true positives’) by each program increased with the number of Examined Species’ audio files included in the simulated night (i.e., ‘Count’; Fig 2). Overall, the average Count required to yield a high percentage of TP simulated nights was similar between KPro (mean =  6.6 audio files) and SonoBat (7.5), though it was more variable for KPro (standard deviation =  4.1) than SonoBat (1.1) among the nine species. KPro required much lower Counts than SonoBat to yield a high percentage of TP simulated night for three species: *L. borealis, L. cinereus,* and *M. leibii.* For example, to correctly determine presence of *L. borealis* on > 75% of simulated nights, KPro only required ≥  2 audio files of that species to be included in a simulated night, whereas SonoBat required ≥  8 audio files (Fig 2). In contrast, SonoBat required fewer audio files for *L. noctivagans* (8 files) and *P. subflavus* (6) than KPro (14 and 10, respectively). Counts only differed by one or two files for the remaining four species (Fig 2).

### Modeling

The best model was designated Ratio_Bats and included variables for the log_10_ of the Count and the ratio of the species’ Count to all other audio files (‘S11 Table 4.xlsx’). Many models had similar MAE and RMSE values (S6 File and S7 File). Due to our training simulated night dataset having mostly low species ratios (mean =  0.11, standard deviation =  0.05, S1 Fig) many of the more complex models tested suffered from overfitting at high and at very low species ratios. Therefore, we chose Ratio_Bats as our preferred model because it contained the fewest number of variables and improved performance over a large range of species ratios.

**Fig 3:**
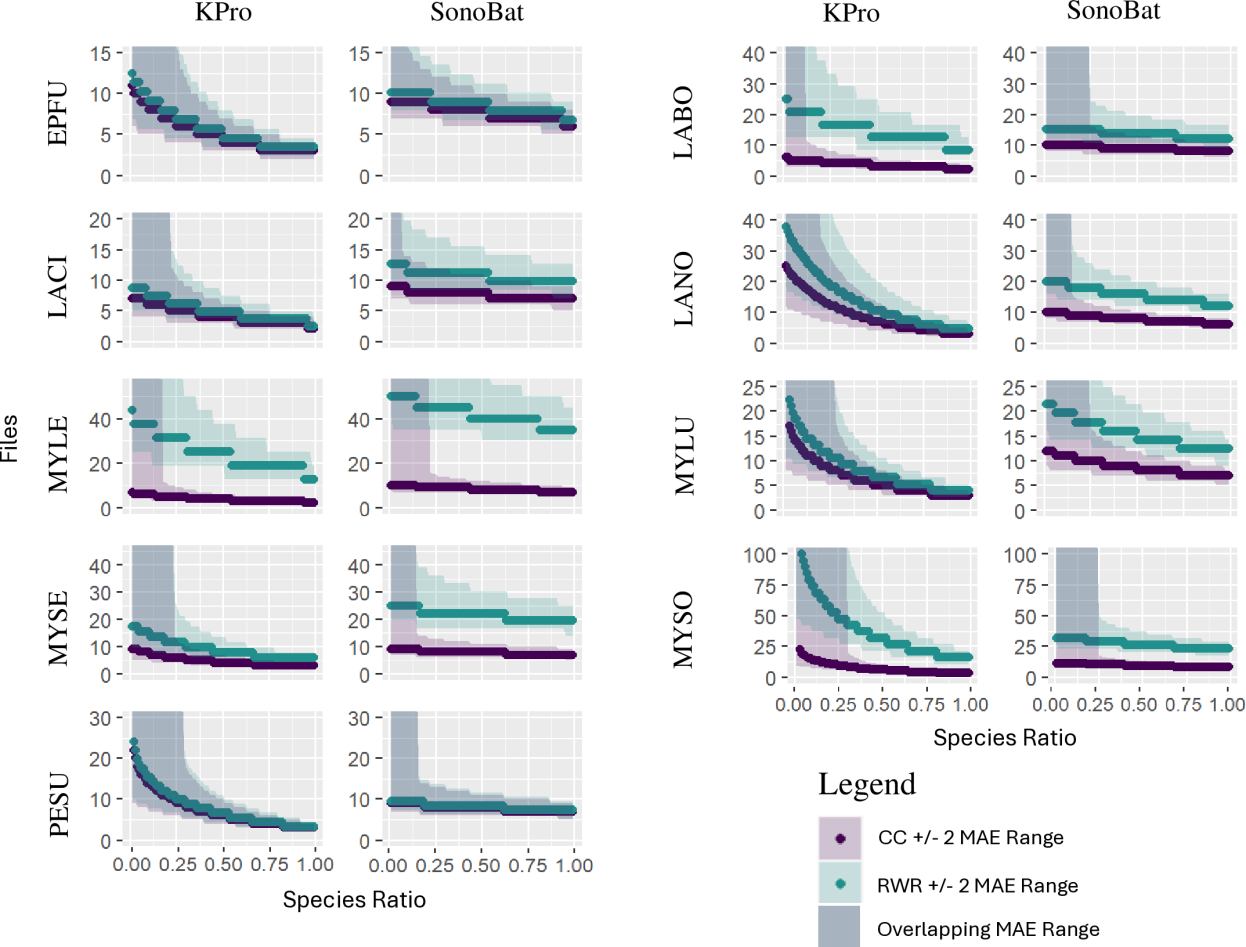
Model interpolations for Kaleidoscope Pro (KPro) and SonoBat of critical count (CC, purple) and real-world recordings (RWR, green) for a given species ratio of each Examined Species. Note that the upper limit on the y-axis is different between species. EPFU *=  Eptesicus fuscus,* LABO =  *Lasiurus borealis*, LACI =  *Lasiurus cinereus*, LANO =  *Lasionycteris noctivagans*, MYLE =  *Myotis leibii*, MYLU =  *Myotis lucifugus*, MYSE =  *Myotis septentrionalis*, MYSO =  *Myotis sodalis*, PESU =  *Perimyotis subflavus*.

The GLM models we created let us examine species counts and species ratios not included in the simulated nights dataset. Fig 3 shows modeled interpolations of CC and RWR needed by KPro and SonoBat to obtain MLE *P* ≤  0.05 for each species across a range of species ratios. Each data point represents a CC or RWR for a given species ratio and the shaded areas represent ±  2 MAE. Across species and programs, CC and RWR were low at high species ratio ( ≥ 0.75) and increased as the species ratio of the Examined Species decreased (Fig 3). In general, our model predicted KPro to have slightly lower CC than SonoBat for most species (except *L. noctivagans* and *P. subflavus*, consistent with Fig 2). For both programs, CC was higher for more ambiguous species, such as *M. sodalis*, than for more distinct species, such as *L. cinereus* (Fig 3). The RWR was nearly identical to CC across species ratios for four species analyzed by KPro (*E. fuscus, L. cinereus, M. lucifugus, P. subflavus*) and for two species analyzed by SonoBat (*E. fuscus* and*, P. subflavus*; Fig 3), reflecting high sensitivity for these species by these programs ( ≥ 0.76; [15], S1 Table). Both RWR and CC diverged greatly for *M. leibii* and *M. sodalis* for both programs. The disparity between RWR and CC was also great for *L. borealis* analyzed by KPro, and for *L. noctivagans, M. lucifugus,* and *M. septentrionalis* analyzed by SonoBat (Fig 3).

### Fort drum military installation case study

**Fig 4:**
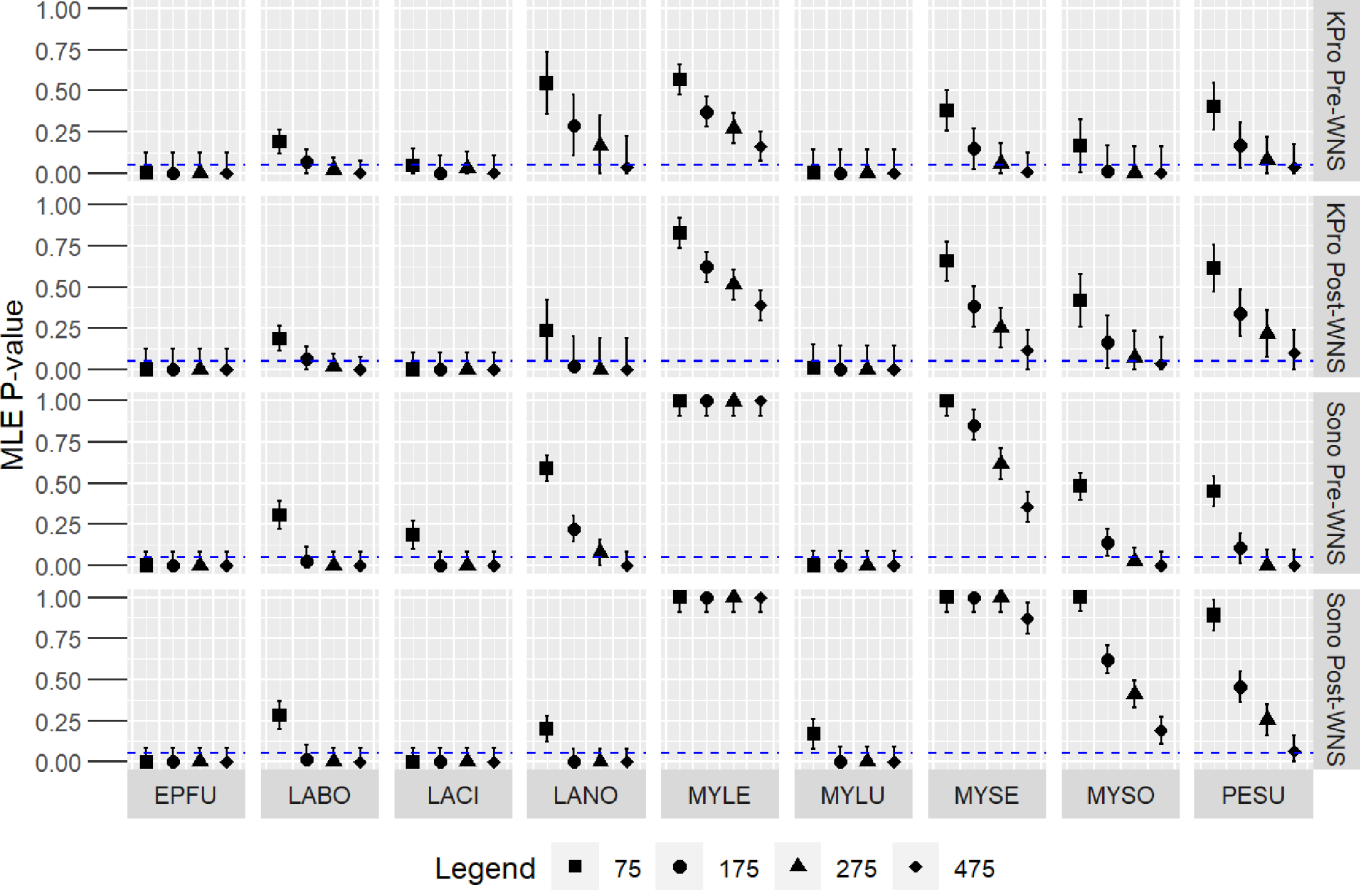
Model-predicted MLE values by Kaleidoscope Pro (KPro) and SonoBat (Sono) programs for northeastern bat species on nights with 75, 175, 275, and 475 total bat audio files using species counts and species ratios during pre- and post-White Nose Syndrome (WNS) periods at the Fort Drum Military Installation. Scatter plot error bars show ±  two MAE. Blue dashed line shows MLE =  0.05. EPFU =  *Eptesicus fuscus*, LABO *=  Lasiurus borealis*, LACI =  *Lasiurus cinereus*, LANO =  *Lasionycteris noctivagans*, MYLE =  *Myotis leibii*, MYLU *=  Myotis lucifugus*, MYSE =  *Myotis septentrionalis*, MYSO =  *Myotis sodalis*, PESU =  *Perimyotis subflavus*.

In our Fort Drum Military Installation simulations, MLE presence for a species was more likely if that species was relatively common (e.g., *E. fuscus, L. cinereus*) and less likely if the species was relatively rare (e.g., *M. leibeii, M. septentrionalis*; Fig 4; Table 1). As expected, the MLE *P-*value of species decreased as the number of total bat audio files increased (e.g., *L. noctivagans, P. subflavus*; Fig 4; Table 1).

**Table 1:** Species ratios (bold; as percentages) at Fort Drum Military Installation, New York, during pre- and post-White Nose Syndrome periods derived from Nocera et al. (2019) and used to generate models used in Fig 4. ‘Count’ indicates the number of files input to our model for each species and was derived by multiplying species ratio by the total number of files in the simulation (75, 175, 275, or 475). Fractional counts better reflect abundances despite the impossibility of fractional call files.

		EPFU	LABO	LACI	LANO	MYLE	MYLU	MYSE	MYSO	PESU
**Pre-WNS Species ratio**		**0.216**	**0.092**	**0.088**	**0.055**	**0.003**	**0.490**	**0.007**	**0.009**	**0.041**
Count	**75**	16.2	6.9	6.6	4.1	0.2	36.7	0.5	0.7	3.1
	**175**	37.8	16.1	15.4	9.7	0.5	85.7	1.2	1.5	7.2
	**275**	59.4	25.3	24.1	15.2	0.8	134.7	1.9	2.4	11.2
	**475**	102.7	43.6	41.7	26.3	1.3	232.6	3.2	4.2	19.4
**Post-WNS Species ratio**		**0.246**	**0.097**	**0.332**	**0.136**	**0.001**	**0.157**	**0.003**	**0.011**	**0.017**
Count	**75**	18.4	7.3	24.9	10.2	0.1	11.8	0.2	0.8	1.3
	**175**	43.0	17.0	58.1	23.8	0.2	27.5	0.5	1.9	3.0
	**275**	67.6	26.7	91.3	37.4	0.3	43.3	0.8	2.9	4.8
	**475**	116.7	46.1	157.7	64.5	0.6	74.7	1.4	5.0	8.3

EPFU =  Eptesicus fuscus, LABO =  Lasiurus borealis, LACI =  Lasiurus cinereus, LANO =  Lasionycteris noctivagans, MYLE =  Myotis leibii, MYLU =  Myotis lucifugus, MYSE =  Myotis septentrionalis, MYSO =  Myotis sodalis, PESU =  Perimyotis subflavus.

The MLE *P-*values were very similar between pre and post-WNS within programs for most species (e.g., *L. borealis*, *L. noctivagans*; Fig 4). KPro was more likely than SonoBat to correctly determine acoustic presence (MLE *P* ≤  0.05) of rare, acoustically ambiguous species, such as *M. septentrionalis* and *M. sodalis*, though KPro still required many total bat audio files to do so (Fig 4). Post-WNS, both programs performed better for correctly determining presence of *L. noctivagans*, whose species ratio doubled between pre- and post-WNS periods, and performed worse for *P. subflavus,* whose species ratio halved between periods (Fig 4; Table 1). Despite the large decrease in species ratio of *M. lucifugus* at Fort Drum between periods, KPro reliably detected *M. lucifugus* at pre and post-WNS abundances whereas SonoBat had decreased performance post-WNS when the total count of files was 75 (Fig 4; Table 1).

## Discussion

Federal guidelines for determining presence of endangered bat species from acoustic monitoring were first established in 2014 and are largely contingent upon MLE *P*-values derived from automated classification programs during individual nights of sampling [26]. These programs are routinely tested to ensure that they accurately report acoustic presence for *M. septentrionalis* and *M. sodalis* (and potentially *P. subflavus* in coming years) under simulated conditions [21]. Results from our own simulations indicate that calculation of correct MLE *P*-values by KPro and SonoBat for these and other northeastern US species is highly dependent on the a) count of audio files for a species of interest; b) species ratio of audio files for a species of interest; and c) total number of audio files that were collected within a single night of monitoring. While KPro and SonoBat are capable of reporting acoustic presence of species using the federal methodology, we find that the number of recordings required for accurate presence may be difficult to achieve, particularly for rare species recorded under real-world conditions.

Similar to observations of others [16,17], we found that the likelihood a species is acoustically present on a night increased as the number of audio files for that species (or Count) was increased. Both programs reported false negatives for all species at Counts of a single audio file, indicating that automated classifiers can struggle to correctly determine presence for a species when it is rare in a sample. The programs performed similarly for *E. fuscus, M. lucifugus, M. septentrionalis,* and *M. sodalis*, but required very different Counts to determine acoustic presence of the remaining five species. Our simulations only used species files that had previously been correctly identified by programs [15], so differences between programs reflect differences in their MLE algorithms, not in their file identification algorithms. For example, SonoBat required a minimum of 8 *L. borealis* files to determine acoustic presence of this species whereas KPro required a minimum of 2 files, even though SonoBat has much higher sensitivity for this species (0.92) compared to KPro (0.24; [15]). Therefore, the accuracy of a program’s file identification algorithm does not necessarily reflect the performance of its MLE algorithm.

Neither program was able to achieve reliable TPs ( > 90% of simulated nights) for *L. noctivagans* until approximately 8-14 files from the species were included in the sample. This likely reflects a high overlap in parameters with *E. fuscus* [27]. KPro and SonoBat were also conservative for *M. lucifugus* and *M. sodalis*, not providing reliable TPs until simulated nights contained 8 to 10 files from these species. SonoBat has not yet been approved for acoustic surveys of *M. septentrionalis* and *M. sodalis* because the Master Test Library maintained by the USFWS is just now acquiring full-spectrum calls for testing this program (A. King, pers comm). Based on our results, we predict that SonoBat (using default settings) should perform similar to KPro (using USFWS-recommended settings) on USFWS tests for these *Myotis* species. On the other hand, our simulations indicate that SonoBat required approximately half the number of calls from *P. subflavus* to establish acoustic presence compared to KPro, despite both programs having high sensitivity for this species (0.97 and 0.91, respectively; [15]). We predict that SonoBat will require fewer files than KPro to establish presence of *P. subflavus* when the USFWS begins testing programs for this species.

Our modeling exercise illustrates the potential limitations for using MLE to detect the presence of rare species via acoustic monitoring. Modelled predictions of CC and RWR for Examined Species across a range of species ratios found that, in general, the number of audio files needed to establish acoustic presence for a species increased as species ratio of that species decreased within a sample. Values for CC and RWR were nearly identical across the range of species ratios for species with high sensitivity (*E. fuscus, P. subflavus*; [15]*,* S1 Table) while the number of RWR needed to determine acoustic presence was much greater than CC for species with low sensitivity, particularly at low species ratio (*M. leibii, M. sodalis*).

Uncertainty for CC and RWR became very high for most Examined Species when their species ratios were less than 25%. This pattern may be an effect of using log_10_ to describe Count in our model. The species ratio coefficients are negative, so MLE increases as species ratio decreases (see coefficient estimates in S6 File and S7 File). The Count term is also negative and, if species ratio is constant, the only way to decrease MLE to 0.05 is to increase Count. At higher Count values, increases in Count have less effect on MLE due to the log_10_ term. That is, the change in MLE at Counts 1-2 is greater than the change in MLE at Counts 24-25. Therefore, a larger increase in Count is needed to achieve MLE *P* ≤  0.05 at lower species ratios. This mathematical relationship may hint that at lower Counts the effect of variables not included in our model (such as the species ratio of other species with similar echolocation calls) have a larger impact on MLE. Since our model does not include those specific variables, their effect comes out in the error term.

Our results are consistent with Ford et al. 2024 [16] that found KPro required > 15 files to calculate MLE *P* <  0.05 for *M. septentrionalis* and *M. sodalis* when these species were at low proportion to other high-frequency species. These results suggest that using MLE to determine acoustic presence for rare species may be unreliable in some instances. Recording enough high-quality echolocation calls from rare species to establish accurate acoustic presence may be difficult in most parts of their range. This issue is exacerbated for *M. septentrionalis*, a gleaning species that produces low-intensity echolocation calls and is difficult to detect relative to other bats [28,29].

The difficulty of detecting rare species may be increased if relatively few total bats are recorded within a night. Applying our model to historical acoustic data from the Fort Drum Military Installation revealed that the species declared present generated by KPro and SonoBat for the site were quite different when 75 files were examined (three and two species, respectively) compared to 475 files (eight and six species, respectively). That is, even at unrealistically high total numbers of bats calls, neither program was able to detect presence for the full suite of nine species at Fort Drum. These results suggest the total number of bat files recorded during a night may be more important for determining accurate species inventories than the program used, which has implications for surveying rare species in low activity areas. For example, of 50 pre-construction wind energy development areas monitored for bat use, none exceeded an average bat activity rate of 15 passes per detector-night [30], suggesting that using MLE values to determine acoustic presence of bat species at proposed wind energy facilities may be inadequate for all but the most common species. The risk for false negatives is higher when the total number of bat files recorded within a night is low. During presence/absence surveys, we recommend that researchers place ultrasonic detectors at or near features attractive to bats (e.g., forest edges and water features) to maximize the total number of bat files recorded each night, and that this number be reported to the USFWS so that MLE *P-*values can be fully evaluated.

Remarkably, despite differences in accuracy [15], proportion of false negative nights, and the number of files required to establish acoustic presence, KPro and SonoBat performed similarly for determining acoustic presence of non-*Myotis* species at Fort Drum when the file classification and MLE algorithms are combined*.* This was true across pre- and post-WNS periods and across total number of files sampled in the Fort Drum data. For example, both programs indicated *L. borealis* was absent when total sample size was 75 files but indicated presence when sample size was 175 files or greater.

Differences between programs were more nuanced for *Myotis* species. Both programs failed to determine presence for *M. leibii* at any sample size, demonstrating the limitation of these programs to detect species that compose < 1% of a population. For the other three *Myotis* species we tested, KPro was more likely to establish acoustic presence for *M. lucifugus, M. septentrionalis,* and *M. sodalis* at lower numbers of total files than SonoBat. Interestingly, KPro was able to accurately determine acoustic presence of *M. sodalis* with just 1-5 files, which was far fewer than the ~10 files predicted by our data (Fig 2). This was likely driven by misclassification of *M. lucifugus* as *M. sodalis* (i.e., false positives). The species ratio of *M. sodalis* did not change between periods (1%), while the abundance of *M. lucifugus* drastically declined from 49% to 16% after WNS. Misclassification of *M. lucifugus* as *M. sodalis* allowed the total positive (true positives +  false positives) count for *M. sodalis* to reach MLE *P* ≤  0.05 only when the *M. lucifugus* count was high during pre-WNS. A similar ‘swamping effect’ was observed by [16] when nightly counts of target species were low. Our models are imperfect and were designed to measure the false negative rate of the programs, not the false positive rates. The programs may have more sophisticated means to reduce false positives not captured in our models. Therefore, we cannot say with confidence that misclassified files contribute true positives for rare species as they did in our models.

Considering the difficulties for establishing acoustic presence of rare species using MLE presented here, we argue that alternative approaches should be considered. Rather than determining species presence using the audio files recorded by a single detector on individual nights, one could determine presence using all audio files recorded by a single detector for all nights or using the audio files recorded by all detectors for individual nights. Both options would increase the total number of bat files examined by the MLE algorithm and increase the likelihood of detecting true presence of rare species. Another possibility could be raising the USFWS MLE *P-*value threshold for presence from 0.05 to a higher number. However, our Fort Drum modeling suggests this may not be effective. For the scenarios we tested, an MLE threshold of 0.10 would have still missed many rare species. Additionally, increasing the MLE threshold may increase the risk of false positives.

A better solution might involve manual review, or visual inspection, of files flagged as possible rare or endangered species by automated software. The current guidelines require that all files be visually reviewed for any nights that MLE *P* ≤  0.05 for *M. septentrionalis*, *M. sodalis* or *P. subflavus* [11]. However, our results indicate that relatively low numbers of files for these species (or low total numbers of files) may result in nights with MLE *P* >  0.05, or false absence. To overcome this issue and provide a more conservative measure of presence, we suggest that any files classified as these three species be visually reviewed by one or more bat acoustic experts. Owing to the rarity of protected species, visually reviewing files flagged by software should not be overly burdensome [16,17], and while manual review is more subjective than automated classification, manual classifications can be defended.

If a more objective, automated approach is preferred, then another option could be to have programs calculate MLE for *Myotis* as a whole. Solick et al. (2024) [15] found that programs were more accurate at correctly classifying calls to the genus *Myotis* than to individual *Myotis* species. Perhaps triggering manual review of all files when MLE *P* ≤  0.05 for any *Myotis* would be more likely to capture echolocation calls produced by individual *Myotis* species.

Lastly, there are other possible approaches for determining acoustic presence instead of MLE (Review Article:[31]). Promising work has been done incorporating a multivariable Bayesian framework [32] which has the potential to improve bat inventories and expand knowledge of important environmental variables influencing bat activity.

The results from our simulations and modelling are based on the quality of the echolocation calls chosen for these tests, the specific versions of the programs used, and a species composition specific to just six northeastern US states. Our model is a useful tool but was simple and did not account for false positives or the species ratios of other species. As well, the species ratios of bat species are often unknown prior to a study. We therefore caution the use of specific numbers of files required to trigger MLE *P* ≤  0.05 that we reported here, and we encourage further testing and simulation work in areas with different species composition and species ratios. Areas with different species ratio of these nine northeastern species, or areas with different species altogether, will yield different results. But we do believe the influence of count and species ratio on MLE/acoustic presence will be consistent and should be considered when designing bat acoustic species surveys and interpreting the results of those surveys.

Our study shows that while automated bat echolocation classification is an impressive and time-saving tool, it is not reliable for detecting rare species. Relatively large numbers of total bat echolocation recordings are needed to detect species with relatively low abundances. Reevaluation of current MLE thresholds and methodologies used to determine species presence/absence may be warranted, particularly when making species-specific conservation, regulatory, and permitting decisions.

## Supporting Information

S1 Table Sensitivity Matrix for SonoBat version used in this study. (XLSX)

S2 Table Confusion Matrix for SonoBat version used in this study (XLSX)

S3 Table Number of files by species each program correctly classified. (XLSX)

S4 Table Explanation of variables used in modeling. (XLSX)

S1 Fig Distribution of species ratios in simulated nights. (PDF)

S1 Script R script used to create simulated nights for SonoBat. (R)

S2 Script R script used to create simulated nights for KPro. (R)

S3 Script R script used to analyze simulated nights for SonoBat. (R)

S4 Script R script used to analyze simulated nights for KPro. (R)

S5 Script R script used to visualize SonoBat and KPro analyses. (R)

S1 File Output of S3 Script. Contains metrics describing simulated nights and models tested for Sono Bat.
(HTML)

S2 File Output of S4 Script. Contains metrics describing simulated nights and models tested for KPro.
(HTML)
