# Supplementary material for: Maximum likelihood estimators are ineffective for acoustic detection of rare bat species
Source: PLoS One. 2025 Apr 1;20(4):e0320646. doi: 10.1371/journal.pone.0320646 (PMC11960983; doi:10.1371/journal.pone.0320646)
Supplement: S1 Fig — (PDF) [file pone.0320646.s005.pdf]

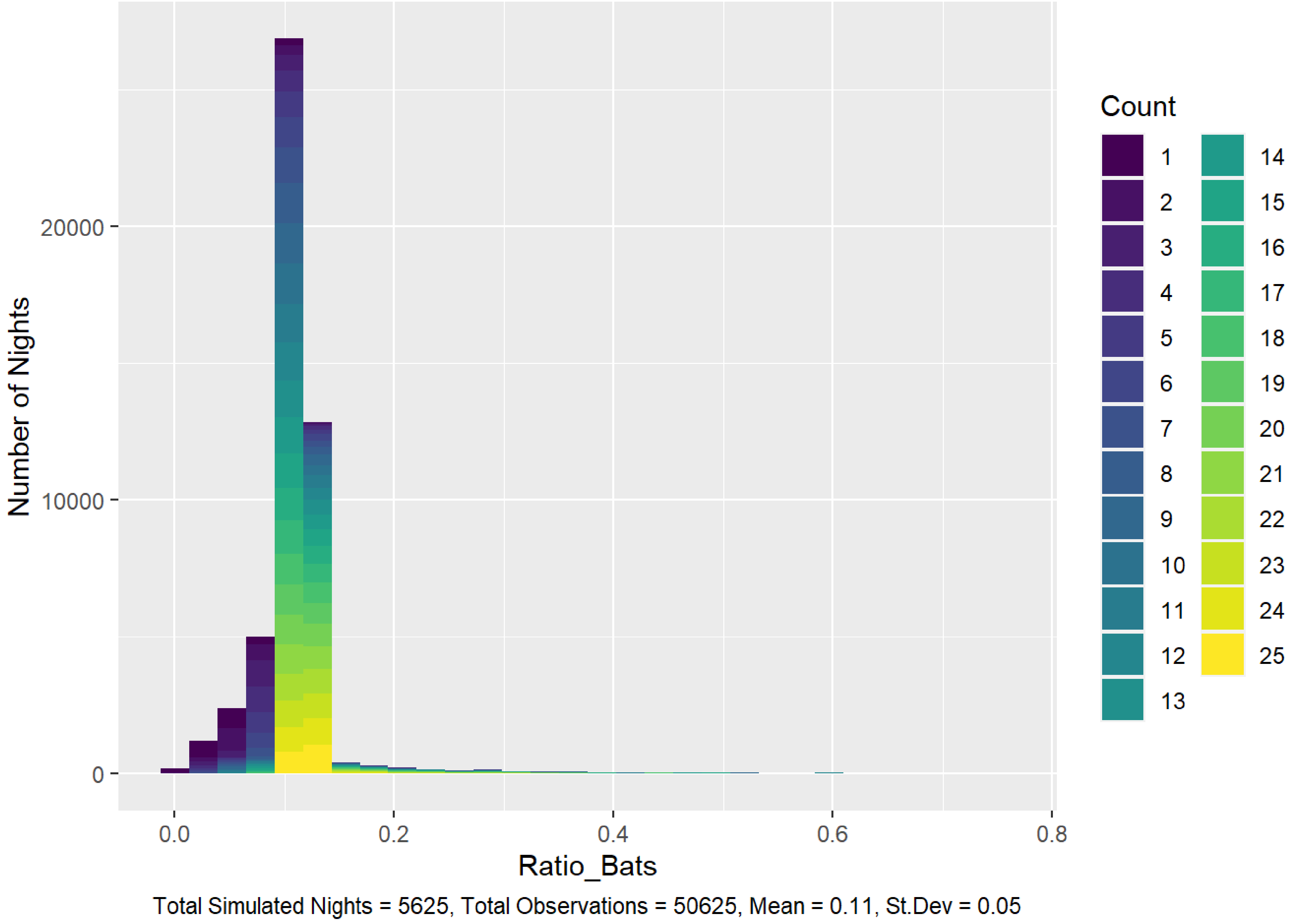

Supplementary Figure 1: Distribution of species ratios in simulated nights.

Ratio_Bats (x-axis) shows the species ratio bins for simulated nights in this study. Number of Observations (y-axis) shows how many observations fall within each bin. Color code ‘Count’ indicates how many audio files were present for the Examined Species in each observation. All possible Examined Species are included in this plot. Divide ‘Number of Observations’ by the number of species (9) to find the Number of Observations for an individual Examined Species.
