# Supplementary material for: Maximum likelihood estimators are ineffective for acoustic detection of rare bat species
Source: PLoS One. 2025 Apr 1;20(4):e0320646. doi: 10.1371/journal.pone.0320646 (PMC11960983; doi:10.1371/journal.pone.0320646)
Supplement: S1 File — Contains metrics describing simulated nights and models tested for Sono Bat. (HTML) [file pone.0320646.s011.html]

SonoBat MLE


Code 

- Show All Code
- Hide All Code

# SonoBat MLE

#### Bradley Hopp

#### 2025-02-25

# Subset Description

# Abbreviations

| Abbreviation | Scientific Name |
| --- | --- |
| EPFU | Eptesicus fuscus |
| LABO | Lasiurus borealis |
| LACI | Lasiurus cinereus |
| LANO | Lasionycteris noctivagans |
| MYLE | Myotis leibii |
| MYLU | Myotis lucifugus |
| MYSE | Myotis septentrionalis |
| MYSO | Myotis sodalis |
| PESU | Perimyotis subflavus |

# MLE v Count

Horizontal, dashed blue line reveals MLE = 0.05. Datapoints represent
simulated nights. Y-axis, ‘MLE’, is software MLE. X-axis, ‘Count’, is
the number of species files
present.

# MLE v Species Ratio

Horizontal, dashed blue line reveals MLE = 0.05. Datapoints represent
simulated nights. Y-axis, ‘MLE’, is software MLE. X-axis is Ratio\_Bats.
Different frames represent different file Counts.

### *EPFU*

### *LABO*

### *LACI*

### *LANO*

### *MYLE*

### *MYLU*

### *MYSE*

### *MYSO*

### *PESU*

# Models

## GLM Coefs

Adj R2 is R score but all model MLE’s > 1 are set to one and < 0
are set to 0.

Coefficient Estimates

| Vars | Species | (Intercept) | Ex\_Count | log\_Ex\_Count | Ratio\_Bats | log\_Ratio\_Bats | Ex\_Count\_Ratio\_Bats | sq\_Ratio\_Bats | Ratio\_High/Low | log\_Ratio\_High/Low | Ex\_Count\_Ratio\_High/Low | sq\_Ratio\_High/Low | Ratio\_EPFU | Ratio\_LABO | Ratio\_LACI | Ratio\_LANO | Ratio\_MYLE | Ratio\_MYLU | Ratio\_MYSE | Ratio\_MYSO | Ratio\_PESU | log\_Ratio\_EPFU | log\_Ratio\_LABO | log\_Ratio\_LACI | log\_Ratio\_LANO | log\_Ratio\_MYLE | log\_Ratio\_MYLU | log\_Ratio\_MYSE | log\_Ratio\_MYSO | log\_Ratio\_PESU | Ex\_Count\_Ratio\_EPFU | Ex\_Count\_Ratio\_LABO | Ex\_Count\_Ratio\_LACI | Ex\_Count\_Ratio\_LANO | Ex\_Count\_Ratio\_MYLE | Ex\_Count\_Ratio\_MYLU | Ex\_Count\_Ratio\_MYSE | Ex\_Count\_Ratio\_MYSO | Ex\_Count\_Ratio\_PESU | sq\_Ratio\_EPFU | sq\_Ratio\_LABO | sq\_Ratio\_LACI | sq\_Ratio\_LANO | sq\_Ratio\_MYLE | sq\_Ratio\_MYLU | sq\_Ratio\_MYSE | sq\_Ratio\_MYSO | sq\_Ratio\_PESU | Adj\_R2 | R2 | MAE | RMSE |
| --- | --- | --- | --- | --- | --- | --- | --- | --- | --- | --- | --- | --- | --- | --- | --- | --- | --- | --- | --- | --- | --- | --- | --- | --- | --- | --- | --- | --- | --- | --- | --- | --- | --- | --- | --- | --- | --- | --- | --- | --- | --- | --- | --- | --- | --- | --- | --- | --- | --- | --- | --- | --- |
| Ratio\_Bats | EPFU | 0.9606 | 0.0371 | -0.5667 | -0.1262 | NA | NA | NA | NA | NA | NA | NA | NA | NA | NA | NA | NA | NA | NA | NA | NA | NA | NA | NA | NA | NA | NA | NA | NA | NA | NA | NA | NA | NA | NA | NA | NA | NA | NA | NA | NA | NA | NA | NA | NA | NA | NA | NA | 0.9527 | 0.9460 | 0.0407 | 0.0576 |
| log\_Ratio\_Bats | EPFU | 0.5993 | 0.0351 | -0.5317 | 0.4017 | -0.1091 | NA | NA | NA | NA | NA | NA | NA | NA | NA | NA | NA | NA | NA | NA | NA | NA | NA | NA | NA | NA | NA | NA | NA | NA | NA | NA | NA | NA | NA | NA | NA | NA | NA | NA | NA | NA | NA | NA | NA | NA | NA | NA | 0.9613 | 0.9524 | 0.0389 | 0.0541 |
| INT\_log\_Ratio\_Bats | EPFU | 0.6192 | 0.0344 | -0.5301 | 0.2943 | -0.1045 | 0.0050 | NA | NA | NA | NA | NA | NA | NA | NA | NA | NA | NA | NA | NA | NA | NA | NA | NA | NA | NA | NA | NA | NA | NA | NA | NA | NA | NA | NA | NA | NA | NA | NA | NA | NA | NA | NA | NA | NA | NA | NA | NA | 0.9613 | 0.9525 | 0.0389 | 0.0541 |
| sq\_Ratio\_Bats | EPFU | 0.9960 | 0.0364 | -0.5505 | -0.8788 | NA | NA | 1.2517 | NA | NA | NA | NA | NA | NA | NA | NA | NA | NA | NA | NA | NA | NA | NA | NA | NA | NA | NA | NA | NA | NA | NA | NA | NA | NA | NA | NA | NA | NA | NA | NA | NA | NA | NA | NA | NA | NA | NA | NA | 0.9565 | 0.9487 | 0.0403 | 0.0562 |
| INT\_sq\_Ratio\_Bats | EPFU | 1.0070 | 0.0330 | -0.5413 | -1.0433 | NA | 0.0225 | 0.8887 | NA | NA | NA | NA | NA | NA | NA | NA | NA | NA | NA | NA | NA | NA | NA | NA | NA | NA | NA | NA | NA | NA | NA | NA | NA | NA | NA | NA | NA | NA | NA | NA | NA | NA | NA | NA | NA | NA | NA | NA | 0.9570 | 0.9492 | 0.0401 | 0.0559 |
| Ratio\_High/Low | EPFU | 1.0171 | 0.0374 | -0.5672 | 0.2812 | NA | NA | NA | -0.3154 | NA | NA | NA | NA | NA | NA | NA | NA | NA | NA | NA | NA | NA | NA | NA | NA | NA | NA | NA | NA | NA | NA | NA | NA | NA | NA | NA | NA | NA | NA | NA | NA | NA | NA | NA | NA | NA | NA | NA | 0.9592 | 0.9501 | 0.0405 | 0.0554 |
| log\_Ratio\_High/Low | EPFU | 0.6855 | 0.0357 | -0.5446 | -0.1018 | NA | NA | NA | 0.2821 | -0.1264 | NA | NA | NA | NA | NA | NA | NA | NA | NA | NA | NA | NA | NA | NA | NA | NA | NA | NA | NA | NA | NA | NA | NA | NA | NA | NA | NA | NA | NA | NA | NA | NA | NA | NA | NA | NA | NA | NA | 0.9641 | 0.9534 | 0.0387 | 0.0536 |
| INT\_log\_Ratio\_High/Low | EPFU | 0.7774 | 0.0324 | -0.5417 | -0.1341 | NA | NA | NA | 0.0975 | -0.0992 | 0.0091 | NA | NA | NA | NA | NA | NA | NA | NA | NA | NA | NA | NA | NA | NA | NA | NA | NA | NA | NA | NA | NA | NA | NA | NA | NA | NA | NA | NA | NA | NA | NA | NA | NA | NA | NA | NA | NA | 0.9643 | 0.9536 | 0.0389 | 0.0535 |
| sq\_Ratio\_High/Low | EPFU | 1.1218 | 0.0356 | -0.5349 | -0.6578 | NA | NA | NA | -0.9478 | NA | NA | 1.3375 | NA | NA | NA | NA | NA | NA | NA | NA | NA | NA | NA | NA | NA | NA | NA | NA | NA | NA | NA | NA | NA | NA | NA | NA | NA | NA | NA | NA | NA | NA | NA | NA | NA | NA | NA | NA | 0.9651 | 0.9552 | 0.0390 | 0.0526 |
| INT\_sq\_Ratio\_High/Low | EPFU | 1.1219 | 0.0354 | -0.5348 | -0.6543 | NA | NA | NA | -0.9441 | NA | 0.0006 | 1.3224 | NA | NA | NA | NA | NA | NA | NA | NA | NA | NA | NA | NA | NA | NA | NA | NA | NA | NA | NA | NA | NA | NA | NA | NA | NA | NA | NA | NA | NA | NA | NA | NA | NA | NA | NA | NA | 0.9651 | 0.9552 | 0.0390 | 0.0526 |
| Ratio\_SPECIES | EPFU | 0.9637 | 0.0366 | -0.5605 | -0.3553 | NA | NA | NA | NA | NA | NA | NA | NA | 0.0015 | 0.0012 | 0.0001 | 0.0018 | 0.0018 | 0.0012 | 0.0018 | 0.0018 | NA | NA | NA | NA | NA | NA | NA | NA | NA | NA | NA | NA | NA | NA | NA | NA | NA | NA | NA | NA | NA | NA | NA | NA | NA | NA | NA | 0.9536 | 0.9466 | 0.0407 | 0.0574 |
| log\_Ratio\_SPECIES | EPFU | 0.8903 | 0.0369 | -0.5570 | 0.2349 | NA | NA | NA | NA | NA | NA | NA | NA | 0.0006 | 0.0025 | 0.0087 | -0.0014 | -0.0014 | 0.0025 | -0.0014 | -0.0014 | NA | -0.0084 | -0.0174 | -0.0472 | 0.0007 | 0.0007 | -0.0174 | 0.0007 | 0.0007 | NA | NA | NA | NA | NA | NA | NA | NA | NA | NA | NA | NA | NA | NA | NA | NA | NA | NA | 0.9643 | 0.9539 | 0.0397 | 0.0535 |
| INT\_log\_Ratio\_SPECIES | EPFU | 0.8794 | 0.0376 | -0.5578 | 0.2110 | NA | NA | NA | NA | NA | NA | NA | NA | 0.0023 | 0.0044 | 0.0109 | -0.0001 | -0.0001 | 0.0044 | -0.0001 | -0.0001 | NA | -0.0090 | -0.0181 | -0.0481 | 0.0003 | 0.0003 | -0.0181 | 0.0003 | 0.0003 | NA | -1e-04 | -0.0001 | -1e-04 | -1e-04 | -0.0001 | -1e-04 | -0.0001 | -1e-04 | NA | NA | NA | NA | NA | NA | NA | NA | NA | 0.9644 | 0.9540 | 0.0397 | 0.0535 |
| sq\_Ratio\_SPECIES | EPFU | 0.9615 | 0.0365 | -0.5586 | -0.4496 | NA | NA | NA | NA | NA | NA | NA | NA | 0.0032 | 0.0013 | -0.0052 | 0.0050 | 0.0050 | 0.0013 | 0.0050 | 0.0050 | NA | NA | NA | NA | NA | NA | NA | NA | NA | NA | NA | NA | NA | NA | NA | NA | NA | NA | NA | -1e-04 | 0e+00 | 3e-04 | -2e-04 | -2e-04 | 0e+00 | -2e-04 | -2e-04 | 0.9539 | 0.9468 | 0.0407 | 0.0573 |
| INT\_sq\_Ratio\_SPECIES | EPFU | 0.9702 | 0.0351 | -0.5545 | -0.4801 | NA | NA | NA | NA | NA | NA | NA | NA | 0.0028 | -0.0014 | -0.0155 | 0.0068 | 0.0068 | -0.0014 | 0.0068 | 0.0068 | NA | NA | NA | NA | NA | NA | NA | NA | NA | NA | 1e-04 | 0.0002 | 8e-04 | -1e-04 | -0.0001 | 2e-04 | -0.0001 | -1e-04 | NA | -1e-04 | -1e-04 | 0e+00 | -1e-04 | -1e-04 | -1e-04 | -1e-04 | -1e-04 | 0.9549 | 0.9474 | 0.0409 | 0.0571 |
| Ratio\_Bats | LABO | 1.0358 | 0.0349 | -0.5797 | -0.0845 | NA | NA | NA | NA | NA | NA | NA | NA | NA | NA | NA | NA | NA | NA | NA | NA | NA | NA | NA | NA | NA | NA | NA | NA | NA | NA | NA | NA | NA | NA | NA | NA | NA | NA | NA | NA | NA | NA | NA | NA | NA | NA | NA | 0.9590 | 0.9480 | 0.0422 | 0.0612 |
| log\_Ratio\_Bats | LABO | 0.8522 | 0.0339 | -0.5620 | 0.1838 | -0.0555 | NA | NA | NA | NA | NA | NA | NA | NA | NA | NA | NA | NA | NA | NA | NA | NA | NA | NA | NA | NA | NA | NA | NA | NA | NA | NA | NA | NA | NA | NA | NA | NA | NA | NA | NA | NA | NA | NA | NA | NA | NA | NA | 0.9609 | 0.9494 | 0.0418 | 0.0604 |
| INT\_log\_Ratio\_Bats | LABO | 0.9032 | 0.0320 | -0.5578 | -0.0914 | -0.0437 | 0.0128 | NA | NA | NA | NA | NA | NA | NA | NA | NA | NA | NA | NA | NA | NA | NA | NA | NA | NA | NA | NA | NA | NA | NA | NA | NA | NA | NA | NA | NA | NA | NA | NA | NA | NA | NA | NA | NA | NA | NA | NA | NA | 0.9610 | 0.9496 | 0.0419 | 0.0603 |
| sq\_Ratio\_Bats | LABO | 1.0578 | 0.0345 | -0.5697 | -0.5525 | NA | NA | 0.7785 | NA | NA | NA | NA | NA | NA | NA | NA | NA | NA | NA | NA | NA | NA | NA | NA | NA | NA | NA | NA | NA | NA | NA | NA | NA | NA | NA | NA | NA | NA | NA | NA | NA | NA | NA | NA | NA | NA | NA | NA | 0.9602 | 0.9489 | 0.0422 | 0.0607 |
| INT\_sq\_Ratio\_Bats | LABO | 1.0666 | 0.0318 | -0.5622 | -0.6850 | NA | 0.0181 | 0.4863 | NA | NA | NA | NA | NA | NA | NA | NA | NA | NA | NA | NA | NA | NA | NA | NA | NA | NA | NA | NA | NA | NA | NA | NA | NA | NA | NA | NA | NA | NA | NA | NA | NA | NA | NA | NA | NA | NA | NA | NA | 0.9603 | 0.9492 | 0.0421 | 0.0605 |
| Ratio\_High/Low | LABO | 1.0527 | 0.0351 | -0.5803 | 0.4683 | NA | NA | NA | -0.4751 | NA | NA | NA | NA | NA | NA | NA | NA | NA | NA | NA | NA | NA | NA | NA | NA | NA | NA | NA | NA | NA | NA | NA | NA | NA | NA | NA | NA | NA | NA | NA | NA | NA | NA | NA | NA | NA | NA | NA | 0.9600 | 0.9490 | 0.0422 | 0.0606 |
| log\_Ratio\_High/Low | LABO | 0.8787 | 0.0341 | -0.5649 | 0.0445 | NA | NA | NA | 0.0947 | -0.0557 | NA | NA | NA | NA | NA | NA | NA | NA | NA | NA | NA | NA | NA | NA | NA | NA | NA | NA | NA | NA | NA | NA | NA | NA | NA | NA | NA | NA | NA | NA | NA | NA | NA | NA | NA | NA | NA | NA | 0.9614 | 0.9499 | 0.0419 | 0.0602 |
| INT\_log\_Ratio\_High/Low | LABO | 0.9486 | 0.0312 | -0.5595 | -0.0565 | NA | NA | NA | -0.1145 | -0.0385 | 0.0139 | NA | NA | NA | NA | NA | NA | NA | NA | NA | NA | NA | NA | NA | NA | NA | NA | NA | NA | NA | NA | NA | NA | NA | NA | NA | NA | NA | NA | NA | NA | NA | NA | NA | NA | NA | NA | NA | 0.9614 | 0.9501 | 0.0419 | 0.0600 |
| sq\_Ratio\_High/Low | LABO | 1.0786 | 0.0345 | -0.5673 | -0.1011 | NA | NA | NA | -0.5268 | NA | NA | 0.7375 | NA | NA | NA | NA | NA | NA | NA | NA | NA | NA | NA | NA | NA | NA | NA | NA | NA | NA | NA | NA | NA | NA | NA | NA | NA | NA | NA | NA | NA | NA | NA | NA | NA | NA | NA | NA | 0.9612 | 0.9498 | 0.0423 | 0.0602 |
| INT\_sq\_Ratio\_High/Low | LABO | 1.0879 | 0.0313 | -0.5604 | -0.1729 | NA | NA | NA | -0.5464 | NA | 0.0149 | 0.5084 | NA | NA | NA | NA | NA | NA | NA | NA | NA | NA | NA | NA | NA | NA | NA | NA | NA | NA | NA | NA | NA | NA | NA | NA | NA | NA | NA | NA | NA | NA | NA | NA | NA | NA | NA | NA | 0.9612 | 0.9501 | 0.0421 | 0.0600 |
| Ratio\_SPECIES | LABO | 1.0377 | 0.0346 | -0.5758 | -0.2293 | NA | NA | NA | NA | NA | NA | NA | 0.0012 | NA | 0.0012 | 0.0012 | 0.0010 | 0.0005 | 0.0012 | 0.0008 | 0.0000 | NA | NA | NA | NA | NA | NA | NA | NA | NA | NA | NA | NA | NA | NA | NA | NA | NA | NA | NA | NA | NA | NA | NA | NA | NA | NA | NA | 0.9593 | 0.9482 | 0.0422 | 0.0611 |
| log\_Ratio\_SPECIES | LABO | 0.9907 | 0.0348 | -0.5736 | 0.1489 | NA | NA | NA | NA | NA | NA | NA | -0.0008 | NA | -0.0008 | -0.0008 | 0.0000 | 0.0026 | -0.0008 | 0.0010 | 0.0053 | 0.0004 | NA | 0.0004 | 0.0004 | -0.0036 | -0.0160 | 0.0004 | -0.0085 | -0.0295 | NA | NA | NA | NA | NA | NA | NA | NA | NA | NA | NA | NA | NA | NA | NA | NA | NA | NA | 0.9623 | 0.9508 | 0.0423 | 0.0598 |
| INT\_log\_Ratio\_SPECIES | LABO | 0.9919 | 0.0347 | -0.5735 | 0.1517 | NA | NA | NA | NA | NA | NA | NA | -0.0010 | NA | -0.0010 | -0.0010 | -0.0002 | 0.0024 | -0.0010 | 0.0009 | 0.0051 | 0.0005 | NA | 0.0005 | 0.0005 | -0.0035 | -0.0160 | 0.0005 | -0.0085 | -0.0294 | 0e+00 | NA | 0.0000 | 0e+00 | 0e+00 | 0.0000 | 0e+00 | 0.0000 | 0e+00 | NA | NA | NA | NA | NA | NA | NA | NA | NA | 0.9623 | 0.9508 | 0.0424 | 0.0599 |
| sq\_Ratio\_SPECIES | LABO | 1.0376 | 0.0346 | -0.5757 | -0.2338 | NA | NA | NA | NA | NA | NA | NA | 0.0026 | NA | 0.0026 | 0.0026 | 0.0017 | -0.0011 | 0.0026 | 0.0006 | -0.0041 | NA | NA | NA | NA | NA | NA | NA | NA | NA | NA | NA | NA | NA | NA | NA | NA | NA | NA | -1e-04 | NA | -1e-04 | -1e-04 | 0e+00 | 1e-04 | -1e-04 | 0e+00 | 2e-04 | 0.9594 | 0.9483 | 0.0423 | 0.0611 |
| INT\_sq\_Ratio\_SPECIES | LABO | 1.0467 | 0.0332 | -0.5715 | -0.2657 | NA | NA | NA | NA | NA | NA | NA | 0.0034 | NA | 0.0034 | 0.0034 | 0.0014 | -0.0046 | 0.0034 | -0.0009 | -0.0112 | NA | NA | NA | NA | NA | NA | NA | NA | NA | 0e+00 | NA | 0.0000 | 0e+00 | 1e-04 | 0.0003 | 0e+00 | 0.0001 | 6e-04 | -1e-04 | NA | -1e-04 | -1e-04 | -1e-04 | 0e+00 | -1e-04 | -1e-04 | 0e+00 | 0.9598 | 0.9487 | 0.0426 | 0.0610 |
| Ratio\_Bats | LACI | 0.9259 | 0.0383 | -0.5651 | -0.0836 | NA | NA | NA | NA | NA | NA | NA | NA | NA | NA | NA | NA | NA | NA | NA | NA | NA | NA | NA | NA | NA | NA | NA | NA | NA | NA | NA | NA | NA | NA | NA | NA | NA | NA | NA | NA | NA | NA | NA | NA | NA | NA | NA | 0.9538 | 0.9436 | 0.0424 | 0.0570 |
| log\_Ratio\_Bats | LACI | 0.6502 | 0.0368 | -0.5384 | 0.3192 | -0.0833 | NA | NA | NA | NA | NA | NA | NA | NA | NA | NA | NA | NA | NA | NA | NA | NA | NA | NA | NA | NA | NA | NA | NA | NA | NA | NA | NA | NA | NA | NA | NA | NA | NA | NA | NA | NA | NA | NA | NA | NA | NA | NA | 0.9582 | 0.9477 | 0.0414 | 0.0549 |
| INT\_log\_Ratio\_Bats | LACI | 0.6380 | 0.0372 | -0.5394 | 0.3851 | -0.0861 | -0.0031 | NA | NA | NA | NA | NA | NA | NA | NA | NA | NA | NA | NA | NA | NA | NA | NA | NA | NA | NA | NA | NA | NA | NA | NA | NA | NA | NA | NA | NA | NA | NA | NA | NA | NA | NA | NA | NA | NA | NA | NA | NA | 0.9582 | 0.9477 | 0.0414 | 0.0549 |
| sq\_Ratio\_Bats | LACI | 0.9503 | 0.0379 | -0.5539 | -0.6014 | NA | NA | 0.8612 | NA | NA | NA | NA | NA | NA | NA | NA | NA | NA | NA | NA | NA | NA | NA | NA | NA | NA | NA | NA | NA | NA | NA | NA | NA | NA | NA | NA | NA | NA | NA | NA | NA | NA | NA | NA | NA | NA | NA | NA | 0.9556 | 0.9450 | 0.0422 | 0.0563 |
| INT\_sq\_Ratio\_Bats | LACI | 0.9564 | 0.0360 | -0.5487 | -0.6940 | NA | 0.0127 | 0.6569 | NA | NA | NA | NA | NA | NA | NA | NA | NA | NA | NA | NA | NA | NA | NA | NA | NA | NA | NA | NA | NA | NA | NA | NA | NA | NA | NA | NA | NA | NA | NA | NA | NA | NA | NA | NA | NA | NA | NA | NA | 0.9558 | 0.9452 | 0.0421 | 0.0562 |
| Ratio\_High/Low | LACI | 0.9748 | 0.0386 | -0.5655 | 0.2688 | NA | NA | NA | -0.2728 | NA | NA | NA | NA | NA | NA | NA | NA | NA | NA | NA | NA | NA | NA | NA | NA | NA | NA | NA | NA | NA | NA | NA | NA | NA | NA | NA | NA | NA | NA | NA | NA | NA | NA | NA | NA | NA | NA | NA | 0.9584 | 0.9470 | 0.0425 | 0.0553 |
| log\_Ratio\_High/Low | LACI | 0.6767 | 0.0371 | -0.5452 | -0.0755 | NA | NA | NA | 0.2643 | -0.1136 | NA | NA | NA | NA | NA | NA | NA | NA | NA | NA | NA | NA | NA | NA | NA | NA | NA | NA | NA | NA | NA | NA | NA | NA | NA | NA | NA | NA | NA | NA | NA | NA | NA | NA | NA | NA | NA | NA | 0.9618 | 0.9498 | 0.0412 | 0.0538 |
| INT\_log\_Ratio\_High/Low | LACI | 0.6968 | 0.0364 | -0.5445 | -0.0826 | NA | NA | NA | 0.2239 | -0.1076 | 0.0020 | NA | NA | NA | NA | NA | NA | NA | NA | NA | NA | NA | NA | NA | NA | NA | NA | NA | NA | NA | NA | NA | NA | NA | NA | NA | NA | NA | NA | NA | NA | NA | NA | NA | NA | NA | NA | NA | 0.9618 | 0.9498 | 0.0413 | 0.0538 |
| sq\_Ratio\_High/Low | LACI | 1.0530 | 0.0373 | -0.5414 | -0.4329 | NA | NA | NA | -0.7454 | NA | NA | 0.9995 | NA | NA | NA | NA | NA | NA | NA | NA | NA | NA | NA | NA | NA | NA | NA | NA | NA | NA | NA | NA | NA | NA | NA | NA | NA | NA | NA | NA | NA | NA | NA | NA | NA | NA | NA | NA | 0.9615 | 0.9500 | 0.0418 | 0.0537 |
| INT\_sq\_Ratio\_High/Low | LACI | 1.0530 | 0.0374 | -0.5414 | -0.4344 | NA | NA | NA | -0.7470 | NA | -0.0002 | 1.0061 | NA | NA | NA | NA | NA | NA | NA | NA | NA | NA | NA | NA | NA | NA | NA | NA | NA | NA | NA | NA | NA | NA | NA | NA | NA | NA | NA | NA | NA | NA | NA | NA | NA | NA | NA | NA | 0.9615 | 0.9500 | 0.0418 | 0.0537 |
| Ratio\_SPECIES | LACI | 0.9280 | 0.0380 | -0.5609 | -0.2377 | NA | NA | NA | NA | NA | NA | NA | 0.0003 | 0.0012 | NA | 0.0000 | 0.0012 | 0.0012 | 0.0012 | 0.0012 | 0.0012 | NA | NA | NA | NA | NA | NA | NA | NA | NA | NA | NA | NA | NA | NA | NA | NA | NA | NA | NA | NA | NA | NA | NA | NA | NA | NA | NA | 0.9542 | 0.9439 | 0.0424 | 0.0568 |
| log\_Ratio\_SPECIES | LACI | 0.8744 | 0.0382 | -0.5584 | 0.1927 | NA | NA | NA | NA | NA | NA | NA | 0.0049 | -0.0009 | NA | 0.0065 | -0.0009 | -0.0009 | -0.0009 | -0.0009 | -0.0009 | -0.0274 | -0.0002 | NA | -0.0352 | -0.0002 | -0.0002 | -0.0002 | -0.0002 | -0.0002 | NA | NA | NA | NA | NA | NA | NA | NA | NA | NA | NA | NA | NA | NA | NA | NA | NA | NA | 0.9605 | 0.9487 | 0.0422 | 0.0546 |
| INT\_log\_Ratio\_SPECIES | LACI | 0.8579 | 0.0393 | -0.5596 | 0.1562 | NA | NA | NA | NA | NA | NA | NA | 0.0084 | 0.0011 | NA | 0.0099 | 0.0011 | 0.0011 | 0.0011 | 0.0011 | 0.0011 | -0.0288 | -0.0009 | NA | -0.0365 | -0.0009 | -0.0009 | -0.0009 | -0.0009 | -0.0009 | -2e-04 | -1e-04 | NA | -2e-04 | -1e-04 | -0.0001 | -1e-04 | -0.0001 | -1e-04 | NA | NA | NA | NA | NA | NA | NA | NA | NA | 0.9607 | 0.9490 | 0.0421 | 0.0545 |
| sq\_Ratio\_SPECIES | LACI | 0.9254 | 0.0379 | -0.5587 | -0.3478 | NA | NA | NA | NA | NA | NA | NA | -0.0015 | 0.0039 | NA | -0.0032 | 0.0039 | 0.0039 | 0.0039 | 0.0039 | 0.0039 | NA | NA | NA | NA | NA | NA | NA | NA | NA | NA | NA | NA | NA | NA | NA | NA | NA | NA | 1e-04 | -1e-04 | NA | 2e-04 | -1e-04 | -1e-04 | -1e-04 | -1e-04 | -1e-04 | 0.9545 | 0.9442 | 0.0424 | 0.0568 |
| INT\_sq\_Ratio\_SPECIES | LACI | 0.9277 | 0.0375 | -0.5576 | -0.3560 | NA | NA | NA | NA | NA | NA | NA | -0.0065 | 0.0055 | NA | -0.0103 | 0.0055 | 0.0055 | 0.0055 | 0.0055 | 0.0055 | NA | NA | NA | NA | NA | NA | NA | NA | NA | 4e-04 | -1e-04 | NA | 6e-04 | -1e-04 | -0.0001 | -1e-04 | -0.0001 | -1e-04 | 0e+00 | -1e-04 | NA | 0e+00 | -1e-04 | -1e-04 | -1e-04 | -1e-04 | -1e-04 | 0.9549 | 0.9444 | 0.0425 | 0.0568 |
| Ratio\_Bats | LANO | 0.9844 | 0.0351 | -0.5599 | -0.1659 | NA | NA | NA | NA | NA | NA | NA | NA | NA | NA | NA | NA | NA | NA | NA | NA | NA | NA | NA | NA | NA | NA | NA | NA | NA | NA | NA | NA | NA | NA | NA | NA | NA | NA | NA | NA | NA | NA | NA | NA | NA | NA | NA | 0.9465 | 0.9412 | 0.0402 | 0.0617 |
| log\_Ratio\_Bats | LANO | 0.5522 | 0.0327 | -0.5180 | 0.4655 | -0.1306 | NA | NA | NA | NA | NA | NA | NA | NA | NA | NA | NA | NA | NA | NA | NA | NA | NA | NA | NA | NA | NA | NA | NA | NA | NA | NA | NA | NA | NA | NA | NA | NA | NA | NA | NA | NA | NA | NA | NA | NA | NA | NA | 0.9590 | 0.9499 | 0.0376 | 0.0570 |
| INT\_log\_Ratio\_Bats | LANO | 0.6030 | 0.0309 | -0.5139 | 0.1913 | -0.1188 | 0.0128 | NA | NA | NA | NA | NA | NA | NA | NA | NA | NA | NA | NA | NA | NA | NA | NA | NA | NA | NA | NA | NA | NA | NA | NA | NA | NA | NA | NA | NA | NA | NA | NA | NA | NA | NA | NA | NA | NA | NA | NA | NA | 0.9590 | 0.9501 | 0.0376 | 0.0569 |
| sq\_Ratio\_Bats | LANO | 1.0297 | 0.0343 | -0.5391 | -1.1297 | NA | NA | 1.6029 | NA | NA | NA | NA | NA | NA | NA | NA | NA | NA | NA | NA | NA | NA | NA | NA | NA | NA | NA | NA | NA | NA | NA | NA | NA | NA | NA | NA | NA | NA | NA | NA | NA | NA | NA | NA | NA | NA | NA | NA | 0.9527 | 0.9454 | 0.0396 | 0.0595 |
| INT\_sq\_Ratio\_Bats | LANO | 1.0448 | 0.0296 | -0.5264 | -1.3567 | NA | 0.0310 | 1.1020 | NA | NA | NA | NA | NA | NA | NA | NA | NA | NA | NA | NA | NA | NA | NA | NA | NA | NA | NA | NA | NA | NA | NA | NA | NA | NA | NA | NA | NA | NA | NA | NA | NA | NA | NA | NA | NA | NA | NA | NA | 0.9536 | 0.9464 | 0.0391 | 0.0590 |
| Ratio\_High/Low | LANO | 1.0751 | 0.0357 | -0.5607 | 0.4881 | NA | NA | NA | -0.5063 | NA | NA | NA | NA | NA | NA | NA | NA | NA | NA | NA | NA | NA | NA | NA | NA | NA | NA | NA | NA | NA | NA | NA | NA | NA | NA | NA | NA | NA | NA | NA | NA | NA | NA | NA | NA | NA | NA | NA | 0.9614 | 0.9513 | 0.0396 | 0.0561 |
| log\_Ratio\_High/Low | LANO | 0.7212 | 0.0339 | -0.5365 | 0.0794 | NA | NA | NA | 0.1313 | -0.1349 | NA | NA | NA | NA | NA | NA | NA | NA | NA | NA | NA | NA | NA | NA | NA | NA | NA | NA | NA | NA | NA | NA | NA | NA | NA | NA | NA | NA | NA | NA | NA | NA | NA | NA | NA | NA | NA | NA | 0.9669 | 0.9548 | 0.0370 | 0.0542 |
| INT\_log\_Ratio\_High/Low | LANO | 0.9405 | 0.0258 | -0.5297 | 0.0024 | NA | NA | NA | -0.3093 | -0.0699 | 0.0216 | NA | NA | NA | NA | NA | NA | NA | NA | NA | NA | NA | NA | NA | NA | NA | NA | NA | NA | NA | NA | NA | NA | NA | NA | NA | NA | NA | NA | NA | NA | NA | NA | NA | NA | NA | NA | NA | 0.9677 | 0.9561 | 0.0372 | 0.0534 |
| sq\_Ratio\_High/Low | LANO | 1.2023 | 0.0335 | -0.5215 | -0.6526 | NA | NA | NA | -1.2746 | NA | NA | 1.6249 | NA | NA | NA | NA | NA | NA | NA | NA | NA | NA | NA | NA | NA | NA | NA | NA | NA | NA | NA | NA | NA | NA | NA | NA | NA | NA | NA | NA | NA | NA | NA | NA | NA | NA | NA | NA | 0.9692 | 0.9584 | 0.0368 | 0.0519 |
| INT\_sq\_Ratio\_High/Low | LANO | 1.2035 | 0.0306 | -0.5198 | -0.6051 | NA | NA | NA | -1.2244 | NA | 0.0077 | 1.4178 | NA | NA | NA | NA | NA | NA | NA | NA | NA | NA | NA | NA | NA | NA | NA | NA | NA | NA | NA | NA | NA | NA | NA | NA | NA | NA | NA | NA | NA | NA | NA | NA | NA | NA | NA | NA | 0.9694 | 0.9585 | 0.0367 | 0.0519 |
| Ratio\_SPECIES | LANO | 0.9883 | 0.0346 | -0.5519 | -0.4589 | NA | NA | NA | NA | NA | NA | NA | 0.0006 | 0.0020 | 0.0001 | NA | 0.0023 | 0.0023 | 0.0023 | 0.0023 | 0.0023 | NA | NA | NA | NA | NA | NA | NA | NA | NA | NA | NA | NA | NA | NA | NA | NA | NA | NA | NA | NA | NA | NA | NA | NA | NA | NA | NA | 0.9480 | 0.9421 | 0.0403 | 0.0612 |
| log\_Ratio\_SPECIES | LANO | 0.8964 | 0.0348 | -0.5476 | 0.2800 | NA | NA | NA | NA | NA | NA | NA | 0.0083 | 0.0004 | 0.0109 | NA | -0.0017 | -0.0017 | -0.0017 | -0.0017 | -0.0017 | -0.0469 | -0.0089 | -0.0595 | NA | 0.0012 | 0.0012 | 0.0012 | 0.0012 | 0.0012 | NA | NA | NA | NA | NA | NA | NA | NA | NA | NA | NA | NA | NA | NA | NA | NA | NA | NA | 0.9658 | 0.9545 | 0.0383 | 0.0546 |
| INT\_log\_Ratio\_SPECIES | LANO | 0.8920 | 0.0351 | -0.5479 | 0.2703 | NA | NA | NA | NA | NA | NA | NA | 0.0094 | 0.0013 | 0.0119 | NA | -0.0013 | -0.0013 | -0.0013 | -0.0013 | -0.0013 | -0.0474 | -0.0092 | -0.0599 | NA | 0.0011 | 0.0011 | 0.0011 | 0.0011 | 0.0011 | 0e+00 | 0e+00 | 0.0000 | NA | 0e+00 | 0.0000 | 0e+00 | 0.0000 | 0e+00 | NA | NA | NA | NA | NA | NA | NA | NA | NA | 0.9659 | 0.9545 | 0.0383 | 0.0546 |
| sq\_Ratio\_SPECIES | LANO | 0.9869 | 0.0345 | -0.5507 | -0.5178 | NA | NA | NA | NA | NA | NA | NA | -0.0046 | 0.0037 | -0.0075 | NA | 0.0057 | 0.0057 | 0.0057 | 0.0057 | 0.0057 | NA | NA | NA | NA | NA | NA | NA | NA | NA | NA | NA | NA | NA | NA | NA | NA | NA | NA | 3e-04 | -1e-04 | 5e-04 | NA | -2e-04 | -2e-04 | -2e-04 | -2e-04 | -2e-04 | 0.9487 | 0.9426 | 0.0404 | 0.0610 |
| INT\_sq\_Ratio\_SPECIES | LANO | 1.0018 | 0.0321 | -0.5438 | -0.5700 | NA | NA | NA | NA | NA | NA | NA | -0.0151 | 0.0031 | -0.0213 | NA | 0.0076 | 0.0076 | 0.0076 | 0.0076 | 0.0076 | NA | NA | NA | NA | NA | NA | NA | NA | NA | 9e-04 | 1e-04 | 0.0011 | NA | -1e-04 | -0.0001 | -1e-04 | -0.0001 | -1e-04 | 0e+00 | -1e-04 | 0e+00 | NA | -2e-04 | -2e-04 | -2e-04 | -2e-04 | -2e-04 | 0.9506 | 0.9440 | 0.0405 | 0.0605 |
| Ratio\_Bats | MYLE | 1.0263 | 0.0365 | -0.5863 | -0.1129 | NA | NA | NA | NA | NA | NA | NA | NA | NA | NA | NA | NA | NA | NA | NA | NA | NA | NA | NA | NA | NA | NA | NA | NA | NA | NA | NA | NA | NA | NA | NA | NA | NA | NA | NA | NA | NA | NA | NA | NA | NA | NA | NA | 0.9523 | 0.9410 | 0.0446 | 0.0647 |
| log\_Ratio\_Bats | MYLE | 0.7513 | 0.0350 | -0.5596 | 0.2888 | -0.0831 | NA | NA | NA | NA | NA | NA | NA | NA | NA | NA | NA | NA | NA | NA | NA | NA | NA | NA | NA | NA | NA | NA | NA | NA | NA | NA | NA | NA | NA | NA | NA | NA | NA | NA | NA | NA | NA | NA | NA | NA | NA | NA | 0.9565 | 0.9442 | 0.0439 | 0.0630 |
| INT\_log\_Ratio\_Bats | MYLE | 0.8040 | 0.0331 | -0.5553 | 0.0045 | -0.0709 | 0.0133 | NA | NA | NA | NA | NA | NA | NA | NA | NA | NA | NA | NA | NA | NA | NA | NA | NA | NA | NA | NA | NA | NA | NA | NA | NA | NA | NA | NA | NA | NA | NA | NA | NA | NA | NA | NA | NA | NA | NA | NA | NA | 0.9565 | 0.9444 | 0.0440 | 0.0629 |
| sq\_Ratio\_Bats | MYLE | 1.0570 | 0.0359 | -0.5722 | -0.7662 | NA | NA | 1.0865 | NA | NA | NA | NA | NA | NA | NA | NA | NA | NA | NA | NA | NA | NA | NA | NA | NA | NA | NA | NA | NA | NA | NA | NA | NA | NA | NA | NA | NA | NA | NA | NA | NA | NA | NA | NA | NA | NA | NA | NA | 0.9546 | 0.9427 | 0.0447 | 0.0638 |
| INT\_sq\_Ratio\_Bats | MYLE | 1.0683 | 0.0324 | -0.5627 | -0.9357 | NA | 0.0232 | 0.7125 | NA | NA | NA | NA | NA | NA | NA | NA | NA | NA | NA | NA | NA | NA | NA | NA | NA | NA | NA | NA | NA | NA | NA | NA | NA | NA | NA | NA | NA | NA | NA | NA | NA | NA | NA | NA | NA | NA | NA | NA | 0.9549 | 0.9432 | 0.0445 | 0.0635 |
| Ratio\_High/Low | MYLE | 1.0491 | 0.0367 | -0.5870 | 0.6346 | NA | NA | NA | -0.6424 | NA | NA | NA | NA | NA | NA | NA | NA | NA | NA | NA | NA | NA | NA | NA | NA | NA | NA | NA | NA | NA | NA | NA | NA | NA | NA | NA | NA | NA | NA | NA | NA | NA | NA | NA | NA | NA | NA | NA | 0.9542 | 0.9427 | 0.0446 | 0.0638 |
| log\_Ratio\_High/Low | MYLE | 0.7770 | 0.0352 | -0.5630 | -0.0280 | NA | NA | NA | 0.2484 | -0.0872 | NA | NA | NA | NA | NA | NA | NA | NA | NA | NA | NA | NA | NA | NA | NA | NA | NA | NA | NA | NA | NA | NA | NA | NA | NA | NA | NA | NA | NA | NA | NA | NA | NA | NA | NA | NA | NA | NA | 0.9573 | 0.9450 | 0.0439 | 0.0626 |
| INT\_log\_Ratio\_High/Low | MYLE | 0.8536 | 0.0320 | -0.5570 | -0.1386 | NA | NA | NA | 0.0194 | -0.0683 | 0.0152 | NA | NA | NA | NA | NA | NA | NA | NA | NA | NA | NA | NA | NA | NA | NA | NA | NA | NA | NA | NA | NA | NA | NA | NA | NA | NA | NA | NA | NA | NA | NA | NA | NA | NA | NA | NA | NA | 0.9574 | 0.9453 | 0.0440 | 0.0624 |
| sq\_Ratio\_High/Low | MYLE | 1.0859 | 0.0359 | -0.5685 | -0.1759 | NA | NA | NA | -0.7161 | NA | NA | 1.0497 | NA | NA | NA | NA | NA | NA | NA | NA | NA | NA | NA | NA | NA | NA | NA | NA | NA | NA | NA | NA | NA | NA | NA | NA | NA | NA | NA | NA | NA | NA | NA | NA | NA | NA | NA | NA | 0.9565 | 0.9445 | 0.0447 | 0.0629 |
| INT\_sq\_Ratio\_High/Low | MYLE | 1.0978 | 0.0318 | -0.5598 | -0.2667 | NA | NA | NA | -0.7409 | NA | 0.0189 | 0.7601 | NA | NA | NA | NA | NA | NA | NA | NA | NA | NA | NA | NA | NA | NA | NA | NA | NA | NA | NA | NA | NA | NA | NA | NA | NA | NA | NA | NA | NA | NA | NA | NA | NA | NA | NA | NA | 0.9567 | 0.9450 | 0.0444 | 0.0626 |
| Ratio\_SPECIES | MYLE | 1.0289 | 0.0361 | -0.5808 | -0.3139 | NA | NA | NA | NA | NA | NA | NA | 0.0016 | 0.0015 | 0.0016 | 0.0016 | NA | 0.0011 | 0.0008 | 0.0001 | 0.0015 | NA | NA | NA | NA | NA | NA | NA | NA | NA | NA | NA | NA | NA | NA | NA | NA | NA | NA | NA | NA | NA | NA | NA | NA | NA | NA | NA | 0.9528 | 0.9414 | 0.0447 | 0.0645 |
| log\_Ratio\_SPECIES | MYLE | 0.9646 | 0.0363 | -0.5778 | 0.2031 | NA | NA | NA | NA | NA | NA | NA | -0.0013 | -0.0005 | -0.0013 | -0.0013 | NA | 0.0016 | 0.0036 | 0.0073 | -0.0005 | 0.0010 | -0.0026 | 0.0010 | 0.0010 | NA | -0.0123 | -0.0219 | -0.0401 | -0.0026 | NA | NA | NA | NA | NA | NA | NA | NA | NA | NA | NA | NA | NA | NA | NA | NA | NA | NA | 0.9587 | 0.9462 | 0.0446 | 0.0621 |
| INT\_log\_Ratio\_SPECIES | MYLE | 0.9633 | 0.0364 | -0.5779 | 0.2002 | NA | NA | NA | NA | NA | NA | NA | -0.0014 | -0.0003 | -0.0014 | -0.0014 | NA | 0.0020 | 0.0042 | 0.0079 | -0.0003 | 0.0011 | -0.0026 | 0.0011 | 0.0011 | NA | -0.0125 | -0.0221 | -0.0403 | -0.0026 | 0e+00 | 0e+00 | 0.0000 | 0e+00 | NA | 0.0000 | 0e+00 | 0.0000 | 0e+00 | NA | NA | NA | NA | NA | NA | NA | NA | NA | 0.9587 | 0.9462 | 0.0446 | 0.0621 |
| sq\_Ratio\_SPECIES | MYLE | 1.0282 | 0.0361 | -0.5802 | -0.3447 | NA | NA | NA | NA | NA | NA | NA | 0.0038 | 0.0032 | 0.0038 | 0.0038 | NA | 0.0011 | -0.0009 | -0.0050 | 0.0032 | NA | NA | NA | NA | NA | NA | NA | NA | NA | NA | NA | NA | NA | NA | NA | NA | NA | NA | -1e-04 | -1e-04 | -1e-04 | -1e-04 | NA | 0e+00 | 1e-04 | 3e-04 | -1e-04 | 0.9530 | 0.9415 | 0.0448 | 0.0645 |
| INT\_sq\_Ratio\_SPECIES | MYLE | 1.0394 | 0.0343 | -0.5750 | -0.3840 | NA | NA | NA | NA | NA | NA | NA | 0.0050 | 0.0036 | 0.0050 | 0.0050 | NA | -0.0010 | -0.0054 | -0.0143 | 0.0036 | NA | NA | NA | NA | NA | NA | NA | NA | NA | -1e-04 | 0e+00 | -0.0001 | -1e-04 | NA | 0.0002 | 4e-04 | 0.0008 | 0e+00 | -1e-04 | -1e-04 | -1e-04 | -1e-04 | NA | -1e-04 | 0e+00 | 0e+00 | -1e-04 | 0.9537 | 0.9421 | 0.0450 | 0.0643 |
| Ratio\_Bats | MYLU | 1.0693 | 0.0323 | -0.5688 | -0.1912 | NA | NA | NA | NA | NA | NA | NA | NA | NA | NA | NA | NA | NA | NA | NA | NA | NA | NA | NA | NA | NA | NA | NA | NA | NA | NA | NA | NA | NA | NA | NA | NA | NA | NA | NA | NA | NA | NA | NA | NA | NA | NA | NA | 0.9456 | 0.9318 | 0.0451 | 0.0728 |
| log\_Ratio\_Bats | MYLU | 0.6758 | 0.0301 | -0.5307 | 0.3837 | -0.1189 | NA | NA | NA | NA | NA | NA | NA | NA | NA | NA | NA | NA | NA | NA | NA | NA | NA | NA | NA | NA | NA | NA | NA | NA | NA | NA | NA | NA | NA | NA | NA | NA | NA | NA | NA | NA | NA | NA | NA | NA | NA | NA | 0.9543 | 0.9378 | 0.0436 | 0.0696 |
| INT\_log\_Ratio\_Bats | MYLU | 0.7905 | 0.0260 | -0.5213 | -0.2351 | -0.0924 | 0.0289 | NA | NA | NA | NA | NA | NA | NA | NA | NA | NA | NA | NA | NA | NA | NA | NA | NA | NA | NA | NA | NA | NA | NA | NA | NA | NA | NA | NA | NA | NA | NA | NA | NA | NA | NA | NA | NA | NA | NA | NA | NA | 0.9544 | 0.9386 | 0.0436 | 0.0692 |
| sq\_Ratio\_Bats | MYLU | 1.1184 | 0.0314 | -0.5464 | -1.2354 | NA | NA | 1.7366 | NA | NA | NA | NA | NA | NA | NA | NA | NA | NA | NA | NA | NA | NA | NA | NA | NA | NA | NA | NA | NA | NA | NA | NA | NA | NA | NA | NA | NA | NA | NA | NA | NA | NA | NA | NA | NA | NA | NA | NA | 0.9514 | 0.9359 | 0.0450 | 0.0706 |
| INT\_sq\_Ratio\_Bats | MYLU | 1.1372 | 0.0256 | -0.5306 | -1.5166 | NA | 0.0385 | 1.1163 | NA | NA | NA | NA | NA | NA | NA | NA | NA | NA | NA | NA | NA | NA | NA | NA | NA | NA | NA | NA | NA | NA | NA | NA | NA | NA | NA | NA | NA | NA | NA | NA | NA | NA | NA | NA | NA | NA | NA | NA | 0.9518 | 0.9372 | 0.0443 | 0.0700 |
| Ratio\_High/Low | MYLU | 1.1048 | 0.0326 | -0.5700 | 0.9691 | NA | NA | NA | -0.9972 | NA | NA | NA | NA | NA | NA | NA | NA | NA | NA | NA | NA | NA | NA | NA | NA | NA | NA | NA | NA | NA | NA | NA | NA | NA | NA | NA | NA | NA | NA | NA | NA | NA | NA | NA | NA | NA | NA | NA | 0.9502 | 0.9357 | 0.0449 | 0.0707 |
| log\_Ratio\_High/Low | MYLU | 0.7496 | 0.0306 | -0.5386 | 0.1040 | NA | NA | NA | 0.1658 | -0.1138 | NA | NA | NA | NA | NA | NA | NA | NA | NA | NA | NA | NA | NA | NA | NA | NA | NA | NA | NA | NA | NA | NA | NA | NA | NA | NA | NA | NA | NA | NA | NA | NA | NA | NA | NA | NA | NA | NA | 0.9557 | 0.9392 | 0.0434 | 0.0689 |
| INT\_log\_Ratio\_High/Low | MYLU | 0.9014 | 0.0244 | -0.5267 | -0.1153 | NA | NA | NA | -0.2883 | -0.0764 | 0.0302 | NA | NA | NA | NA | NA | NA | NA | NA | NA | NA | NA | NA | NA | NA | NA | NA | NA | NA | NA | NA | NA | NA | NA | NA | NA | NA | NA | NA | NA | NA | NA | NA | NA | NA | NA | NA | NA | 0.9561 | 0.9403 | 0.0433 | 0.0683 |
| sq\_Ratio\_High/Low | MYLU | 1.1623 | 0.0313 | -0.5410 | -0.2964 | NA | NA | NA | -1.1122 | NA | NA | 1.6390 | NA | NA | NA | NA | NA | NA | NA | NA | NA | NA | NA | NA | NA | NA | NA | NA | NA | NA | NA | NA | NA | NA | NA | NA | NA | NA | NA | NA | NA | NA | NA | NA | NA | NA | NA | NA | 0.9555 | 0.9395 | 0.0447 | 0.0686 |
| INT\_sq\_Ratio\_High/Low | MYLU | 1.1811 | 0.0248 | -0.5272 | -0.4407 | NA | NA | NA | -1.1516 | NA | 0.0300 | 1.1786 | NA | NA | NA | NA | NA | NA | NA | NA | NA | NA | NA | NA | NA | NA | NA | NA | NA | NA | NA | NA | NA | NA | NA | NA | NA | NA | NA | NA | NA | NA | NA | NA | NA | NA | NA | NA | 0.9557 | 0.9407 | 0.0438 | 0.0680 |
| Ratio\_SPECIES | MYLU | 1.0736 | 0.0316 | -0.5600 | -0.5158 | NA | NA | NA | NA | NA | NA | NA | 0.0026 | 0.0025 | 0.0026 | 0.0026 | 0.0018 | NA | 0.0015 | -0.0002 | 0.0025 | NA | NA | NA | NA | NA | NA | NA | NA | NA | NA | NA | NA | NA | NA | NA | NA | NA | NA | NA | NA | NA | NA | NA | NA | NA | NA | NA | 0.9471 | 0.9328 | 0.0456 | 0.0723 |
| log\_Ratio\_SPECIES | MYLU | 0.9803 | 0.0319 | -0.5556 | 0.2344 | NA | NA | NA | NA | NA | NA | NA | -0.0016 | -0.0009 | -0.0016 | -0.0016 | 0.0029 | NA | 0.0042 | 0.0124 | -0.0009 | 0.0020 | -0.0017 | 0.0020 | 0.0020 | -0.0202 | NA | -0.0263 | -0.0672 | -0.0017 | NA | NA | NA | NA | NA | NA | NA | NA | NA | NA | NA | NA | NA | NA | NA | NA | NA | NA | 0.9603 | 0.9430 | 0.0439 | 0.0670 |
| INT\_log\_Ratio\_SPECIES | MYLU | 0.9914 | 0.0312 | -0.5548 | 0.2590 | NA | NA | NA | NA | NA | NA | NA | -0.0031 | -0.0022 | -0.0031 | -0.0031 | 0.0016 | NA | 0.0026 | 0.0092 | -0.0022 | 0.0025 | -0.0012 | 0.0025 | 0.0025 | -0.0197 | NA | -0.0257 | -0.0659 | -0.0012 | 1e-04 | 1e-04 | 0.0001 | 1e-04 | 1e-04 | NA | 1e-04 | 0.0001 | 1e-04 | NA | NA | NA | NA | NA | NA | NA | NA | NA | 0.9602 | 0.9431 | 0.0440 | 0.0670 |
| sq\_Ratio\_SPECIES | MYLU | 1.0742 | 0.0317 | -0.5605 | -0.4915 | NA | NA | NA | NA | NA | NA | NA | 0.0054 | 0.0046 | 0.0054 | 0.0054 | 0.0004 | NA | -0.0010 | -0.0112 | 0.0046 | NA | NA | NA | NA | NA | NA | NA | NA | NA | NA | NA | NA | NA | NA | NA | NA | NA | NA | -2e-04 | -1e-04 | -2e-04 | -2e-04 | 1e-04 | NA | 1e-04 | 6e-04 | -1e-04 | 0.9478 | 0.9332 | 0.0458 | 0.0721 |
| INT\_sq\_Ratio\_SPECIES | MYLU | 1.0964 | 0.0281 | -0.5502 | -0.5694 | NA | NA | NA | NA | NA | NA | NA | 0.0067 | 0.0050 | 0.0067 | 0.0067 | -0.0040 | NA | -0.0071 | -0.0283 | 0.0050 | NA | NA | NA | NA | NA | NA | NA | NA | NA | 0e+00 | 0e+00 | 0.0000 | 0e+00 | 4e-04 | NA | 5e-04 | 0.0014 | 0e+00 | -2e-04 | -2e-04 | -2e-04 | -2e-04 | -1e-04 | NA | -1e-04 | 1e-04 | -2e-04 | 0.9496 | 0.9352 | 0.0460 | 0.0713 |
| Ratio\_Bats | MYSE | 0.9749 | 0.0395 | -0.5902 | -0.0847 | NA | NA | NA | NA | NA | NA | NA | NA | NA | NA | NA | NA | NA | NA | NA | NA | NA | NA | NA | NA | NA | NA | NA | NA | NA | NA | NA | NA | NA | NA | NA | NA | NA | NA | NA | NA | NA | NA | NA | NA | NA | NA | NA | 0.9472 | 0.9367 | 0.0463 | 0.0639 |
| log\_Ratio\_Bats | MYSE | 0.7148 | 0.0381 | -0.5650 | 0.2953 | -0.0786 | NA | NA | NA | NA | NA | NA | NA | NA | NA | NA | NA | NA | NA | NA | NA | NA | NA | NA | NA | NA | NA | NA | NA | NA | NA | NA | NA | NA | NA | NA | NA | NA | NA | NA | NA | NA | NA | NA | NA | NA | NA | NA | 0.9517 | 0.9399 | 0.0461 | 0.0623 |
| INT\_log\_Ratio\_Bats | MYSE | 0.7231 | 0.0378 | -0.5644 | 0.2509 | -0.0767 | 0.0021 | NA | NA | NA | NA | NA | NA | NA | NA | NA | NA | NA | NA | NA | NA | NA | NA | NA | NA | NA | NA | NA | NA | NA | NA | NA | NA | NA | NA | NA | NA | NA | NA | NA | NA | NA | NA | NA | NA | NA | NA | NA | 0.9517 | 0.9399 | 0.0461 | 0.0623 |
| sq\_Ratio\_Bats | MYSE | 0.9996 | 0.0391 | -0.5789 | -0.6101 | NA | NA | 0.8738 | NA | NA | NA | NA | NA | NA | NA | NA | NA | NA | NA | NA | NA | NA | NA | NA | NA | NA | NA | NA | NA | NA | NA | NA | NA | NA | NA | NA | NA | NA | NA | NA | NA | NA | NA | NA | NA | NA | NA | NA | 0.9491 | 0.9379 | 0.0465 | 0.0633 |
| INT\_sq\_Ratio\_Bats | MYSE | 1.0071 | 0.0368 | -0.5726 | -0.7224 | NA | 0.0154 | 0.6260 | NA | NA | NA | NA | NA | NA | NA | NA | NA | NA | NA | NA | NA | NA | NA | NA | NA | NA | NA | NA | NA | NA | NA | NA | NA | NA | NA | NA | NA | NA | NA | NA | NA | NA | NA | NA | NA | NA | NA | NA | 0.9494 | 0.9382 | 0.0465 | 0.0632 |
| Ratio\_High/Low | MYSE | 0.9922 | 0.0397 | -0.5908 | 0.4809 | NA | NA | NA | -0.4861 | NA | NA | NA | NA | NA | NA | NA | NA | NA | NA | NA | NA | NA | NA | NA | NA | NA | NA | NA | NA | NA | NA | NA | NA | NA | NA | NA | NA | NA | NA | NA | NA | NA | NA | NA | NA | NA | NA | NA | 0.9494 | 0.9378 | 0.0465 | 0.0633 |
| log\_Ratio\_High/Low | MYSE | 0.7084 | 0.0381 | -0.5657 | -0.2104 | NA | NA | NA | 0.4433 | -0.0909 | NA | NA | NA | NA | NA | NA | NA | NA | NA | NA | NA | NA | NA | NA | NA | NA | NA | NA | NA | NA | NA | NA | NA | NA | NA | NA | NA | NA | NA | NA | NA | NA | NA | NA | NA | NA | NA | NA | 0.9530 | 0.9405 | 0.0460 | 0.0620 |
| INT\_log\_Ratio\_High/Low | MYSE | 0.7316 | 0.0371 | -0.5639 | -0.2439 | NA | NA | NA | 0.3739 | -0.0852 | 0.0046 | NA | NA | NA | NA | NA | NA | NA | NA | NA | NA | NA | NA | NA | NA | NA | NA | NA | NA | NA | NA | NA | NA | NA | NA | NA | NA | NA | NA | NA | NA | NA | NA | NA | NA | NA | NA | NA | 0.9531 | 0.9405 | 0.0460 | 0.0620 |
| sq\_Ratio\_High/Low | MYSE | 1.0234 | 0.0390 | -0.5751 | -0.2056 | NA | NA | NA | -0.5484 | NA | NA | 0.8891 | NA | NA | NA | NA | NA | NA | NA | NA | NA | NA | NA | NA | NA | NA | NA | NA | NA | NA | NA | NA | NA | NA | NA | NA | NA | NA | NA | NA | NA | NA | NA | NA | NA | NA | NA | NA | 0.9510 | 0.9392 | 0.0466 | 0.0627 |
| INT\_sq\_Ratio\_High/Low | MYSE | 1.0312 | 0.0363 | -0.5693 | -0.2652 | NA | NA | NA | -0.5647 | NA | 0.0124 | 0.6988 | NA | NA | NA | NA | NA | NA | NA | NA | NA | NA | NA | NA | NA | NA | NA | NA | NA | NA | NA | NA | NA | NA | NA | NA | NA | NA | NA | NA | NA | NA | NA | NA | NA | NA | NA | NA | 0.9513 | 0.9394 | 0.0466 | 0.0626 |
| Ratio\_SPECIES | MYSE | 0.9770 | 0.0392 | -0.5859 | -0.2431 | NA | NA | NA | NA | NA | NA | NA | 0.0012 | 0.0012 | 0.0012 | 0.0012 | 0.0006 | 0.0006 | NA | 0.0006 | 0.0011 | NA | NA | NA | NA | NA | NA | NA | NA | NA | NA | NA | NA | NA | NA | NA | NA | NA | NA | NA | NA | NA | NA | NA | NA | NA | NA | NA | 0.9476 | 0.9370 | 0.0464 | 0.0638 |
| log\_Ratio\_SPECIES | MYSE | 0.9220 | 0.0394 | -0.5833 | 0.1995 | NA | NA | NA | NA | NA | NA | NA | -0.0010 | -0.0008 | -0.0010 | -0.0010 | 0.0035 | 0.0035 | NA | 0.0035 | -0.0005 | 0.0002 | -0.0008 | 0.0002 | 0.0002 | -0.0210 | -0.0210 | NA | -0.0210 | -0.0025 | NA | NA | NA | NA | NA | NA | NA | NA | NA | NA | NA | NA | NA | NA | NA | NA | NA | NA | 0.9530 | 0.9405 | 0.0466 | 0.0622 |
| INT\_log\_Ratio\_SPECIES | MYSE | 0.9096 | 0.0403 | -0.5842 | 0.1722 | NA | NA | NA | NA | NA | NA | NA | 0.0004 | 0.0006 | 0.0004 | 0.0004 | 0.0060 | 0.0060 | NA | 0.0060 | 0.0009 | -0.0002 | -0.0013 | -0.0002 | -0.0002 | -0.0219 | -0.0219 | NA | -0.0219 | -0.0029 | -1e-04 | -1e-04 | -0.0001 | -1e-04 | -1e-04 | -0.0001 | NA | -0.0001 | -1e-04 | NA | NA | NA | NA | NA | NA | NA | NA | NA | 0.9531 | 0.9407 | 0.0466 | 0.0622 |
| sq\_Ratio\_SPECIES | MYSE | 0.9749 | 0.0391 | -0.5841 | -0.3328 | NA | NA | NA | NA | NA | NA | NA | 0.0037 | 0.0035 | 0.0037 | 0.0037 | -0.0004 | -0.0004 | NA | -0.0004 | 0.0032 | NA | NA | NA | NA | NA | NA | NA | NA | NA | NA | NA | NA | NA | NA | NA | NA | NA | NA | -1e-04 | -1e-04 | -1e-04 | -1e-04 | 1e-04 | 1e-04 | NA | 1e-04 | -1e-04 | 0.9478 | 0.9371 | 0.0464 | 0.0638 |
| INT\_sq\_Ratio\_SPECIES | MYSE | 0.9794 | 0.0384 | -0.5820 | -0.3487 | NA | NA | NA | NA | NA | NA | NA | 0.0052 | 0.0048 | 0.0052 | 0.0052 | -0.0041 | -0.0041 | NA | -0.0041 | 0.0039 | NA | NA | NA | NA | NA | NA | NA | NA | NA | -1e-04 | -1e-04 | -0.0001 | -1e-04 | 3e-04 | 0.0003 | NA | 0.0003 | 0e+00 | -1e-04 | -1e-04 | -1e-04 | -1e-04 | 0e+00 | 0e+00 | NA | 0e+00 | -1e-04 | 0.9481 | 0.9372 | 0.0466 | 0.0638 |
| Ratio\_Bats | MYSO | 1.0601 | 0.0325 | -0.5702 | -0.1264 | NA | NA | NA | NA | NA | NA | NA | NA | NA | NA | NA | NA | NA | NA | NA | NA | NA | NA | NA | NA | NA | NA | NA | NA | NA | NA | NA | NA | NA | NA | NA | NA | NA | NA | NA | NA | NA | NA | NA | NA | NA | NA | NA | 0.9580 | 0.9456 | 0.0416 | 0.0641 |
| log\_Ratio\_Bats | MYSO | 0.8079 | 0.0311 | -0.5457 | 0.2420 | -0.0762 | NA | NA | NA | NA | NA | NA | NA | NA | NA | NA | NA | NA | NA | NA | NA | NA | NA | NA | NA | NA | NA | NA | NA | NA | NA | NA | NA | NA | NA | NA | NA | NA | NA | NA | NA | NA | NA | NA | NA | NA | NA | NA | 0.9618 | 0.9482 | 0.0408 | 0.0626 |
| INT\_log\_Ratio\_Bats | MYSO | 0.8912 | 0.0281 | -0.5389 | -0.2072 | -0.0570 | 0.0210 | NA | NA | NA | NA | NA | NA | NA | NA | NA | NA | NA | NA | NA | NA | NA | NA | NA | NA | NA | NA | NA | NA | NA | NA | NA | NA | NA | NA | NA | NA | NA | NA | NA | NA | NA | NA | NA | NA | NA | NA | NA | 0.9618 | 0.9486 | 0.0408 | 0.0624 |
| sq\_Ratio\_Bats | MYSO | 1.0919 | 0.0320 | -0.5556 | -0.8019 | NA | NA | 1.1235 | NA | NA | NA | NA | NA | NA | NA | NA | NA | NA | NA | NA | NA | NA | NA | NA | NA | NA | NA | NA | NA | NA | NA | NA | NA | NA | NA | NA | NA | NA | NA | NA | NA | NA | NA | NA | NA | NA | NA | NA | 0.9605 | 0.9474 | 0.0416 | 0.0631 |
| INT\_sq\_Ratio\_Bats | MYSO | 1.1049 | 0.0279 | -0.5446 | -0.9980 | NA | 0.0268 | 0.6909 | NA | NA | NA | NA | NA | NA | NA | NA | NA | NA | NA | NA | NA | NA | NA | NA | NA | NA | NA | NA | NA | NA | NA | NA | NA | NA | NA | NA | NA | NA | NA | NA | NA | NA | NA | NA | NA | NA | NA | NA | 0.9607 | 0.9480 | 0.0412 | 0.0627 |
| Ratio\_High/Low | MYSO | 1.0834 | 0.0328 | -0.5709 | 0.6352 | NA | NA | NA | -0.6545 | NA | NA | NA | NA | NA | NA | NA | NA | NA | NA | NA | NA | NA | NA | NA | NA | NA | NA | NA | NA | NA | NA | NA | NA | NA | NA | NA | NA | NA | NA | NA | NA | NA | NA | NA | NA | NA | NA | NA | 0.9601 | 0.9473 | 0.0415 | 0.0631 |
| log\_Ratio\_High/Low | MYSO | 0.8563 | 0.0315 | -0.5509 | 0.0824 | NA | NA | NA | 0.0887 | -0.0727 | NA | NA | NA | NA | NA | NA | NA | NA | NA | NA | NA | NA | NA | NA | NA | NA | NA | NA | NA | NA | NA | NA | NA | NA | NA | NA | NA | NA | NA | NA | NA | NA | NA | NA | NA | NA | NA | NA | 0.9625 | 0.9488 | 0.0407 | 0.0623 |
| INT\_log\_Ratio\_High/Low | MYSO | 0.9662 | 0.0270 | -0.5423 | -0.0763 | NA | NA | NA | -0.2397 | -0.0457 | 0.0218 | NA | NA | NA | NA | NA | NA | NA | NA | NA | NA | NA | NA | NA | NA | NA | NA | NA | NA | NA | NA | NA | NA | NA | NA | NA | NA | NA | NA | NA | NA | NA | NA | NA | NA | NA | NA | NA | 0.9626 | 0.9494 | 0.0407 | 0.0619 |
| sq\_Ratio\_High/Low | MYSO | 1.1204 | 0.0319 | -0.5523 | -0.1807 | NA | NA | NA | -0.7287 | NA | NA | 1.0568 | NA | NA | NA | NA | NA | NA | NA | NA | NA | NA | NA | NA | NA | NA | NA | NA | NA | NA | NA | NA | NA | NA | NA | NA | NA | NA | NA | NA | NA | NA | NA | NA | NA | NA | NA | NA | 0.9623 | 0.9490 | 0.0414 | 0.0621 |
| INT\_sq\_Ratio\_High/Low | MYSO | 1.1339 | 0.0273 | -0.5423 | -0.2838 | NA | NA | NA | -0.7568 | NA | 0.0214 | 0.7279 | NA | NA | NA | NA | NA | NA | NA | NA | NA | NA | NA | NA | NA | NA | NA | NA | NA | NA | NA | NA | NA | NA | NA | NA | NA | NA | NA | NA | NA | NA | NA | NA | NA | NA | NA | NA | 0.9625 | 0.9496 | 0.0409 | 0.0618 |
| Ratio\_SPECIES | MYSO | 1.0629 | 0.0321 | -0.5645 | -0.3363 | NA | NA | NA | NA | NA | NA | NA | 0.0017 | 0.0016 | 0.0017 | 0.0017 | 0.0012 | -0.0004 | 0.0012 | NA | 0.0016 | NA | NA | NA | NA | NA | NA | NA | NA | NA | NA | NA | NA | NA | NA | NA | NA | NA | NA | NA | NA | NA | NA | NA | NA | NA | NA | NA | 0.9587 | 0.9460 | 0.0418 | 0.0639 |
| log\_Ratio\_SPECIES | MYSO | 0.9989 | 0.0323 | -0.5615 | 0.1780 | NA | NA | NA | NA | NA | NA | NA | -0.0010 | -0.0004 | -0.0010 | -0.0010 | 0.0013 | 0.0093 | 0.0013 | NA | -0.0004 | 0.0004 | -0.0028 | 0.0004 | 0.0004 | -0.0108 | -0.0502 | -0.0108 | NA | -0.0028 | NA | NA | NA | NA | NA | NA | NA | NA | NA | NA | NA | NA | NA | NA | NA | NA | NA | NA | 0.9650 | 0.9511 | 0.0410 | 0.0611 |
| INT\_log\_Ratio\_SPECIES | MYSO | 1.0070 | 0.0318 | -0.5609 | 0.1959 | NA | NA | NA | NA | NA | NA | NA | -0.0020 | -0.0014 | -0.0020 | -0.0020 | 0.0002 | 0.0070 | 0.0002 | NA | -0.0014 | 0.0007 | -0.0024 | 0.0007 | 0.0007 | -0.0104 | -0.0492 | -0.0104 | NA | -0.0024 | 0e+00 | 0e+00 | 0.0000 | 0e+00 | 0e+00 | 0.0001 | 0e+00 | NA | 0e+00 | NA | NA | NA | NA | NA | NA | NA | NA | NA | 0.9650 | 0.9512 | 0.0411 | 0.0611 |
| sq\_Ratio\_SPECIES | MYSO | 1.0635 | 0.0322 | -0.5651 | -0.3073 | NA | NA | NA | NA | NA | NA | NA | 0.0032 | 0.0025 | 0.0032 | 0.0032 | 0.0007 | -0.0088 | 0.0007 | NA | 0.0025 | NA | NA | NA | NA | NA | NA | NA | NA | NA | NA | NA | NA | NA | NA | NA | NA | NA | NA | -1e-04 | -1e-04 | -1e-04 | -1e-04 | 0e+00 | 5e-04 | 0e+00 | NA | -1e-04 | 0.9590 | 0.9463 | 0.0419 | 0.0638 |
| INT\_sq\_Ratio\_SPECIES | MYSO | 1.0788 | 0.0297 | -0.5580 | -0.3607 | NA | NA | NA | NA | NA | NA | NA | 0.0039 | 0.0024 | 0.0039 | 0.0039 | -0.0016 | -0.0217 | -0.0016 | NA | 0.0024 | NA | NA | NA | NA | NA | NA | NA | NA | NA | 0e+00 | 1e-04 | 0.0000 | 0e+00 | 2e-04 | 0.0011 | 2e-04 | NA | 1e-04 | -1e-04 | -1e-04 | -1e-04 | -1e-04 | -1e-04 | 1e-04 | -1e-04 | NA | -1e-04 | 0.9599 | 0.9472 | 0.0420 | 0.0633 |
| Ratio\_Bats | PESU | 0.9806 | 0.0394 | -0.5912 | -0.0916 | NA | NA | NA | NA | NA | NA | NA | NA | NA | NA | NA | NA | NA | NA | NA | NA | NA | NA | NA | NA | NA | NA | NA | NA | NA | NA | NA | NA | NA | NA | NA | NA | NA | NA | NA | NA | NA | NA | NA | NA | NA | NA | NA | 0.9474 | 0.9366 | 0.0464 | 0.0643 |
| log\_Ratio\_Bats | PESU | 0.7048 | 0.0379 | -0.5645 | 0.3113 | -0.0833 | NA | NA | NA | NA | NA | NA | NA | NA | NA | NA | NA | NA | NA | NA | NA | NA | NA | NA | NA | NA | NA | NA | NA | NA | NA | NA | NA | NA | NA | NA | NA | NA | NA | NA | NA | NA | NA | NA | NA | NA | NA | NA | 0.9522 | 0.9402 | 0.0461 | 0.0625 |
| INT\_log\_Ratio\_Bats | PESU | 0.7180 | 0.0374 | -0.5634 | 0.2402 | -0.0803 | 0.0033 | NA | NA | NA | NA | NA | NA | NA | NA | NA | NA | NA | NA | NA | NA | NA | NA | NA | NA | NA | NA | NA | NA | NA | NA | NA | NA | NA | NA | NA | NA | NA | NA | NA | NA | NA | NA | NA | NA | NA | NA | NA | 0.9523 | 0.9402 | 0.0462 | 0.0625 |
| sq\_Ratio\_Bats | PESU | 1.0072 | 0.0389 | -0.5790 | -0.6582 | NA | NA | 0.9422 | NA | NA | NA | NA | NA | NA | NA | NA | NA | NA | NA | NA | NA | NA | NA | NA | NA | NA | NA | NA | NA | NA | NA | NA | NA | NA | NA | NA | NA | NA | NA | NA | NA | NA | NA | NA | NA | NA | NA | NA | 0.9494 | 0.9381 | 0.0466 | 0.0636 |
| INT\_sq\_Ratio\_Bats | PESU | 1.0155 | 0.0364 | -0.5720 | -0.7825 | NA | 0.0170 | 0.6680 | NA | NA | NA | NA | NA | NA | NA | NA | NA | NA | NA | NA | NA | NA | NA | NA | NA | NA | NA | NA | NA | NA | NA | NA | NA | NA | NA | NA | NA | NA | NA | NA | NA | NA | NA | NA | NA | NA | NA | NA | 0.9498 | 0.9384 | 0.0466 | 0.0634 |
| Ratio\_High/Low | PESU | 0.9994 | 0.0396 | -0.5918 | 0.5245 | NA | NA | NA | -0.5296 | NA | NA | NA | NA | NA | NA | NA | NA | NA | NA | NA | NA | NA | NA | NA | NA | NA | NA | NA | NA | NA | NA | NA | NA | NA | NA | NA | NA | NA | NA | NA | NA | NA | NA | NA | NA | NA | NA | NA | 0.9497 | 0.9380 | 0.0466 | 0.0636 |
| log\_Ratio\_High/Low | PESU | 0.7005 | 0.0379 | -0.5654 | -0.2033 | NA | NA | NA | 0.4489 | -0.0957 | NA | NA | NA | NA | NA | NA | NA | NA | NA | NA | NA | NA | NA | NA | NA | NA | NA | NA | NA | NA | NA | NA | NA | NA | NA | NA | NA | NA | NA | NA | NA | NA | NA | NA | NA | NA | NA | NA | 0.9537 | 0.9409 | 0.0460 | 0.0622 |
| INT\_log\_Ratio\_High/Low | PESU | 0.7304 | 0.0367 | -0.5631 | -0.2463 | NA | NA | NA | 0.3597 | -0.0884 | 0.0059 | NA | NA | NA | NA | NA | NA | NA | NA | NA | NA | NA | NA | NA | NA | NA | NA | NA | NA | NA | NA | NA | NA | NA | NA | NA | NA | NA | NA | NA | NA | NA | NA | NA | NA | NA | NA | NA | 0.9537 | 0.9410 | 0.0461 | 0.0621 |
| sq\_Ratio\_High/Low | PESU | 1.0328 | 0.0388 | -0.5749 | -0.2119 | NA | NA | NA | -0.5965 | NA | NA | 0.9538 | NA | NA | NA | NA | NA | NA | NA | NA | NA | NA | NA | NA | NA | NA | NA | NA | NA | NA | NA | NA | NA | NA | NA | NA | NA | NA | NA | NA | NA | NA | NA | NA | NA | NA | NA | NA | 0.9515 | 0.9395 | 0.0467 | 0.0629 |
| INT\_sq\_Ratio\_High/Low | PESU | 1.0415 | 0.0359 | -0.5686 | -0.2782 | NA | NA | NA | -0.6146 | NA | 0.0138 | 0.7424 | NA | NA | NA | NA | NA | NA | NA | NA | NA | NA | NA | NA | NA | NA | NA | NA | NA | NA | NA | NA | NA | NA | NA | NA | NA | NA | NA | NA | NA | NA | NA | NA | NA | NA | NA | NA | 0.9519 | 0.9398 | 0.0466 | 0.0627 |
| Ratio\_SPECIES | PESU | 0.9828 | 0.0391 | -0.5865 | -0.2637 | NA | NA | NA | NA | NA | NA | NA | 0.0013 | 0.0005 | 0.0013 | 0.0013 | 0.0013 | 0.0009 | 0.0009 | 0.0009 | NA | NA | NA | NA | NA | NA | NA | NA | NA | NA | NA | NA | NA | NA | NA | NA | NA | NA | NA | NA | NA | NA | NA | NA | NA | NA | NA | NA | 0.9478 | 0.9370 | 0.0466 | 0.0641 |
| log\_Ratio\_SPECIES | PESU | 0.9243 | 0.0393 | -0.5838 | 0.2067 | NA | NA | NA | NA | NA | NA | NA | -0.0011 | 0.0047 | -0.0011 | -0.0011 | -0.0006 | 0.0019 | 0.0019 | 0.0019 | NA | 0.0005 | -0.0271 | 0.0005 | 0.0005 | -0.0021 | -0.0140 | -0.0140 | -0.0140 | NA | NA | NA | NA | NA | NA | NA | NA | NA | NA | NA | NA | NA | NA | NA | NA | NA | NA | NA | 0.9532 | 0.9407 | 0.0467 | 0.0624 |
| INT\_log\_Ratio\_SPECIES | PESU | 0.9126 | 0.0401 | -0.5846 | 0.1809 | NA | NA | NA | NA | NA | NA | NA | 0.0000 | 0.0073 | 0.0000 | 0.0000 | 0.0009 | 0.0040 | 0.0040 | 0.0040 | NA | 0.0001 | -0.0281 | 0.0001 | 0.0001 | -0.0026 | -0.0147 | -0.0147 | -0.0147 | NA | -1e-04 | -1e-04 | -0.0001 | -1e-04 | -1e-04 | -0.0001 | -1e-04 | -0.0001 | NA | NA | NA | NA | NA | NA | NA | NA | NA | NA | 0.9533 | 0.9409 | 0.0466 | 0.0624 |
| sq\_Ratio\_SPECIES | PESU | 0.9807 | 0.0390 | -0.5847 | -0.3540 | NA | NA | NA | NA | NA | NA | NA | 0.0040 | -0.0015 | 0.0040 | 0.0040 | 0.0035 | 0.0012 | 0.0012 | 0.0012 | NA | NA | NA | NA | NA | NA | NA | NA | NA | NA | NA | NA | NA | NA | NA | NA | NA | NA | NA | -1e-04 | 1e-04 | -1e-04 | -1e-04 | -1e-04 | 0e+00 | 0e+00 | 0e+00 | NA | 0.9479 | 0.9371 | 0.0466 | 0.0642 |
| INT\_sq\_Ratio\_SPECIES | PESU | 0.9863 | 0.0381 | -0.5821 | -0.3734 | NA | NA | NA | NA | NA | NA | NA | 0.0055 | -0.0067 | 0.0055 | 0.0055 | 0.0045 | -0.0008 | -0.0008 | -0.0008 | NA | NA | NA | NA | NA | NA | NA | NA | NA | NA | -1e-04 | 4e-04 | -0.0001 | -1e-04 | -1e-04 | 0.0002 | 2e-04 | 0.0002 | NA | -1e-04 | 0e+00 | -1e-04 | -1e-04 | -1e-04 | -1e-04 | -1e-04 | -1e-04 | NA | 0.9483 | 0.9373 | 0.0468 | 0.0642 |

## GLM Coefs p-values

p-values

| Vars | Species | (Intercept) | Ex\_Count | log\_Ex\_Count | Ratio\_Bats | log\_Ratio\_Bats | Ex\_Count\_Ratio\_Bats | sq\_Ratio\_Bats | Ratio\_High/Low | log\_Ratio\_High/Low | Ex\_Count\_Ratio\_High/Low | sq\_Ratio\_High/Low | Ratio\_EPFU | Ratio\_LABO | Ratio\_LACI | Ratio\_LANO | Ratio\_MYLE | Ratio\_MYLU | Ratio\_MYSE | Ratio\_MYSO | Ratio\_PESU | log\_Ratio\_EPFU | log\_Ratio\_LABO | log\_Ratio\_LACI | log\_Ratio\_LANO | log\_Ratio\_MYLE | log\_Ratio\_MYLU | log\_Ratio\_MYSE | log\_Ratio\_MYSO | log\_Ratio\_PESU | Ex\_Count\_Ratio\_EPFU | Ex\_Count\_Ratio\_LABO | Ex\_Count\_Ratio\_LACI | Ex\_Count\_Ratio\_LANO | Ex\_Count\_Ratio\_MYLE | Ex\_Count\_Ratio\_MYLU | Ex\_Count\_Ratio\_MYSE | Ex\_Count\_Ratio\_MYSO | Ex\_Count\_Ratio\_PESU | sq\_Ratio\_EPFU | sq\_Ratio\_LABO | sq\_Ratio\_LACI | sq\_Ratio\_LANO | sq\_Ratio\_MYLE | sq\_Ratio\_MYLU | sq\_Ratio\_MYSE | sq\_Ratio\_MYSO | sq\_Ratio\_PESU |
| --- | --- | --- | --- | --- | --- | --- | --- | --- | --- | --- | --- | --- | --- | --- | --- | --- | --- | --- | --- | --- | --- | --- | --- | --- | --- | --- | --- | --- | --- | --- | --- | --- | --- | --- | --- | --- | --- | --- | --- | --- | --- | --- | --- | --- | --- | --- | --- | --- |
| Ratio\_Bats | EPFU | 0 | 0 | 0 | 0.0000 | NA | NA | NA | NA | NA | NA | NA | NA | NA | NA | NA | NA | NA | NA | NA | NA | NA | NA | NA | NA | NA | NA | NA | NA | NA | NA | NA | NA | NA | NA | NA | NA | NA | NA | NA | NA | NA | NA | NA | NA | NA | NA | NA |
| log\_Ratio\_Bats | EPFU | 0 | 0 | 0 | 0.0000 | 0 | NA | NA | NA | NA | NA | NA | NA | NA | NA | NA | NA | NA | NA | NA | NA | NA | NA | NA | NA | NA | NA | NA | NA | NA | NA | NA | NA | NA | NA | NA | NA | NA | NA | NA | NA | NA | NA | NA | NA | NA | NA | NA |
| INT\_log\_Ratio\_Bats | EPFU | 0 | 0 | 0 | 0.0000 | 0 | 0.0703 | NA | NA | NA | NA | NA | NA | NA | NA | NA | NA | NA | NA | NA | NA | NA | NA | NA | NA | NA | NA | NA | NA | NA | NA | NA | NA | NA | NA | NA | NA | NA | NA | NA | NA | NA | NA | NA | NA | NA | NA | NA |
| sq\_Ratio\_Bats | EPFU | 0 | 0 | 0 | 0.0000 | NA | NA | 0 | NA | NA | NA | NA | NA | NA | NA | NA | NA | NA | NA | NA | NA | NA | NA | NA | NA | NA | NA | NA | NA | NA | NA | NA | NA | NA | NA | NA | NA | NA | NA | NA | NA | NA | NA | NA | NA | NA | NA | NA |
| INT\_sq\_Ratio\_Bats | EPFU | 0 | 0 | 0 | 0.0000 | NA | 0.0000 | 0 | NA | NA | NA | NA | NA | NA | NA | NA | NA | NA | NA | NA | NA | NA | NA | NA | NA | NA | NA | NA | NA | NA | NA | NA | NA | NA | NA | NA | NA | NA | NA | NA | NA | NA | NA | NA | NA | NA | NA | NA |
| Ratio\_High/Low | EPFU | 0 | 0 | 0 | 0.0000 | NA | NA | NA | 0.0000 | NA | NA | NA | NA | NA | NA | NA | NA | NA | NA | NA | NA | NA | NA | NA | NA | NA | NA | NA | NA | NA | NA | NA | NA | NA | NA | NA | NA | NA | NA | NA | NA | NA | NA | NA | NA | NA | NA | NA |
| log\_Ratio\_High/Low | EPFU | 0 | 0 | 0 | 0.0008 | NA | NA | NA | 0.0000 | 0 | NA | NA | NA | NA | NA | NA | NA | NA | NA | NA | NA | NA | NA | NA | NA | NA | NA | NA | NA | NA | NA | NA | NA | NA | NA | NA | NA | NA | NA | NA | NA | NA | NA | NA | NA | NA | NA | NA |
| INT\_log\_Ratio\_High/Low | EPFU | 0 | 0 | 0 | 0.0000 | NA | NA | NA | 0.0438 | 0 | 0.0000 | NA | NA | NA | NA | NA | NA | NA | NA | NA | NA | NA | NA | NA | NA | NA | NA | NA | NA | NA | NA | NA | NA | NA | NA | NA | NA | NA | NA | NA | NA | NA | NA | NA | NA | NA | NA | NA |
| sq\_Ratio\_High/Low | EPFU | 0 | 0 | 0 | 0.0000 | NA | NA | NA | 0.0000 | NA | NA | 0 | NA | NA | NA | NA | NA | NA | NA | NA | NA | NA | NA | NA | NA | NA | NA | NA | NA | NA | NA | NA | NA | NA | NA | NA | NA | NA | NA | NA | NA | NA | NA | NA | NA | NA | NA | NA |
| INT\_sq\_Ratio\_High/Low | EPFU | 0 | 0 | 0 | 0.0000 | NA | NA | NA | 0.0000 | NA | 0.7476 | 0 | NA | NA | NA | NA | NA | NA | NA | NA | NA | NA | NA | NA | NA | NA | NA | NA | NA | NA | NA | NA | NA | NA | NA | NA | NA | NA | NA | NA | NA | NA | NA | NA | NA | NA | NA | NA |
| Ratio\_SPECIES | EPFU | 0 | 0 | 0 | 0.0000 | NA | NA | NA | NA | NA | NA | NA | NA | 0.0231 | 0.0740 | 0.8713 | 0.0067 | 0.0067 | 0.0740 | 0.0067 | 0.0067 | NA | NA | NA | NA | NA | NA | NA | NA | NA | NA | NA | NA | NA | NA | NA | NA | NA | NA | NA | NA | NA | NA | NA | NA | NA | NA | NA |
| log\_Ratio\_SPECIES | EPFU | 0 | 0 | 0 | 0.0000 | NA | NA | NA | NA | NA | NA | NA | NA | 0.4977 | 0.0044 | 0.0000 | 0.1157 | 0.1157 | 0.0044 | 0.1157 | 0.1157 | NA | 0.0008 | 0.0000 | 0.0000 | 0.7658 | 0.7658 | 0.0000 | 0.7658 | 0.7658 | NA | NA | NA | NA | NA | NA | NA | NA | NA | NA | NA | NA | NA | NA | NA | NA | NA | NA |
| INT\_log\_Ratio\_SPECIES | EPFU | 0 | 0 | 0 | 0.0000 | NA | NA | NA | NA | NA | NA | NA | NA | 0.4614 | 0.1567 | 0.0004 | 0.9670 | 0.9670 | 0.1567 | 0.9670 | 0.9670 | NA | 0.0013 | 0.0000 | 0.0000 | 0.9069 | 0.9069 | 0.0000 | 0.9069 | 0.9069 | NA | 0.5875 | 0.5352 | 0.4640 | 0.7000 | 0.7000 | 0.5352 | 0.7000 | 0.7000 | NA | NA | NA | NA | NA | NA | NA | NA | NA |
| sq\_Ratio\_SPECIES | EPFU | 0 | 0 | 0 | 0.0000 | NA | NA | NA | NA | NA | NA | NA | NA | 0.0640 | 0.4489 | 0.0029 | 0.0039 | 0.0039 | 0.4489 | 0.0039 | 0.0039 | NA | NA | NA | NA | NA | NA | NA | NA | NA | NA | NA | NA | NA | NA | NA | NA | NA | NA | NA | 0.4287 | 0.8208 | 0.0002 | 0.0785 | 0.0785 | 0.8208 | 0.0785 | 0.0785 |
| INT\_sq\_Ratio\_SPECIES | EPFU | 0 | 0 | 0 | 0.0000 | NA | NA | NA | NA | NA | NA | NA | NA | 0.2926 | 0.6000 | 0.0000 | 0.0100 | 0.0100 | 0.6000 | 0.0100 | 0.0100 | NA | NA | NA | NA | NA | NA | NA | NA | NA | NA | 0.7015 | 0.1304 | 0.0000 | 0.4642 | 0.4642 | 0.1304 | 0.4642 | 0.4642 | NA | 0.3374 | 0.4637 | 0.9485 | 0.2493 | 0.2493 | 0.4637 | 0.2493 | 0.2493 |
| Ratio\_Bats | LABO | 0 | 0 | 0 | 0.0000 | NA | NA | NA | NA | NA | NA | NA | NA | NA | NA | NA | NA | NA | NA | NA | NA | NA | NA | NA | NA | NA | NA | NA | NA | NA | NA | NA | NA | NA | NA | NA | NA | NA | NA | NA | NA | NA | NA | NA | NA | NA | NA | NA |
| log\_Ratio\_Bats | LABO | 0 | 0 | 0 | 0.0000 | 0 | NA | NA | NA | NA | NA | NA | NA | NA | NA | NA | NA | NA | NA | NA | NA | NA | NA | NA | NA | NA | NA | NA | NA | NA | NA | NA | NA | NA | NA | NA | NA | NA | NA | NA | NA | NA | NA | NA | NA | NA | NA | NA |
| INT\_log\_Ratio\_Bats | LABO | 0 | 0 | 0 | 0.2000 | 0 | 0.0000 | NA | NA | NA | NA | NA | NA | NA | NA | NA | NA | NA | NA | NA | NA | NA | NA | NA | NA | NA | NA | NA | NA | NA | NA | NA | NA | NA | NA | NA | NA | NA | NA | NA | NA | NA | NA | NA | NA | NA | NA | NA |
| sq\_Ratio\_Bats | LABO | 0 | 0 | 0 | 0.0000 | NA | NA | 0 | NA | NA | NA | NA | NA | NA | NA | NA | NA | NA | NA | NA | NA | NA | NA | NA | NA | NA | NA | NA | NA | NA | NA | NA | NA | NA | NA | NA | NA | NA | NA | NA | NA | NA | NA | NA | NA | NA | NA | NA |
| INT\_sq\_Ratio\_Bats | LABO | 0 | 0 | 0 | 0.0000 | NA | 0.0000 | 0 | NA | NA | NA | NA | NA | NA | NA | NA | NA | NA | NA | NA | NA | NA | NA | NA | NA | NA | NA | NA | NA | NA | NA | NA | NA | NA | NA | NA | NA | NA | NA | NA | NA | NA | NA | NA | NA | NA | NA | NA |
| Ratio\_High/Low | LABO | 0 | 0 | 0 | 0.0000 | NA | NA | NA | 0.0000 | NA | NA | NA | NA | NA | NA | NA | NA | NA | NA | NA | NA | NA | NA | NA | NA | NA | NA | NA | NA | NA | NA | NA | NA | NA | NA | NA | NA | NA | NA | NA | NA | NA | NA | NA | NA | NA | NA | NA |
| log\_Ratio\_High/Low | LABO | 0 | 0 | 0 | 0.5231 | NA | NA | NA | 0.1936 | 0 | NA | NA | NA | NA | NA | NA | NA | NA | NA | NA | NA | NA | NA | NA | NA | NA | NA | NA | NA | NA | NA | NA | NA | NA | NA | NA | NA | NA | NA | NA | NA | NA | NA | NA | NA | NA | NA | NA |
| INT\_log\_Ratio\_High/Low | LABO | 0 | 0 | 0 | 0.4328 | NA | NA | NA | 0.1633 | 0 | 0.0000 | NA | NA | NA | NA | NA | NA | NA | NA | NA | NA | NA | NA | NA | NA | NA | NA | NA | NA | NA | NA | NA | NA | NA | NA | NA | NA | NA | NA | NA | NA | NA | NA | NA | NA | NA | NA | NA |
| sq\_Ratio\_High/Low | LABO | 0 | 0 | 0 | 0.2118 | NA | NA | NA | 0.0000 | NA | NA | 0 | NA | NA | NA | NA | NA | NA | NA | NA | NA | NA | NA | NA | NA | NA | NA | NA | NA | NA | NA | NA | NA | NA | NA | NA | NA | NA | NA | NA | NA | NA | NA | NA | NA | NA | NA | NA |
| INT\_sq\_Ratio\_High/Low | LABO | 0 | 0 | 0 | 0.0341 | NA | NA | NA | 0.0000 | NA | 0.0000 | 0 | NA | NA | NA | NA | NA | NA | NA | NA | NA | NA | NA | NA | NA | NA | NA | NA | NA | NA | NA | NA | NA | NA | NA | NA | NA | NA | NA | NA | NA | NA | NA | NA | NA | NA | NA | NA |
| Ratio\_SPECIES | LABO | 0 | 0 | 0 | 0.0000 | NA | NA | NA | NA | NA | NA | NA | 0.0967 | NA | 0.0967 | 0.0967 | 0.1585 | 0.4446 | 0.0967 | 0.2469 | 0.9800 | NA | NA | NA | NA | NA | NA | NA | NA | NA | NA | NA | NA | NA | NA | NA | NA | NA | NA | NA | NA | NA | NA | NA | NA | NA | NA | NA |
| log\_Ratio\_SPECIES | LABO | 0 | 0 | 0 | 0.0007 | NA | NA | NA | NA | NA | NA | NA | 0.3869 | NA | 0.3869 | 0.3869 | 0.9738 | 0.0080 | 0.3869 | 0.2976 | 0.0000 | 0.8811 | NA | 0.8811 | 0.8811 | 0.1969 | 0.0000 | 0.8811 | 0.0021 | 0.0000 | NA | NA | NA | NA | NA | NA | NA | NA | NA | NA | NA | NA | NA | NA | NA | NA | NA | NA |
| INT\_log\_Ratio\_SPECIES | LABO | 0 | 0 | 0 | 0.0007 | NA | NA | NA | NA | NA | NA | NA | 0.7645 | NA | 0.7645 | 0.7645 | 0.9441 | 0.4838 | 0.7645 | 0.7902 | 0.1415 | 0.8756 | NA | 0.8756 | 0.8756 | 0.2629 | 0.0000 | 0.8756 | 0.0065 | 0.0000 | 0.9533 | NA | 0.9533 | 0.9533 | 0.9516 | 0.9713 | 0.9533 | 0.9827 | 0.9467 | NA | NA | NA | NA | NA | NA | NA | NA | NA |
| sq\_Ratio\_SPECIES | LABO | 0 | 0 | 0 | 0.0000 | NA | NA | NA | NA | NA | NA | NA | 0.1580 | NA | 0.1580 | 0.1580 | 0.3641 | 0.5678 | 0.1580 | 0.7463 | 0.0262 | NA | NA | NA | NA | NA | NA | NA | NA | NA | NA | NA | NA | NA | NA | NA | NA | NA | NA | 0.3930 | NA | 0.3930 | 0.3930 | 0.6913 | 0.3258 | 0.3930 | 0.8815 | 0.0119 |
| INT\_sq\_Ratio\_SPECIES | LABO | 0 | 0 | 0 | 0.0000 | NA | NA | NA | NA | NA | NA | NA | 0.2342 | NA | 0.2342 | 0.2342 | 0.6231 | 0.1062 | 0.2342 | 0.7458 | 0.0001 | NA | NA | NA | NA | NA | NA | NA | NA | NA | 0.8537 | NA | 0.8537 | 0.8537 | 0.7644 | 0.0722 | 0.8537 | 0.3845 | 0.0006 | 0.4872 | NA | 0.4872 | 0.4872 | 0.5613 | 0.7966 | 0.4872 | 0.6598 | 0.9137 |
| Ratio\_Bats | LACI | 0 | 0 | 0 | 0.0000 | NA | NA | NA | NA | NA | NA | NA | NA | NA | NA | NA | NA | NA | NA | NA | NA | NA | NA | NA | NA | NA | NA | NA | NA | NA | NA | NA | NA | NA | NA | NA | NA | NA | NA | NA | NA | NA | NA | NA | NA | NA | NA | NA |
| log\_Ratio\_Bats | LACI | 0 | 0 | 0 | 0.0000 | 0 | NA | NA | NA | NA | NA | NA | NA | NA | NA | NA | NA | NA | NA | NA | NA | NA | NA | NA | NA | NA | NA | NA | NA | NA | NA | NA | NA | NA | NA | NA | NA | NA | NA | NA | NA | NA | NA | NA | NA | NA | NA | NA |
| INT\_log\_Ratio\_Bats | LACI | 0 | 0 | 0 | 0.0000 | 0 | 0.2735 | NA | NA | NA | NA | NA | NA | NA | NA | NA | NA | NA | NA | NA | NA | NA | NA | NA | NA | NA | NA | NA | NA | NA | NA | NA | NA | NA | NA | NA | NA | NA | NA | NA | NA | NA | NA | NA | NA | NA | NA | NA |
| sq\_Ratio\_Bats | LACI | 0 | 0 | 0 | 0.0000 | NA | NA | 0 | NA | NA | NA | NA | NA | NA | NA | NA | NA | NA | NA | NA | NA | NA | NA | NA | NA | NA | NA | NA | NA | NA | NA | NA | NA | NA | NA | NA | NA | NA | NA | NA | NA | NA | NA | NA | NA | NA | NA | NA |
| INT\_sq\_Ratio\_Bats | LACI | 0 | 0 | 0 | 0.0000 | NA | 0.0000 | 0 | NA | NA | NA | NA | NA | NA | NA | NA | NA | NA | NA | NA | NA | NA | NA | NA | NA | NA | NA | NA | NA | NA | NA | NA | NA | NA | NA | NA | NA | NA | NA | NA | NA | NA | NA | NA | NA | NA | NA | NA |
| Ratio\_High/Low | LACI | 0 | 0 | 0 | 0.0000 | NA | NA | NA | 0.0000 | NA | NA | NA | NA | NA | NA | NA | NA | NA | NA | NA | NA | NA | NA | NA | NA | NA | NA | NA | NA | NA | NA | NA | NA | NA | NA | NA | NA | NA | NA | NA | NA | NA | NA | NA | NA | NA | NA | NA |
| log\_Ratio\_High/Low | LACI | 0 | 0 | 0 | 0.0130 | NA | NA | NA | 0.0000 | 0 | NA | NA | NA | NA | NA | NA | NA | NA | NA | NA | NA | NA | NA | NA | NA | NA | NA | NA | NA | NA | NA | NA | NA | NA | NA | NA | NA | NA | NA | NA | NA | NA | NA | NA | NA | NA | NA | NA |
| INT\_log\_Ratio\_High/Low | LACI | 0 | 0 | 0 | 0.0078 | NA | NA | NA | 0.0000 | 0 | 0.2522 | NA | NA | NA | NA | NA | NA | NA | NA | NA | NA | NA | NA | NA | NA | NA | NA | NA | NA | NA | NA | NA | NA | NA | NA | NA | NA | NA | NA | NA | NA | NA | NA | NA | NA | NA | NA | NA |
| sq\_Ratio\_High/Low | LACI | 0 | 0 | 0 | 0.0000 | NA | NA | NA | 0.0000 | NA | NA | 0 | NA | NA | NA | NA | NA | NA | NA | NA | NA | NA | NA | NA | NA | NA | NA | NA | NA | NA | NA | NA | NA | NA | NA | NA | NA | NA | NA | NA | NA | NA | NA | NA | NA | NA | NA | NA |
| INT\_sq\_Ratio\_High/Low | LACI | 0 | 0 | 0 | 0.0000 | NA | NA | NA | 0.0000 | NA | 0.8916 | 0 | NA | NA | NA | NA | NA | NA | NA | NA | NA | NA | NA | NA | NA | NA | NA | NA | NA | NA | NA | NA | NA | NA | NA | NA | NA | NA | NA | NA | NA | NA | NA | NA | NA | NA | NA | NA |
| Ratio\_SPECIES | LACI | 0 | 0 | 0 | 0.0000 | NA | NA | NA | NA | NA | NA | NA | 0.6291 | 0.0686 | NA | 0.9742 | 0.0686 | 0.0686 | 0.0686 | 0.0686 | 0.0686 | NA | NA | NA | NA | NA | NA | NA | NA | NA | NA | NA | NA | NA | NA | NA | NA | NA | NA | NA | NA | NA | NA | NA | NA | NA | NA | NA |
| log\_Ratio\_SPECIES | LACI | 0 | 0 | 0 | 0.0000 | NA | NA | NA | NA | NA | NA | NA | 0.0000 | 0.2915 | NA | 0.0000 | 0.2915 | 0.2915 | 0.2915 | 0.2915 | 0.2915 | 0.0000 | 0.9441 | NA | 0.0000 | 0.9441 | 0.9441 | 0.9441 | 0.9441 | 0.9441 | NA | NA | NA | NA | NA | NA | NA | NA | NA | NA | NA | NA | NA | NA | NA | NA | NA | NA |
| INT\_log\_Ratio\_SPECIES | LACI | 0 | 0 | 0 | 0.0001 | NA | NA | NA | NA | NA | NA | NA | 0.0078 | 0.7169 | NA | 0.0018 | 0.7169 | 0.7169 | 0.7169 | 0.7169 | 0.7169 | 0.0000 | 0.7537 | NA | 0.0000 | 0.7537 | 0.7537 | 0.7537 | 0.7537 | 0.7537 | 0.2593 | 0.5206 | NA | 0.2801 | 0.5206 | 0.5206 | 0.5206 | 0.5206 | 0.5206 | NA | NA | NA | NA | NA | NA | NA | NA | NA |
| sq\_Ratio\_SPECIES | LACI | 0 | 0 | 0 | 0.0000 | NA | NA | NA | NA | NA | NA | NA | 0.3853 | 0.0239 | NA | 0.0609 | 0.0239 | 0.0239 | 0.0239 | 0.0239 | 0.0239 | NA | NA | NA | NA | NA | NA | NA | NA | NA | NA | NA | NA | NA | NA | NA | NA | NA | NA | 0.1234 | 0.1651 | NA | 0.0134 | 0.1651 | 0.1651 | 0.1651 | 0.1651 | 0.1651 |
| INT\_sq\_Ratio\_SPECIES | LACI | 0 | 0 | 0 | 0.0000 | NA | NA | NA | NA | NA | NA | NA | 0.0136 | 0.0362 | NA | 0.0001 | 0.0362 | 0.0362 | 0.0362 | 0.0362 | 0.0362 | NA | NA | NA | NA | NA | NA | NA | NA | NA | 0.0109 | 0.4431 | NA | 0.0003 | 0.4431 | 0.4431 | 0.4431 | 0.4431 | 0.4431 | 0.8840 | 0.4498 | NA | 0.9680 | 0.4498 | 0.4498 | 0.4498 | 0.4498 | 0.4498 |
| Ratio\_Bats | LANO | 0 | 0 | 0 | 0.0000 | NA | NA | NA | NA | NA | NA | NA | NA | NA | NA | NA | NA | NA | NA | NA | NA | NA | NA | NA | NA | NA | NA | NA | NA | NA | NA | NA | NA | NA | NA | NA | NA | NA | NA | NA | NA | NA | NA | NA | NA | NA | NA | NA |
| log\_Ratio\_Bats | LANO | 0 | 0 | 0 | 0.0000 | 0 | NA | NA | NA | NA | NA | NA | NA | NA | NA | NA | NA | NA | NA | NA | NA | NA | NA | NA | NA | NA | NA | NA | NA | NA | NA | NA | NA | NA | NA | NA | NA | NA | NA | NA | NA | NA | NA | NA | NA | NA | NA | NA |
| INT\_log\_Ratio\_Bats | LANO | 0 | 0 | 0 | 0.0044 | 0 | 0.0000 | NA | NA | NA | NA | NA | NA | NA | NA | NA | NA | NA | NA | NA | NA | NA | NA | NA | NA | NA | NA | NA | NA | NA | NA | NA | NA | NA | NA | NA | NA | NA | NA | NA | NA | NA | NA | NA | NA | NA | NA | NA |
| sq\_Ratio\_Bats | LANO | 0 | 0 | 0 | 0.0000 | NA | NA | 0 | NA | NA | NA | NA | NA | NA | NA | NA | NA | NA | NA | NA | NA | NA | NA | NA | NA | NA | NA | NA | NA | NA | NA | NA | NA | NA | NA | NA | NA | NA | NA | NA | NA | NA | NA | NA | NA | NA | NA | NA |
| INT\_sq\_Ratio\_Bats | LANO | 0 | 0 | 0 | 0.0000 | NA | 0.0000 | 0 | NA | NA | NA | NA | NA | NA | NA | NA | NA | NA | NA | NA | NA | NA | NA | NA | NA | NA | NA | NA | NA | NA | NA | NA | NA | NA | NA | NA | NA | NA | NA | NA | NA | NA | NA | NA | NA | NA | NA | NA |
| Ratio\_High/Low | LANO | 0 | 0 | 0 | 0.0000 | NA | NA | NA | 0.0000 | NA | NA | NA | NA | NA | NA | NA | NA | NA | NA | NA | NA | NA | NA | NA | NA | NA | NA | NA | NA | NA | NA | NA | NA | NA | NA | NA | NA | NA | NA | NA | NA | NA | NA | NA | NA | NA | NA | NA |
| log\_Ratio\_High/Low | LANO | 0 | 0 | 0 | 0.0093 | NA | NA | NA | 0.0001 | 0 | NA | NA | NA | NA | NA | NA | NA | NA | NA | NA | NA | NA | NA | NA | NA | NA | NA | NA | NA | NA | NA | NA | NA | NA | NA | NA | NA | NA | NA | NA | NA | NA | NA | NA | NA | NA | NA | NA |
| INT\_log\_Ratio\_High/Low | LANO | 0 | 0 | 0 | 0.9385 | NA | NA | NA | 0.0000 | 0 | 0.0000 | NA | NA | NA | NA | NA | NA | NA | NA | NA | NA | NA | NA | NA | NA | NA | NA | NA | NA | NA | NA | NA | NA | NA | NA | NA | NA | NA | NA | NA | NA | NA | NA | NA | NA | NA | NA | NA |
| sq\_Ratio\_High/Low | LANO | 0 | 0 | 0 | 0.0000 | NA | NA | NA | 0.0000 | NA | NA | 0 | NA | NA | NA | NA | NA | NA | NA | NA | NA | NA | NA | NA | NA | NA | NA | NA | NA | NA | NA | NA | NA | NA | NA | NA | NA | NA | NA | NA | NA | NA | NA | NA | NA | NA | NA | NA |
| INT\_sq\_Ratio\_High/Low | LANO | 0 | 0 | 0 | 0.0000 | NA | NA | NA | 0.0000 | NA | 0.0000 | 0 | NA | NA | NA | NA | NA | NA | NA | NA | NA | NA | NA | NA | NA | NA | NA | NA | NA | NA | NA | NA | NA | NA | NA | NA | NA | NA | NA | NA | NA | NA | NA | NA | NA | NA | NA | NA |
| Ratio\_SPECIES | LANO | 0 | 0 | 0 | 0.0000 | NA | NA | NA | NA | NA | NA | NA | 0.4123 | 0.0048 | 0.8886 | NA | 0.0010 | 0.0010 | 0.0010 | 0.0010 | 0.0010 | NA | NA | NA | NA | NA | NA | NA | NA | NA | NA | NA | NA | NA | NA | NA | NA | NA | NA | NA | NA | NA | NA | NA | NA | NA | NA | NA |
| log\_Ratio\_SPECIES | LANO | 0 | 0 | 0 | 0.0000 | NA | NA | NA | NA | NA | NA | NA | 0.0000 | 0.6097 | 0.0000 | NA | 0.0545 | 0.0545 | 0.0545 | 0.0545 | 0.0545 | 0.0000 | 0.0005 | 0.0000 | NA | 0.6390 | 0.6390 | 0.6390 | 0.6390 | 0.6390 | NA | NA | NA | NA | NA | NA | NA | NA | NA | NA | NA | NA | NA | NA | NA | NA | NA | NA |
| INT\_log\_Ratio\_SPECIES | LANO | 0 | 0 | 0 | 0.0000 | NA | NA | NA | NA | NA | NA | NA | 0.0028 | 0.6725 | 0.0002 | NA | 0.6902 | 0.6902 | 0.6902 | 0.6902 | 0.6902 | 0.0000 | 0.0013 | 0.0000 | NA | 0.7131 | 0.7131 | 0.7131 | 0.7131 | 0.7131 | 0.7223 | 0.7785 | 0.7500 | NA | 0.8958 | 0.8958 | 0.8958 | 0.8958 | 0.8958 | NA | NA | NA | NA | NA | NA | NA | NA | NA |
| sq\_Ratio\_SPECIES | LANO | 0 | 0 | 0 | 0.0000 | NA | NA | NA | NA | NA | NA | NA | 0.0119 | 0.0425 | 0.0000 | NA | 0.0019 | 0.0019 | 0.0019 | 0.0019 | 0.0019 | NA | NA | NA | NA | NA | NA | NA | NA | NA | NA | NA | NA | NA | NA | NA | NA | NA | NA | 0.0008 | 0.3804 | 0.0000 | NA | 0.0592 | 0.0592 | 0.0592 | 0.0592 | 0.0592 |
| INT\_sq\_Ratio\_SPECIES | LANO | 0 | 0 | 0 | 0.0000 | NA | NA | NA | NA | NA | NA | NA | 0.0000 | 0.2618 | 0.0000 | NA | 0.0064 | 0.0064 | 0.0064 | 0.0064 | 0.0064 | NA | NA | NA | NA | NA | NA | NA | NA | NA | 0.0000 | 0.5756 | 0.0000 | NA | 0.5364 | 0.5364 | 0.5364 | 0.5364 | 0.5364 | 0.8481 | 0.2354 | 0.8543 | NA | 0.1660 | 0.1660 | 0.1660 | 0.1660 | 0.1660 |
| Ratio\_Bats | MYLE | 0 | 0 | 0 | 0.0000 | NA | NA | NA | NA | NA | NA | NA | NA | NA | NA | NA | NA | NA | NA | NA | NA | NA | NA | NA | NA | NA | NA | NA | NA | NA | NA | NA | NA | NA | NA | NA | NA | NA | NA | NA | NA | NA | NA | NA | NA | NA | NA | NA |
| log\_Ratio\_Bats | MYLE | 0 | 0 | 0 | 0.0000 | 0 | NA | NA | NA | NA | NA | NA | NA | NA | NA | NA | NA | NA | NA | NA | NA | NA | NA | NA | NA | NA | NA | NA | NA | NA | NA | NA | NA | NA | NA | NA | NA | NA | NA | NA | NA | NA | NA | NA | NA | NA | NA | NA |
| INT\_log\_Ratio\_Bats | MYLE | 0 | 0 | 0 | 0.9513 | 0 | 0.0000 | NA | NA | NA | NA | NA | NA | NA | NA | NA | NA | NA | NA | NA | NA | NA | NA | NA | NA | NA | NA | NA | NA | NA | NA | NA | NA | NA | NA | NA | NA | NA | NA | NA | NA | NA | NA | NA | NA | NA | NA | NA |
| sq\_Ratio\_Bats | MYLE | 0 | 0 | 0 | 0.0000 | NA | NA | 0 | NA | NA | NA | NA | NA | NA | NA | NA | NA | NA | NA | NA | NA | NA | NA | NA | NA | NA | NA | NA | NA | NA | NA | NA | NA | NA | NA | NA | NA | NA | NA | NA | NA | NA | NA | NA | NA | NA | NA | NA |
| INT\_sq\_Ratio\_Bats | MYLE | 0 | 0 | 0 | 0.0000 | NA | 0.0000 | 0 | NA | NA | NA | NA | NA | NA | NA | NA | NA | NA | NA | NA | NA | NA | NA | NA | NA | NA | NA | NA | NA | NA | NA | NA | NA | NA | NA | NA | NA | NA | NA | NA | NA | NA | NA | NA | NA | NA | NA | NA |
| Ratio\_High/Low | MYLE | 0 | 0 | 0 | 0.0000 | NA | NA | NA | 0.0000 | NA | NA | NA | NA | NA | NA | NA | NA | NA | NA | NA | NA | NA | NA | NA | NA | NA | NA | NA | NA | NA | NA | NA | NA | NA | NA | NA | NA | NA | NA | NA | NA | NA | NA | NA | NA | NA | NA | NA |
| log\_Ratio\_High/Low | MYLE | 0 | 0 | 0 | 0.6991 | NA | NA | NA | 0.0010 | 0 | NA | NA | NA | NA | NA | NA | NA | NA | NA | NA | NA | NA | NA | NA | NA | NA | NA | NA | NA | NA | NA | NA | NA | NA | NA | NA | NA | NA | NA | NA | NA | NA | NA | NA | NA | NA | NA | NA |
| INT\_log\_Ratio\_High/Low | MYLE | 0 | 0 | 0 | 0.0641 | NA | NA | NA | 0.8202 | 0 | 0.0000 | NA | NA | NA | NA | NA | NA | NA | NA | NA | NA | NA | NA | NA | NA | NA | NA | NA | NA | NA | NA | NA | NA | NA | NA | NA | NA | NA | NA | NA | NA | NA | NA | NA | NA | NA | NA | NA |
| sq\_Ratio\_High/Low | MYLE | 0 | 0 | 0 | 0.0376 | NA | NA | NA | 0.0000 | NA | NA | 0 | NA | NA | NA | NA | NA | NA | NA | NA | NA | NA | NA | NA | NA | NA | NA | NA | NA | NA | NA | NA | NA | NA | NA | NA | NA | NA | NA | NA | NA | NA | NA | NA | NA | NA | NA | NA |
| INT\_sq\_Ratio\_High/Low | MYLE | 0 | 0 | 0 | 0.0017 | NA | NA | NA | 0.0000 | NA | 0.0000 | 0 | NA | NA | NA | NA | NA | NA | NA | NA | NA | NA | NA | NA | NA | NA | NA | NA | NA | NA | NA | NA | NA | NA | NA | NA | NA | NA | NA | NA | NA | NA | NA | NA | NA | NA | NA | NA |
| Ratio\_SPECIES | MYLE | 0 | 0 | 0 | 0.0000 | NA | NA | NA | NA | NA | NA | NA | 0.0334 | 0.0459 | 0.0334 | 0.0334 | NA | 0.1268 | 0.2790 | 0.8797 | 0.0459 | NA | NA | NA | NA | NA | NA | NA | NA | NA | NA | NA | NA | NA | NA | NA | NA | NA | NA | NA | NA | NA | NA | NA | NA | NA | NA | NA |
| log\_Ratio\_SPECIES | MYLE | 0 | 0 | 0 | 0.0000 | NA | NA | NA | NA | NA | NA | NA | 0.2095 | 0.6264 | 0.2095 | 0.2095 | NA | 0.1196 | 0.0004 | 0.0000 | 0.6264 | 0.7360 | 0.3744 | 0.7360 | 0.7360 | NA | 0.0000 | 0.0000 | 0.0000 | 0.3744 | NA | NA | NA | NA | NA | NA | NA | NA | NA | NA | NA | NA | NA | NA | NA | NA | NA | NA |
| INT\_log\_Ratio\_SPECIES | MYLE | 0 | 0 | 0 | 0.0000 | NA | NA | NA | NA | NA | NA | NA | 0.6928 | 0.9377 | 0.6928 | 0.6928 | NA | 0.5839 | 0.2382 | 0.0289 | 0.9377 | 0.7446 | 0.4174 | 0.7446 | 0.7446 | NA | 0.0001 | 0.0000 | 0.0000 | 0.4174 | 0.9594 | 0.9548 | 0.9594 | 0.9594 | NA | 0.9071 | 0.8445 | 0.8777 | 0.9548 | NA | NA | NA | NA | NA | NA | NA | NA | NA |
| sq\_Ratio\_SPECIES | MYLE | 0 | 0 | 0 | 0.0000 | NA | NA | NA | NA | NA | NA | NA | 0.0519 | 0.1027 | 0.0519 | 0.0519 | NA | 0.5689 | 0.6420 | 0.0101 | 0.1027 | NA | NA | NA | NA | NA | NA | NA | NA | NA | NA | NA | NA | NA | NA | NA | NA | NA | NA | 0.2401 | 0.3783 | 0.2401 | 0.2401 | NA | 0.9146 | 0.2822 | 0.0024 | 0.3783 |
| INT\_sq\_Ratio\_SPECIES | MYLE | 0 | 0 | 0 | 0.0000 | NA | NA | NA | NA | NA | NA | NA | 0.0943 | 0.2302 | 0.0943 | 0.0943 | NA | 0.7376 | 0.0704 | 0.0000 | 0.2302 | NA | NA | NA | NA | NA | NA | NA | NA | NA | 0.7401 | 0.9825 | 0.7401 | 0.7401 | NA | 0.2607 | 0.0299 | 0.0000 | 0.9825 | 0.3700 | 0.3973 | 0.3700 | 0.3700 | NA | 0.5287 | 0.6895 | 0.9475 | 0.3973 |
| Ratio\_Bats | MYLU | 0 | 0 | 0 | 0.0000 | NA | NA | NA | NA | NA | NA | NA | NA | NA | NA | NA | NA | NA | NA | NA | NA | NA | NA | NA | NA | NA | NA | NA | NA | NA | NA | NA | NA | NA | NA | NA | NA | NA | NA | NA | NA | NA | NA | NA | NA | NA | NA | NA |
| log\_Ratio\_Bats | MYLU | 0 | 0 | 0 | 0.0000 | 0 | NA | NA | NA | NA | NA | NA | NA | NA | NA | NA | NA | NA | NA | NA | NA | NA | NA | NA | NA | NA | NA | NA | NA | NA | NA | NA | NA | NA | NA | NA | NA | NA | NA | NA | NA | NA | NA | NA | NA | NA | NA | NA |
| INT\_log\_Ratio\_Bats | MYLU | 0 | 0 | 0 | 0.0040 | 0 | 0.0000 | NA | NA | NA | NA | NA | NA | NA | NA | NA | NA | NA | NA | NA | NA | NA | NA | NA | NA | NA | NA | NA | NA | NA | NA | NA | NA | NA | NA | NA | NA | NA | NA | NA | NA | NA | NA | NA | NA | NA | NA | NA |
| sq\_Ratio\_Bats | MYLU | 0 | 0 | 0 | 0.0000 | NA | NA | 0 | NA | NA | NA | NA | NA | NA | NA | NA | NA | NA | NA | NA | NA | NA | NA | NA | NA | NA | NA | NA | NA | NA | NA | NA | NA | NA | NA | NA | NA | NA | NA | NA | NA | NA | NA | NA | NA | NA | NA | NA |
| INT\_sq\_Ratio\_Bats | MYLU | 0 | 0 | 0 | 0.0000 | NA | 0.0000 | 0 | NA | NA | NA | NA | NA | NA | NA | NA | NA | NA | NA | NA | NA | NA | NA | NA | NA | NA | NA | NA | NA | NA | NA | NA | NA | NA | NA | NA | NA | NA | NA | NA | NA | NA | NA | NA | NA | NA | NA | NA |
| Ratio\_High/Low | MYLU | 0 | 0 | 0 | 0.0000 | NA | NA | NA | 0.0000 | NA | NA | NA | NA | NA | NA | NA | NA | NA | NA | NA | NA | NA | NA | NA | NA | NA | NA | NA | NA | NA | NA | NA | NA | NA | NA | NA | NA | NA | NA | NA | NA | NA | NA | NA | NA | NA | NA | NA |
| log\_Ratio\_High/Low | MYLU | 0 | 0 | 0 | 0.1923 | NA | NA | NA | 0.0466 | 0 | NA | NA | NA | NA | NA | NA | NA | NA | NA | NA | NA | NA | NA | NA | NA | NA | NA | NA | NA | NA | NA | NA | NA | NA | NA | NA | NA | NA | NA | NA | NA | NA | NA | NA | NA | NA | NA | NA |
| INT\_log\_Ratio\_High/Low | MYLU | 0 | 0 | 0 | 0.1587 | NA | NA | NA | 0.0020 | 0 | 0.0000 | NA | NA | NA | NA | NA | NA | NA | NA | NA | NA | NA | NA | NA | NA | NA | NA | NA | NA | NA | NA | NA | NA | NA | NA | NA | NA | NA | NA | NA | NA | NA | NA | NA | NA | NA | NA | NA |
| sq\_Ratio\_High/Low | MYLU | 0 | 0 | 0 | 0.0013 | NA | NA | NA | 0.0000 | NA | NA | 0 | NA | NA | NA | NA | NA | NA | NA | NA | NA | NA | NA | NA | NA | NA | NA | NA | NA | NA | NA | NA | NA | NA | NA | NA | NA | NA | NA | NA | NA | NA | NA | NA | NA | NA | NA | NA |
| INT\_sq\_Ratio\_High/Low | MYLU | 0 | 0 | 0 | 0.0000 | NA | NA | NA | 0.0000 | NA | 0.0000 | 0 | NA | NA | NA | NA | NA | NA | NA | NA | NA | NA | NA | NA | NA | NA | NA | NA | NA | NA | NA | NA | NA | NA | NA | NA | NA | NA | NA | NA | NA | NA | NA | NA | NA | NA | NA | NA |
| Ratio\_SPECIES | MYLU | 0 | 0 | 0 | 0.0000 | NA | NA | NA | NA | NA | NA | NA | 0.0017 | 0.0031 | 0.0017 | 0.0017 | 0.0350 | NA | 0.0726 | 0.7734 | 0.0031 | NA | NA | NA | NA | NA | NA | NA | NA | NA | NA | NA | NA | NA | NA | NA | NA | NA | NA | NA | NA | NA | NA | NA | NA | NA | NA | NA |
| log\_Ratio\_SPECIES | MYLU | 0 | 0 | 0 | 0.0000 | NA | NA | NA | NA | NA | NA | NA | 0.1255 | 0.4114 | 0.1255 | 0.1255 | 0.0067 | NA | 0.0001 | 0.0000 | 0.4114 | 0.5171 | 0.5838 | 0.5171 | 0.5171 | 0.0000 | NA | 0.0000 | 0.0000 | 0.5838 | NA | NA | NA | NA | NA | NA | NA | NA | NA | NA | NA | NA | NA | NA | NA | NA | NA | NA |
| INT\_log\_Ratio\_SPECIES | MYLU | 0 | 0 | 0 | 0.0000 | NA | NA | NA | NA | NA | NA | NA | 0.4275 | 0.5640 | 0.4275 | 0.4275 | 0.6760 | NA | 0.5016 | 0.0176 | 0.5640 | 0.4739 | 0.7231 | 0.4739 | 0.4739 | 0.0000 | NA | 0.0000 | 0.0000 | 0.7231 | 0.7194 | 0.7341 | 0.7194 | 0.7194 | 0.7428 | NA | 0.6916 | 0.3978 | 0.7341 | NA | NA | NA | NA | NA | NA | NA | NA | NA |
| sq\_Ratio\_SPECIES | MYLU | 0 | 0 | 0 | 0.0000 | NA | NA | NA | NA | NA | NA | NA | 0.0136 | 0.0361 | 0.0136 | 0.0136 | 0.8451 | NA | 0.6351 | 0.0000 | 0.0361 | NA | NA | NA | NA | NA | NA | NA | NA | NA | NA | NA | NA | NA | NA | NA | NA | NA | NA | 0.1398 | 0.2566 | 0.1398 | 0.1398 | 0.5305 | NA | 0.2138 | 0.0000 | 0.2566 |
| INT\_sq\_Ratio\_SPECIES | MYLU | 0 | 0 | 0 | 0.0000 | NA | NA | NA | NA | NA | NA | NA | 0.0418 | 0.1325 | 0.0418 | 0.0418 | 0.2273 | NA | 0.0299 | 0.0000 | 0.1325 | NA | NA | NA | NA | NA | NA | NA | NA | NA | 0.8541 | 0.8479 | 0.8541 | 0.8541 | 0.0355 | NA | 0.0053 | 0.0000 | 0.8479 | 0.1868 | 0.2153 | 0.1868 | 0.1868 | 0.4186 | NA | 0.5045 | 0.5780 | 0.2153 |
| Ratio\_Bats | MYSE | 0 | 0 | 0 | 0.0000 | NA | NA | NA | NA | NA | NA | NA | NA | NA | NA | NA | NA | NA | NA | NA | NA | NA | NA | NA | NA | NA | NA | NA | NA | NA | NA | NA | NA | NA | NA | NA | NA | NA | NA | NA | NA | NA | NA | NA | NA | NA | NA | NA |
| log\_Ratio\_Bats | MYSE | 0 | 0 | 0 | 0.0000 | 0 | NA | NA | NA | NA | NA | NA | NA | NA | NA | NA | NA | NA | NA | NA | NA | NA | NA | NA | NA | NA | NA | NA | NA | NA | NA | NA | NA | NA | NA | NA | NA | NA | NA | NA | NA | NA | NA | NA | NA | NA | NA | NA |
| INT\_log\_Ratio\_Bats | MYSE | 0 | 0 | 0 | 0.0007 | 0 | 0.5152 | NA | NA | NA | NA | NA | NA | NA | NA | NA | NA | NA | NA | NA | NA | NA | NA | NA | NA | NA | NA | NA | NA | NA | NA | NA | NA | NA | NA | NA | NA | NA | NA | NA | NA | NA | NA | NA | NA | NA | NA | NA |
| sq\_Ratio\_Bats | MYSE | 0 | 0 | 0 | 0.0000 | NA | NA | 0 | NA | NA | NA | NA | NA | NA | NA | NA | NA | NA | NA | NA | NA | NA | NA | NA | NA | NA | NA | NA | NA | NA | NA | NA | NA | NA | NA | NA | NA | NA | NA | NA | NA | NA | NA | NA | NA | NA | NA | NA |
| INT\_sq\_Ratio\_Bats | MYSE | 0 | 0 | 0 | 0.0000 | NA | 0.0000 | 0 | NA | NA | NA | NA | NA | NA | NA | NA | NA | NA | NA | NA | NA | NA | NA | NA | NA | NA | NA | NA | NA | NA | NA | NA | NA | NA | NA | NA | NA | NA | NA | NA | NA | NA | NA | NA | NA | NA | NA | NA |
| Ratio\_High/Low | MYSE | 0 | 0 | 0 | 0.0000 | NA | NA | NA | 0.0000 | NA | NA | NA | NA | NA | NA | NA | NA | NA | NA | NA | NA | NA | NA | NA | NA | NA | NA | NA | NA | NA | NA | NA | NA | NA | NA | NA | NA | NA | NA | NA | NA | NA | NA | NA | NA | NA | NA | NA |
| log\_Ratio\_High/Low | MYSE | 0 | 0 | 0 | 0.0034 | NA | NA | NA | 0.0000 | 0 | NA | NA | NA | NA | NA | NA | NA | NA | NA | NA | NA | NA | NA | NA | NA | NA | NA | NA | NA | NA | NA | NA | NA | NA | NA | NA | NA | NA | NA | NA | NA | NA | NA | NA | NA | NA | NA | NA |
| INT\_log\_Ratio\_High/Low | MYSE | 0 | 0 | 0 | 0.0011 | NA | NA | NA | 0.0000 | 0 | 0.0799 | NA | NA | NA | NA | NA | NA | NA | NA | NA | NA | NA | NA | NA | NA | NA | NA | NA | NA | NA | NA | NA | NA | NA | NA | NA | NA | NA | NA | NA | NA | NA | NA | NA | NA | NA | NA | NA |
| sq\_Ratio\_High/Low | MYSE | 0 | 0 | 0 | 0.0148 | NA | NA | NA | 0.0000 | NA | NA | 0 | NA | NA | NA | NA | NA | NA | NA | NA | NA | NA | NA | NA | NA | NA | NA | NA | NA | NA | NA | NA | NA | NA | NA | NA | NA | NA | NA | NA | NA | NA | NA | NA | NA | NA | NA | NA |
| INT\_sq\_Ratio\_High/Low | MYSE | 0 | 0 | 0 | 0.0018 | NA | NA | NA | 0.0000 | NA | 0.0000 | 0 | NA | NA | NA | NA | NA | NA | NA | NA | NA | NA | NA | NA | NA | NA | NA | NA | NA | NA | NA | NA | NA | NA | NA | NA | NA | NA | NA | NA | NA | NA | NA | NA | NA | NA | NA | NA |
| Ratio\_SPECIES | MYSE | 0 | 0 | 0 | 0.0000 | NA | NA | NA | NA | NA | NA | NA | 0.0934 | 0.1034 | 0.0934 | 0.0934 | 0.4481 | 0.4481 | NA | 0.4481 | 0.1229 | NA | NA | NA | NA | NA | NA | NA | NA | NA | NA | NA | NA | NA | NA | NA | NA | NA | NA | NA | NA | NA | NA | NA | NA | NA | NA | NA |
| log\_Ratio\_SPECIES | MYSE | 0 | 0 | 0 | 0.0000 | NA | NA | NA | NA | NA | NA | NA | 0.2958 | 0.4075 | 0.2958 | 0.2958 | 0.0006 | 0.0006 | NA | 0.0006 | 0.6291 | 0.9374 | 0.7793 | 0.9374 | 0.9374 | 0.0000 | 0.0000 | NA | 0.0000 | 0.3904 | NA | NA | NA | NA | NA | NA | NA | NA | NA | NA | NA | NA | NA | NA | NA | NA | NA | NA |
| INT\_log\_Ratio\_SPECIES | MYSE | 0 | 0 | 0 | 0.0002 | NA | NA | NA | NA | NA | NA | NA | 0.9220 | 0.8702 | 0.9220 | 0.9220 | 0.0981 | 0.0981 | NA | 0.0981 | 0.8039 | 0.9402 | 0.6920 | 0.9402 | 0.9402 | 0.0000 | 0.0000 | NA | 0.0000 | 0.3657 | 0.7056 | 0.7017 | 0.7056 | 0.7056 | 0.4870 | 0.4870 | NA | 0.4870 | 0.7104 | NA | NA | NA | NA | NA | NA | NA | NA | NA |
| sq\_Ratio\_SPECIES | MYSE | 0 | 0 | 0 | 0.0000 | NA | NA | NA | NA | NA | NA | NA | 0.0526 | 0.0685 | 0.0526 | 0.0526 | 0.8403 | 0.8403 | NA | 0.8403 | 0.1009 | NA | NA | NA | NA | NA | NA | NA | NA | NA | NA | NA | NA | NA | NA | NA | NA | NA | NA | 0.2340 | 0.2800 | 0.2340 | 0.2340 | 0.4163 | 0.4163 | NA | 0.4163 | 0.3605 |
| INT\_sq\_Ratio\_SPECIES | MYSE | 0 | 0 | 0 | 0.0000 | NA | NA | NA | NA | NA | NA | NA | 0.0761 | 0.1076 | 0.0761 | 0.0761 | 0.1648 | 0.1648 | NA | 0.1648 | 0.1847 | NA | NA | NA | NA | NA | NA | NA | NA | NA | 0.5562 | 0.6370 | 0.5562 | 0.5562 | 0.0831 | 0.0831 | NA | 0.0831 | 0.7982 | 0.4805 | 0.4973 | 0.4805 | 0.4805 | 0.7482 | 0.7482 | NA | 0.7482 | 0.5093 |
| Ratio\_Bats | MYSO | 0 | 0 | 0 | 0.0000 | NA | NA | NA | NA | NA | NA | NA | NA | NA | NA | NA | NA | NA | NA | NA | NA | NA | NA | NA | NA | NA | NA | NA | NA | NA | NA | NA | NA | NA | NA | NA | NA | NA | NA | NA | NA | NA | NA | NA | NA | NA | NA | NA |
| log\_Ratio\_Bats | MYSO | 0 | 0 | 0 | 0.0000 | 0 | NA | NA | NA | NA | NA | NA | NA | NA | NA | NA | NA | NA | NA | NA | NA | NA | NA | NA | NA | NA | NA | NA | NA | NA | NA | NA | NA | NA | NA | NA | NA | NA | NA | NA | NA | NA | NA | NA | NA | NA | NA | NA |
| INT\_log\_Ratio\_Bats | MYSO | 0 | 0 | 0 | 0.0050 | 0 | 0.0000 | NA | NA | NA | NA | NA | NA | NA | NA | NA | NA | NA | NA | NA | NA | NA | NA | NA | NA | NA | NA | NA | NA | NA | NA | NA | NA | NA | NA | NA | NA | NA | NA | NA | NA | NA | NA | NA | NA | NA | NA | NA |
| sq\_Ratio\_Bats | MYSO | 0 | 0 | 0 | 0.0000 | NA | NA | 0 | NA | NA | NA | NA | NA | NA | NA | NA | NA | NA | NA | NA | NA | NA | NA | NA | NA | NA | NA | NA | NA | NA | NA | NA | NA | NA | NA | NA | NA | NA | NA | NA | NA | NA | NA | NA | NA | NA | NA | NA |
| INT\_sq\_Ratio\_Bats | MYSO | 0 | 0 | 0 | 0.0000 | NA | 0.0000 | 0 | NA | NA | NA | NA | NA | NA | NA | NA | NA | NA | NA | NA | NA | NA | NA | NA | NA | NA | NA | NA | NA | NA | NA | NA | NA | NA | NA | NA | NA | NA | NA | NA | NA | NA | NA | NA | NA | NA | NA | NA |
| Ratio\_High/Low | MYSO | 0 | 0 | 0 | 0.0000 | NA | NA | NA | 0.0000 | NA | NA | NA | NA | NA | NA | NA | NA | NA | NA | NA | NA | NA | NA | NA | NA | NA | NA | NA | NA | NA | NA | NA | NA | NA | NA | NA | NA | NA | NA | NA | NA | NA | NA | NA | NA | NA | NA | NA |
| log\_Ratio\_High/Low | MYSO | 0 | 0 | 0 | 0.2539 | NA | NA | NA | 0.2390 | 0 | NA | NA | NA | NA | NA | NA | NA | NA | NA | NA | NA | NA | NA | NA | NA | NA | NA | NA | NA | NA | NA | NA | NA | NA | NA | NA | NA | NA | NA | NA | NA | NA | NA | NA | NA | NA | NA | NA |
| INT\_log\_Ratio\_High/Low | MYSO | 0 | 0 | 0 | 0.3042 | NA | NA | NA | 0.0047 | 0 | 0.0000 | NA | NA | NA | NA | NA | NA | NA | NA | NA | NA | NA | NA | NA | NA | NA | NA | NA | NA | NA | NA | NA | NA | NA | NA | NA | NA | NA | NA | NA | NA | NA | NA | NA | NA | NA | NA | NA |
| sq\_Ratio\_High/Low | MYSO | 0 | 0 | 0 | 0.0307 | NA | NA | NA | 0.0000 | NA | NA | 0 | NA | NA | NA | NA | NA | NA | NA | NA | NA | NA | NA | NA | NA | NA | NA | NA | NA | NA | NA | NA | NA | NA | NA | NA | NA | NA | NA | NA | NA | NA | NA | NA | NA | NA | NA | NA |
| INT\_sq\_Ratio\_High/Low | MYSO | 0 | 0 | 0 | 0.0007 | NA | NA | NA | 0.0000 | NA | 0.0000 | 0 | NA | NA | NA | NA | NA | NA | NA | NA | NA | NA | NA | NA | NA | NA | NA | NA | NA | NA | NA | NA | NA | NA | NA | NA | NA | NA | NA | NA | NA | NA | NA | NA | NA | NA | NA | NA |
| Ratio\_SPECIES | MYSO | 0 | 0 | 0 | 0.0000 | NA | NA | NA | NA | NA | NA | NA | 0.0229 | 0.0345 | 0.0229 | 0.0229 | 0.0919 | 0.5765 | 0.0919 | NA | 0.0345 | NA | NA | NA | NA | NA | NA | NA | NA | NA | NA | NA | NA | NA | NA | NA | NA | NA | NA | NA | NA | NA | NA | NA | NA | NA | NA | NA |
| log\_Ratio\_SPECIES | MYSO | 0 | 0 | 0 | 0.0001 | NA | NA | NA | NA | NA | NA | NA | 0.3062 | 0.7203 | 0.3062 | 0.3062 | 0.1873 | 0.0000 | 0.1873 | NA | 0.7203 | 0.8893 | 0.3297 | 0.8893 | 0.8893 | 0.0001 | 0.0000 | 0.0001 | NA | 0.3297 | NA | NA | NA | NA | NA | NA | NA | NA | NA | NA | NA | NA | NA | NA | NA | NA | NA | NA |
| INT\_log\_Ratio\_SPECIES | MYSO | 0 | 0 | 0 | 0.0000 | NA | NA | NA | NA | NA | NA | NA | 0.5747 | 0.7006 | 0.5747 | 0.5747 | 0.9564 | 0.0490 | 0.9564 | NA | 0.7006 | 0.8194 | 0.4496 | 0.8194 | 0.8194 | 0.0011 | 0.0000 | 0.0011 | NA | 0.4496 | 0.7875 | 0.7807 | 0.7875 | 0.7875 | 0.7588 | 0.5078 | 0.7588 | NA | 0.7807 | NA | NA | NA | NA | NA | NA | NA | NA | NA |
| sq\_Ratio\_SPECIES | MYSO | 0 | 0 | 0 | 0.0000 | NA | NA | NA | NA | NA | NA | NA | 0.0931 | 0.1894 | 0.0931 | 0.0931 | 0.7117 | 0.0000 | 0.7117 | NA | 0.1894 | NA | NA | NA | NA | NA | NA | NA | NA | NA | NA | NA | NA | NA | NA | NA | NA | NA | NA | 0.3239 | 0.5191 | 0.3239 | 0.3239 | 0.8202 | 0.0000 | 0.8202 | NA | 0.5191 |
| INT\_sq\_Ratio\_SPECIES | MYSO | 0 | 0 | 0 | 0.0000 | NA | NA | NA | NA | NA | NA | NA | 0.1800 | 0.4170 | 0.1800 | 0.1800 | 0.5869 | 0.0000 | 0.5869 | NA | 0.4170 | NA | NA | NA | NA | NA | NA | NA | NA | NA | 0.9650 | 0.7380 | 0.9650 | 0.9650 | 0.1935 | 0.0000 | 0.1935 | NA | 0.7380 | 0.3477 | 0.3890 | 0.3477 | 0.3477 | 0.5070 | 0.5782 | 0.5070 | NA | 0.3890 |
| Ratio\_Bats | PESU | 0 | 0 | 0 | 0.0000 | NA | NA | NA | NA | NA | NA | NA | NA | NA | NA | NA | NA | NA | NA | NA | NA | NA | NA | NA | NA | NA | NA | NA | NA | NA | NA | NA | NA | NA | NA | NA | NA | NA | NA | NA | NA | NA | NA | NA | NA | NA | NA | NA |
| log\_Ratio\_Bats | PESU | 0 | 0 | 0 | 0.0000 | 0 | NA | NA | NA | NA | NA | NA | NA | NA | NA | NA | NA | NA | NA | NA | NA | NA | NA | NA | NA | NA | NA | NA | NA | NA | NA | NA | NA | NA | NA | NA | NA | NA | NA | NA | NA | NA | NA | NA | NA | NA | NA | NA |
| INT\_log\_Ratio\_Bats | PESU | 0 | 0 | 0 | 0.0012 | 0 | 0.2996 | NA | NA | NA | NA | NA | NA | NA | NA | NA | NA | NA | NA | NA | NA | NA | NA | NA | NA | NA | NA | NA | NA | NA | NA | NA | NA | NA | NA | NA | NA | NA | NA | NA | NA | NA | NA | NA | NA | NA | NA | NA |
| sq\_Ratio\_Bats | PESU | 0 | 0 | 0 | 0.0000 | NA | NA | 0 | NA | NA | NA | NA | NA | NA | NA | NA | NA | NA | NA | NA | NA | NA | NA | NA | NA | NA | NA | NA | NA | NA | NA | NA | NA | NA | NA | NA | NA | NA | NA | NA | NA | NA | NA | NA | NA | NA | NA | NA |
| INT\_sq\_Ratio\_Bats | PESU | 0 | 0 | 0 | 0.0000 | NA | 0.0000 | 0 | NA | NA | NA | NA | NA | NA | NA | NA | NA | NA | NA | NA | NA | NA | NA | NA | NA | NA | NA | NA | NA | NA | NA | NA | NA | NA | NA | NA | NA | NA | NA | NA | NA | NA | NA | NA | NA | NA | NA | NA |
| Ratio\_High/Low | PESU | 0 | 0 | 0 | 0.0000 | NA | NA | NA | 0.0000 | NA | NA | NA | NA | NA | NA | NA | NA | NA | NA | NA | NA | NA | NA | NA | NA | NA | NA | NA | NA | NA | NA | NA | NA | NA | NA | NA | NA | NA | NA | NA | NA | NA | NA | NA | NA | NA | NA | NA |
| log\_Ratio\_High/Low | PESU | 0 | 0 | 0 | 0.0048 | NA | NA | NA | 0.0000 | 0 | NA | NA | NA | NA | NA | NA | NA | NA | NA | NA | NA | NA | NA | NA | NA | NA | NA | NA | NA | NA | NA | NA | NA | NA | NA | NA | NA | NA | NA | NA | NA | NA | NA | NA | NA | NA | NA | NA |
| INT\_log\_Ratio\_High/Low | PESU | 0 | 0 | 0 | 0.0010 | NA | NA | NA | 0.0000 | 0 | 0.0247 | NA | NA | NA | NA | NA | NA | NA | NA | NA | NA | NA | NA | NA | NA | NA | NA | NA | NA | NA | NA | NA | NA | NA | NA | NA | NA | NA | NA | NA | NA | NA | NA | NA | NA | NA | NA | NA |
| sq\_Ratio\_High/Low | PESU | 0 | 0 | 0 | 0.0123 | NA | NA | NA | 0.0000 | NA | NA | 0 | NA | NA | NA | NA | NA | NA | NA | NA | NA | NA | NA | NA | NA | NA | NA | NA | NA | NA | NA | NA | NA | NA | NA | NA | NA | NA | NA | NA | NA | NA | NA | NA | NA | NA | NA | NA |
| INT\_sq\_Ratio\_High/Low | PESU | 0 | 0 | 0 | 0.0011 | NA | NA | NA | 0.0000 | NA | 0.0000 | 0 | NA | NA | NA | NA | NA | NA | NA | NA | NA | NA | NA | NA | NA | NA | NA | NA | NA | NA | NA | NA | NA | NA | NA | NA | NA | NA | NA | NA | NA | NA | NA | NA | NA | NA | NA | NA |
| Ratio\_SPECIES | PESU | 0 | 0 | 0 | 0.0000 | NA | NA | NA | NA | NA | NA | NA | 0.0711 | 0.5410 | 0.0711 | 0.0711 | 0.0874 | 0.2326 | 0.2326 | 0.2326 | NA | NA | NA | NA | NA | NA | NA | NA | NA | NA | NA | NA | NA | NA | NA | NA | NA | NA | NA | NA | NA | NA | NA | NA | NA | NA | NA | NA |
| log\_Ratio\_SPECIES | PESU | 0 | 0 | 0 | 0.0000 | NA | NA | NA | NA | NA | NA | NA | 0.2528 | 0.0000 | 0.2528 | 0.2528 | 0.5568 | 0.0549 | 0.0549 | 0.0549 | NA | 0.8664 | 0.0000 | 0.8664 | 0.8664 | 0.4723 | 0.0000 | 0.0000 | 0.0000 | NA | NA | NA | NA | NA | NA | NA | NA | NA | NA | NA | NA | NA | NA | NA | NA | NA | NA | NA |
| INT\_log\_Ratio\_SPECIES | PESU | 0 | 0 | 0 | 0.0001 | NA | NA | NA | NA | NA | NA | NA | 0.9948 | 0.0435 | 0.9948 | 0.9948 | 0.8076 | 0.2721 | 0.2721 | 0.2721 | NA | 0.9737 | 0.0000 | 0.9737 | 0.9737 | 0.4270 | 0.0000 | 0.0000 | 0.0000 | NA | 0.7546 | 0.4723 | 0.7546 | 0.7546 | 0.6904 | 0.5746 | 0.5746 | 0.5746 | NA | NA | NA | NA | NA | NA | NA | NA | NA | NA |
| sq\_Ratio\_SPECIES | PESU | 0 | 0 | 0 | 0.0000 | NA | NA | NA | NA | NA | NA | NA | 0.0417 | 0.4531 | 0.0417 | 0.0417 | 0.0705 | 0.5412 | 0.5412 | 0.5412 | NA | NA | NA | NA | NA | NA | NA | NA | NA | NA | NA | NA | NA | NA | NA | NA | NA | NA | NA | 0.2129 | 0.1723 | 0.2129 | 0.2129 | 0.3027 | 0.9283 | 0.9283 | 0.9283 | NA |
| INT\_sq\_Ratio\_SPECIES | PESU | 0 | 0 | 0 | 0.0000 | NA | NA | NA | NA | NA | NA | NA | 0.0652 | 0.0240 | 0.0652 | 0.0652 | 0.1325 | 0.7864 | 0.7864 | 0.7864 | NA | NA | NA | NA | NA | NA | NA | NA | NA | NA | 0.5630 | 0.0155 | 0.5630 | 0.5630 | 0.7428 | 0.3282 | 0.3282 | 0.3282 | NA | 0.4436 | 0.8025 | 0.4436 | 0.4436 | 0.4681 | 0.6073 | 0.6073 | 0.6073 | NA |

## MAE Boxplots

Boxplots for each model. Contains values for each of the nine
Examined Species. Black horizontal line shows median. Outliers defined
as datapoints > 1.5 IQR below Q1 or above
Q3.

## RMSE Boxplots

Boxplots for each model. Contains values for each of the nine
Examined Species. Black horizontal line shows median. Outliers defined
as datapoints > 1.5 IQR below Q1 or above
Q3.

## R2 Boxplots

Boxplots for each model. Contains values for each of the nine
Examined Species. Black horizontal line shows median. Outliers defined
as datapoints > 1.5 IQR below Q1 or above
Q3.

## *Ratio\_Bats*

### *Count and Species Ratio Plot*

See text for details on plot
formation.

### *Fort Drum Military Installation Case Study Plots*

Plots using species ratio derived from Nocera 2019 with varying total
audio file counts. Error bars show +/- 2 MAE. See text for details.

#### *Total Files = 75*

#### *Total Files = 175*

#### *Total Files = 275*

#### *Total Files = 475*

### *MLE Plots*

Each simulated night’s model predicted MLE and the actual MLE from
the program.

Histogram of errors for each simulated night’s modeled MLE. Model MLE
- Program MLE = Error.

## *log\_Ratio\_Bats*

### *Count and Species Ratio Plot*

See text for details on plot
formation.

### *Fort Drum Military Installation Case Study Plots*

Plots using species ratio derived from Nocera 2019 with varying total
audio file counts. Error bars show +/- 2 MAE. See text for details.

#### *Total Files = 75*

#### *Total Files = 175*

#### *Total Files = 275*

#### *Total Files = 475*

### *MLE Plots*

Each simulated night’s model predicted MLE and the actual MLE from
the program.

Histogram of errors for each simulated night’s modeled MLE. Model MLE
- Program MLE = Error.

## *INT\_log\_Ratio\_Bats*

### *Count and Species Ratio Plot*

See text for details on plot
formation.

### *Fort Drum Military Installation Case Study Plots*

Plots using species ratio derived from Nocera 2019 with varying total
audio file counts. Error bars show +/- 2 MAE. See text for details.

#### *Total Files = 75*

#### *Total Files = 175*

#### *Total Files = 275*

#### *Total Files = 475*

### *MLE Plots*

Each simulated night’s model predicted MLE and the actual MLE from
the program.

Histogram of errors for each simulated night’s modeled MLE. Model MLE
- Program MLE = Error.

## *sq\_Ratio\_Bats*

### *Count and Species Ratio Plot*

See text for details on plot
formation.

### *Fort Drum Military Installation Case Study Plots*

Plots using species ratio derived from Nocera 2019 with varying total
audio file counts. Error bars show +/- 2 MAE. See text for details.

#### *Total Files = 75*

#### *Total Files = 175*

#### *Total Files = 275*

#### *Total Files = 475*

### *MLE Plots*

Each simulated night’s model predicted MLE and the actual MLE from
the program.

Histogram of errors for each simulated night’s modeled MLE. Model MLE
- Program MLE = Error.

## *INT\_sq\_Ratio\_Bats*

### *Count and Species Ratio Plot*

See text for details on plot
formation.

### *Fort Drum Military Installation Case Study Plots*

Plots using species ratio derived from Nocera 2019 with varying total
audio file counts. Error bars show +/- 2 MAE. See text for details.

#### *Total Files = 75*

#### *Total Files = 175*

#### *Total Files = 275*

#### *Total Files = 475*

### *MLE Plots*

Each simulated night’s model predicted MLE and the actual MLE from
the program.

Histogram of errors for each simulated night’s modeled MLE. Model MLE
- Program MLE = Error.

## *Ratio\_High/Low*

### *Count and Species Ratio Plot*

See text for details on plot
formation.

### *Fort Drum Military Installation Case Study Plots*

Plots using species ratio derived from Nocera 2019 with varying total
audio file counts. Error bars show +/- 2 MAE. See text for details.

#### *Total Files = 75*

#### *Total Files = 175*

#### *Total Files = 275*

#### *Total Files = 475*

### *MLE Plots*

Each simulated night’s model predicted MLE and the actual MLE from
the program.

Histogram of errors for each simulated night’s modeled MLE. Model MLE
- Program MLE = Error.

## *log\_Ratio\_High/Low*

### *Count and Species Ratio Plot*

See text for details on plot
formation.

### *Fort Drum Military Installation Case Study Plots*

Plots using species ratio derived from Nocera 2019 with varying total
audio file counts. Error bars show +/- 2 MAE. See text for details.

#### *Total Files = 75*

#### *Total Files = 175*

#### *Total Files = 275*

#### *Total Files = 475*

### *MLE Plots*

Each simulated night’s model predicted MLE and the actual MLE from
the program.

Histogram of errors for each simulated night’s modeled MLE. Model MLE
- Program MLE = Error.

## *INT\_log\_Ratio\_High/Low*

### *Count and Species Ratio Plot*

See text for details on plot
formation.

### *Fort Drum Military Installation Case Study Plots*

Plots using species ratio derived from Nocera 2019 with varying total
audio file counts. Error bars show +/- 2 MAE. See text for details.

#### *Total Files = 75*

#### *Total Files = 175*

#### *Total Files = 275*

#### *Total Files = 475*

### *MLE Plots*

Each simulated night’s model predicted MLE and the actual MLE from
the program.

Histogram of errors for each simulated night’s modeled MLE. Model MLE
- Program MLE = Error.

## *sq\_Ratio\_High/Low*

### *Count and Species Ratio Plot*

See text for details on plot
formation.

### *Fort Drum Military Installation Case Study Plots*

Plots using species ratio derived from Nocera 2019 with varying total
audio file counts. Error bars show +/- 2 MAE. See text for details.

#### *Total Files = 75*

#### *Total Files = 175*

#### *Total Files = 275*

#### *Total Files = 475*

### *MLE Plots*

Each simulated night’s model predicted MLE and the actual MLE from
the program.

Histogram of errors for each simulated night’s modeled MLE. Model MLE
- Program MLE = Error.

## *INT\_sq\_Ratio\_High/Low*

### *Count and Species Ratio Plot*

See text for details on plot
formation.

### *Fort Drum Military Installation Case Study Plots*

Plots using species ratio derived from Nocera 2019 with varying total
audio file counts. Error bars show +/- 2 MAE. See text for details.

#### *Total Files = 75*

#### *Total Files = 175*

#### *Total Files = 275*

#### *Total Files = 475*

### *MLE Plots*

Each simulated night’s model predicted MLE and the actual MLE from
the program.

Histogram of errors for each simulated night’s modeled MLE. Model MLE
- Program MLE = Error.

## *Ratio\_SPECIES*

### *Count and Species Ratio Plot*

See text for details on plot
formation.

### *Fort Drum Military Installation Case Study Plots*

Plots using species ratio derived from Nocera 2019 with varying total
audio file counts. Error bars show +/- 2 MAE. See text for details.

#### *Total Files = 75*

#### *Total Files = 175*

#### *Total Files = 275*

#### *Total Files = 475*

### *MLE Plots*

Each simulated night’s model predicted MLE and the actual MLE from
the program.

Histogram of errors for each simulated night’s modeled MLE. Model MLE
- Program MLE = Error.

## *log\_Ratio\_SPECIES*

### *Count and Species Ratio Plot*

See text for details on plot
formation.

### *Fort Drum Military Installation Case Study Plots*

Plots using species ratio derived from Nocera 2019 with varying total
audio file counts. Error bars show +/- 2 MAE. See text for details.

#### *Total Files = 75*

#### *Total Files = 175*

#### *Total Files = 275*

#### *Total Files = 475*

### *MLE Plots*

Each simulated night’s model predicted MLE and the actual MLE from
the program.

Histogram of errors for each simulated night’s modeled MLE. Model MLE
- Program MLE = Error.

## *INT\_log\_Ratio\_SPECIES*

### *Count and Species Ratio Plot*

See text for details on plot
formation.

### *Fort Drum Military Installation Case Study Plots*

Plots using species ratio derived from Nocera 2019 with varying total
audio file counts. Error bars show +/- 2 MAE. See text for details.

#### *Total Files = 75*

#### *Total Files = 175*

#### *Total Files = 275*

#### *Total Files = 475*

### *MLE Plots*

Each simulated night’s model predicted MLE and the actual MLE from
the program.

Histogram of errors for each simulated night’s modeled MLE. Model MLE
- Program MLE = Error.

## *sq\_Ratio\_SPECIES*

### *Count and Species Ratio Plot*

See text for details on plot
formation.

### *Fort Drum Military Installation Case Study Plots*

Plots using species ratio derived from Nocera 2019 with varying total
audio file counts. Error bars show +/- 2 MAE. See text for details.

#### *Total Files = 75*

#### *Total Files = 175*

#### *Total Files = 275*

#### *Total Files = 475*

### *MLE Plots*

Each simulated night’s model predicted MLE and the actual MLE from
the program.

Histogram of errors for each simulated night’s modeled MLE. Model MLE
- Program MLE = Error.

## *INT\_sq\_Ratio\_SPECIES*

### *Count and Species Ratio Plot*

See text for details on plot
formation.

### *Fort Drum Military Installation Case Study Plots*

Plots using species ratio derived from Nocera 2019 with varying total
audio file counts. Error bars show +/- 2 MAE. See text for details.

#### *Total Files = 75*

#### *Total Files = 175*

#### *Total Files = 275*

#### *Total Files = 475*

### *MLE Plots*

Each simulated night’s model predicted MLE and the actual MLE from
the program.

Histogram of errors for each simulated night’s modeled MLE. Model MLE
- Program MLE = Error.
