# Supplementary material for: Maximum likelihood estimators are ineffective for acoustic detection of rare bat species
Source: PLoS One. 2025 Apr 1;20(4):e0320646. doi: 10.1371/journal.pone.0320646 (PMC11960983; doi:10.1371/journal.pone.0320646)
Supplement: S2 File — Contains metrics describing simulated nights and models tested for KPro. (HTML) [file pone.0320646.s012.html]

KPro MLE


Code 

- Show All Code
- Hide All Code

# KPro MLE

#### Bradley Hopp

#### 2025-02-25

# Subset Description

# Abbreviations

| Abbreviation | Scientific Name |
| --- | --- |
| EPFU | Eptesicus fuscus |
| LABO | Lasiurus borealis |
| LACI | Lasiurus cinereus |
| LANO | Lasionycteris noctivagans |
| MYLE | Myotis leibii |
| MYLU | Myotis lucifugus |
| MYSE | Myotis septentrionalis |
| MYSO | Myotis sodalis |
| PESU | Perimyotis subflavus |

# MLE v Count

Horizontal, dashed blue line reveals MLE = 0.05. Datapoints represent
simulated nights. Y-axis, ‘MLE’, is software MLE. X-axis, ‘Count’, is
the number of species files
present.

# MLE v Species Ratio

Horizontal, dashed blue line reveals MLE = 0.05. Datapoints represent
simulated nights. Y-axis, ‘MLE’, is software MLE. X-axis is Ratio\_Bats.
Different frames represent different file Counts.

### *EPFU*

### *LABO*

### *LACI*

### *LANO*

### *MYLE*

### *MYLU*

### *MYSE*

### *MYSO*

### *PESU*

# Models

## GLM Coefs

Adj R2 is R score but all model MLE’s > 1 are set to one and < 0
are set to 0.

Coefficient Estimates

| Vars | Species | (Intercept) | Ex\_Count | log\_Ex\_Count | Ratio\_Bats | log\_Ratio\_Bats | Ex\_Count\_Ratio\_Bats | sq\_Ratio\_Bats | Ratio\_High/Low | log\_Ratio\_High/Low | Ex\_Count\_Ratio\_High/Low | sq\_Ratio\_High/Low | Ratio\_EPFU | Ratio\_LABO | Ratio\_LACI | Ratio\_LANO | Ratio\_MYLE | Ratio\_MYLU | Ratio\_MYSE | Ratio\_MYSO | Ratio\_PESU | log\_Ratio\_EPFU | log\_Ratio\_LABO | log\_Ratio\_LACI | log\_Ratio\_LANO | log\_Ratio\_MYLE | log\_Ratio\_MYLU | log\_Ratio\_MYSE | log\_Ratio\_MYSO | log\_Ratio\_PESU | Ex\_Count\_Ratio\_EPFU | Ex\_Count\_Ratio\_LABO | Ex\_Count\_Ratio\_LACI | Ex\_Count\_Ratio\_LANO | Ex\_Count\_Ratio\_MYLE | Ex\_Count\_Ratio\_MYLU | Ex\_Count\_Ratio\_MYSE | Ex\_Count\_Ratio\_MYSO | Ex\_Count\_Ratio\_PESU | sq\_Ratio\_EPFU | sq\_Ratio\_LABO | sq\_Ratio\_LACI | sq\_Ratio\_LANO | sq\_Ratio\_MYLE | sq\_Ratio\_MYLU | sq\_Ratio\_MYSE | sq\_Ratio\_MYSO | sq\_Ratio\_PESU | Adj\_R2 | R2 | MAE | RMSE |
| --- | --- | --- | --- | --- | --- | --- | --- | --- | --- | --- | --- | --- | --- | --- | --- | --- | --- | --- | --- | --- | --- | --- | --- | --- | --- | --- | --- | --- | --- | --- | --- | --- | --- | --- | --- | --- | --- | --- | --- | --- | --- | --- | --- | --- | --- | --- | --- | --- | --- | --- | --- | --- |
| Ratio\_Bats | EPFU | 0.6570 | 0.0240 | -0.3653 | -0.3977 | NA | NA | NA | NA | NA | NA | NA | NA | NA | NA | NA | NA | NA | NA | NA | NA | NA | NA | NA | NA | NA | NA | NA | NA | NA | NA | NA | NA | NA | NA | NA | NA | NA | NA | NA | NA | NA | NA | NA | NA | NA | NA | NA | 0.6343 | 0.6230 | 0.0623 | 0.1261 |
| log\_Ratio\_Bats | EPFU | -0.6133 | 0.0169 | -0.2423 | 1.4582 | -0.3837 | NA | NA | NA | NA | NA | NA | NA | NA | NA | NA | NA | NA | NA | NA | NA | NA | NA | NA | NA | NA | NA | NA | NA | NA | NA | NA | NA | NA | NA | NA | NA | NA | NA | NA | NA | NA | NA | NA | NA | NA | NA | NA | 0.7582 | 0.7396 | 0.0518 | 0.1049 |
| INT\_log\_Ratio\_Bats | EPFU | -0.5732 | 0.0155 | -0.2390 | 1.2420 | -0.3745 | 0.0101 | NA | NA | NA | NA | NA | NA | NA | NA | NA | NA | NA | NA | NA | NA | NA | NA | NA | NA | NA | NA | NA | NA | NA | NA | NA | NA | NA | NA | NA | NA | NA | NA | NA | NA | NA | NA | NA | NA | NA | NA | NA | 0.7578 | 0.7398 | 0.0518 | 0.1049 |
| sq\_Ratio\_Bats | EPFU | 0.7749 | 0.0219 | -0.3114 | -2.9044 | NA | NA | 4.1689 | NA | NA | NA | NA | NA | NA | NA | NA | NA | NA | NA | NA | NA | NA | NA | NA | NA | NA | NA | NA | NA | NA | NA | NA | NA | NA | NA | NA | NA | NA | NA | NA | NA | NA | NA | NA | NA | NA | NA | NA | 0.6947 | 0.6666 | 0.0623 | 0.1187 |
| INT\_sq\_Ratio\_Bats | EPFU | 0.8126 | 0.0102 | -0.2796 | -3.4704 | NA | 0.0774 | 2.9201 | NA | NA | NA | NA | NA | NA | NA | NA | NA | NA | NA | NA | NA | NA | NA | NA | NA | NA | NA | NA | NA | NA | NA | NA | NA | NA | NA | NA | NA | NA | NA | NA | NA | NA | NA | NA | NA | NA | NA | NA | 0.7027 | 0.6763 | 0.0599 | 0.1171 |
| Ratio\_High/Low | EPFU | 0.9598 | 0.0259 | -0.3682 | 1.7857 | NA | NA | NA | -1.6903 | NA | NA | NA | NA | NA | NA | NA | NA | NA | NA | NA | NA | NA | NA | NA | NA | NA | NA | NA | NA | NA | NA | NA | NA | NA | NA | NA | NA | NA | NA | NA | NA | NA | NA | NA | NA | NA | NA | NA | 0.8599 | 0.7974 | 0.0571 | 0.0924 |
| log\_Ratio\_High/Low | EPFU | -0.3355 | 0.0193 | -0.2796 | 0.2899 | NA | NA | NA | 0.6431 | -0.4935 | NA | NA | NA | NA | NA | NA | NA | NA | NA | NA | NA | NA | NA | NA | NA | NA | NA | NA | NA | NA | NA | NA | NA | NA | NA | NA | NA | NA | NA | NA | NA | NA | NA | NA | NA | NA | NA | NA | 0.9161 | 0.8694 | 0.0401 | 0.0745 |
| INT\_log\_Ratio\_High/Low | EPFU | 0.2098 | -0.0006 | -0.2625 | 0.0985 | NA | NA | NA | -0.4522 | -0.3321 | 0.0538 | NA | NA | NA | NA | NA | NA | NA | NA | NA | NA | NA | NA | NA | NA | NA | NA | NA | NA | NA | NA | NA | NA | NA | NA | NA | NA | NA | NA | NA | NA | NA | NA | NA | NA | NA | NA | NA | 0.9198 | 0.8812 | 0.0386 | 0.0711 |
| sq\_Ratio\_High/Low | EPFU | 1.3312 | 0.0195 | -0.2535 | -1.5452 | NA | NA | NA | -3.9336 | NA | NA | 4.7447 | NA | NA | NA | NA | NA | NA | NA | NA | NA | NA | NA | NA | NA | NA | NA | NA | NA | NA | NA | NA | NA | NA | NA | NA | NA | NA | NA | NA | NA | NA | NA | NA | NA | NA | NA | NA | 0.9190 | 0.8897 | 0.0414 | 0.0683 |
| INT\_sq\_Ratio\_High/Low | EPFU | 1.3370 | 0.0061 | -0.2460 | -1.3246 | NA | NA | NA | -3.7009 | NA | 0.0358 | 3.7848 | NA | NA | NA | NA | NA | NA | NA | NA | NA | NA | NA | NA | NA | NA | NA | NA | NA | NA | NA | NA | NA | NA | NA | NA | NA | NA | NA | NA | NA | NA | NA | NA | NA | NA | NA | NA | 0.9219 | 0.8945 | 0.0399 | 0.0668 |
| Ratio\_SPECIES | EPFU | 0.6665 | 0.0227 | -0.3458 | -1.1167 | NA | NA | NA | NA | NA | NA | NA | NA | 0.0059 | 0.0019 | -0.0023 | 0.0060 | 0.0060 | 0.0060 | 0.0060 | 0.0060 | NA | NA | NA | NA | NA | NA | NA | NA | NA | NA | NA | NA | NA | NA | NA | NA | NA | NA | NA | NA | NA | NA | NA | NA | NA | NA | NA | 0.6486 | 0.6330 | 0.0638 | 0.1245 |
| log\_Ratio\_SPECIES | EPFU | 0.3953 | 0.0235 | -0.3330 | 1.0636 | NA | NA | NA | NA | NA | NA | NA | NA | -0.0073 | 0.0244 | 0.0477 | -0.0077 | -0.0078 | -0.0077 | -0.0077 | -0.0078 | NA | 0.0090 | -0.1372 | -0.2496 | 0.0110 | 0.0111 | 0.0110 | 0.0110 | 0.0111 | NA | NA | NA | NA | NA | NA | NA | NA | NA | NA | NA | NA | NA | NA | NA | NA | NA | NA | 0.9101 | 0.8526 | 0.0492 | 0.0794 |
| INT\_log\_Ratio\_SPECIES | EPFU | 0.3827 | 0.0243 | -0.3340 | 1.0359 | NA | NA | NA | NA | NA | NA | NA | NA | -0.0090 | 0.0375 | 0.0610 | -0.0097 | -0.0098 | -0.0097 | -0.0097 | -0.0098 | NA | 0.0099 | -0.1428 | -0.2552 | 0.0120 | 0.0121 | 0.0120 | 0.0120 | 0.0121 | NA | 0.0001 | -0.0006 | -0.0006 | 0.0001 | 0.0001 | 0.0001 | 0.0001 | 0.0001 | NA | NA | NA | NA | NA | NA | NA | NA | NA | 0.9116 | 0.8533 | 0.0494 | 0.0794 |
| sq\_Ratio\_SPECIES | EPFU | 0.6650 | 0.0226 | -0.3446 | -1.1791 | NA | NA | NA | NA | NA | NA | NA | NA | 0.0145 | -0.0106 | -0.0365 | 0.0148 | 0.0148 | 0.0148 | 0.0148 | 0.0148 | NA | NA | NA | NA | NA | NA | NA | NA | NA | NA | NA | NA | NA | NA | NA | NA | NA | NA | NA | -0.0005 | 0.0008 | 0.0021 | -0.0005 | -0.0005 | -5e-04 | -5e-04 | -5e-04 | 0.6642 | 0.6424 | 0.0656 | 0.1231 |
| INT\_sq\_Ratio\_SPECIES | EPFU | 0.7069 | 0.0159 | -0.3250 | -1.3261 | NA | NA | NA | NA | NA | NA | NA | NA | 0.0207 | -0.0378 | -0.0921 | 0.0215 | 0.0215 | 0.0215 | 0.0215 | 0.0215 | NA | NA | NA | NA | NA | NA | NA | NA | NA | NA | -0.0004 | 0.0023 | 0.0045 | -0.0004 | -0.0004 | -0.0004 | -0.0004 | -0.0004 | NA | -0.0004 | -0.0002 | 0.0003 | -0.0004 | -0.0004 | -4e-04 | -4e-04 | -4e-04 | 0.6973 | 0.6638 | 0.0639 | 0.1200 |
| Ratio\_Bats | LABO | 0.2836 | 0.0151 | -0.1897 | -0.1522 | NA | NA | NA | NA | NA | NA | NA | NA | NA | NA | NA | NA | NA | NA | NA | NA | NA | NA | NA | NA | NA | NA | NA | NA | NA | NA | NA | NA | NA | NA | NA | NA | NA | NA | NA | NA | NA | NA | NA | NA | NA | NA | NA | 0.4605 | 0.4328 | 0.0368 | 0.0815 |
| log\_Ratio\_Bats | LABO | -0.5474 | 0.0105 | -0.1093 | 1.0618 | -0.2510 | NA | NA | NA | NA | NA | NA | NA | NA | NA | NA | NA | NA | NA | NA | NA | NA | NA | NA | NA | NA | NA | NA | NA | NA | NA | NA | NA | NA | NA | NA | NA | NA | NA | NA | NA | NA | NA | NA | NA | NA | NA | NA | 0.6465 | 0.6128 | 0.0335 | 0.0675 |
| INT\_log\_Ratio\_Bats | LABO | -0.6905 | 0.0156 | -0.1210 | 1.8340 | -0.2840 | -0.0360 | NA | NA | NA | NA | NA | NA | NA | NA | NA | NA | NA | NA | NA | NA | NA | NA | NA | NA | NA | NA | NA | NA | NA | NA | NA | NA | NA | NA | NA | NA | NA | NA | NA | NA | NA | NA | NA | NA | NA | NA | NA | 0.6554 | 0.6203 | 0.0336 | 0.0668 |
| sq\_Ratio\_Bats | LABO | 0.3336 | 0.0142 | -0.1669 | -1.2167 | NA | NA | 1.7703 | NA | NA | NA | NA | NA | NA | NA | NA | NA | NA | NA | NA | NA | NA | NA | NA | NA | NA | NA | NA | NA | NA | NA | NA | NA | NA | NA | NA | NA | NA | NA | NA | NA | NA | NA | NA | NA | NA | NA | NA | 0.5041 | 0.4612 | 0.0376 | 0.0795 |
| INT\_sq\_Ratio\_Bats | LABO | 0.3493 | 0.0094 | -0.1537 | -1.4517 | NA | 0.0322 | 1.2516 | NA | NA | NA | NA | NA | NA | NA | NA | NA | NA | NA | NA | NA | NA | NA | NA | NA | NA | NA | NA | NA | NA | NA | NA | NA | NA | NA | NA | NA | NA | NA | NA | NA | NA | NA | NA | NA | NA | NA | NA | 0.5109 | 0.4673 | 0.0369 | 0.0791 |
| Ratio\_High/Low | LABO | 0.3303 | 0.0156 | -0.1912 | 1.3766 | NA | NA | NA | -1.3139 | NA | NA | NA | NA | NA | NA | NA | NA | NA | NA | NA | NA | NA | NA | NA | NA | NA | NA | NA | NA | NA | NA | NA | NA | NA | NA | NA | NA | NA | NA | NA | NA | NA | NA | NA | NA | NA | NA | NA | 0.5183 | 0.4784 | 0.0374 | 0.0782 |
| log\_Ratio\_High/Low | LABO | -0.6893 | 0.0098 | -0.1013 | -1.1065 | NA | NA | NA | 2.0243 | -0.3266 | NA | NA | NA | NA | NA | NA | NA | NA | NA | NA | NA | NA | NA | NA | NA | NA | NA | NA | NA | NA | NA | NA | NA | NA | NA | NA | NA | NA | NA | NA | NA | NA | NA | NA | NA | NA | NA | NA | 0.6957 | 0.6689 | 0.0306 | 0.0624 |
| INT\_log\_Ratio\_High/Low | LABO | -0.8046 | 0.0145 | -0.1103 | -0.9400 | NA | NA | NA | 2.3690 | -0.3550 | -0.0229 | NA | NA | NA | NA | NA | NA | NA | NA | NA | NA | NA | NA | NA | NA | NA | NA | NA | NA | NA | NA | NA | NA | NA | NA | NA | NA | NA | NA | NA | NA | NA | NA | NA | NA | NA | NA | NA | 0.7005 | 0.6733 | 0.0307 | 0.0620 |
| sq\_Ratio\_High/Low | LABO | 0.3920 | 0.0142 | -0.1602 | 0.0191 | NA | NA | NA | -1.4373 | NA | NA | 1.7582 | NA | NA | NA | NA | NA | NA | NA | NA | NA | NA | NA | NA | NA | NA | NA | NA | NA | NA | NA | NA | NA | NA | NA | NA | NA | NA | NA | NA | NA | NA | NA | NA | NA | NA | NA | NA | 0.5628 | 0.5076 | 0.0379 | 0.0761 |
| INT\_sq\_Ratio\_High/Low | LABO | 0.4112 | 0.0076 | -0.1461 | -0.1282 | NA | NA | NA | -1.4775 | NA | 0.0306 | 1.2882 | NA | NA | NA | NA | NA | NA | NA | NA | NA | NA | NA | NA | NA | NA | NA | NA | NA | NA | NA | NA | NA | NA | NA | NA | NA | NA | NA | NA | NA | NA | NA | NA | NA | NA | NA | NA | 0.5688 | 0.5160 | 0.0367 | 0.0755 |
| Ratio\_SPECIES | LABO | 0.2870 | 0.0147 | -0.1829 | -0.4060 | NA | NA | NA | NA | NA | NA | NA | 0.0022 | NA | 0.0022 | 0.0022 | 0.0022 | -0.0014 | 0.0022 | 0.0024 | 0.0005 | NA | NA | NA | NA | NA | NA | NA | NA | NA | NA | NA | NA | NA | NA | NA | NA | NA | NA | NA | NA | NA | NA | NA | NA | NA | NA | NA | 0.4684 | 0.4378 | 0.0371 | 0.0812 |
| log\_Ratio\_SPECIES | LABO | 0.1078 | 0.0152 | -0.1745 | 1.0340 | NA | NA | NA | NA | NA | NA | NA | -0.0048 | NA | -0.0048 | -0.0048 | -0.0051 | 0.0271 | -0.0054 | -0.0067 | 0.0109 | -0.0012 | NA | -0.0012 | -0.0012 | 0.0000 | -0.1464 | 0.0016 | 0.0076 | -0.0722 | NA | NA | NA | NA | NA | NA | NA | NA | NA | NA | NA | NA | NA | NA | NA | NA | NA | NA | 0.8086 | 0.7265 | 0.0360 | 0.0571 |
| INT\_log\_Ratio\_SPECIES | LABO | 0.0790 | 0.0172 | -0.1766 | 0.9705 | NA | NA | NA | NA | NA | NA | NA | -0.0041 | NA | -0.0041 | -0.0041 | -0.0047 | 0.0471 | -0.0053 | -0.0075 | 0.0229 | -0.0012 | NA | -0.0012 | -0.0012 | 0.0002 | -0.1548 | 0.0018 | 0.0082 | -0.0771 | 0.0000 | NA | 0.0000 | 0.0000 | 0.0000 | -0.0009 | 0.0000 | 0.0000 | -0.0005 | NA | NA | NA | NA | NA | NA | NA | NA | NA | 0.8148 | 0.7328 | 0.0365 | 0.0565 |
| sq\_Ratio\_SPECIES | LABO | 0.2873 | 0.0147 | -0.1832 | -0.3924 | NA | NA | NA | NA | NA | NA | NA | 0.0053 | NA | 0.0053 | 0.0053 | 0.0054 | -0.0173 | 0.0057 | 0.0065 | -0.0050 | NA | NA | NA | NA | NA | NA | NA | NA | NA | NA | NA | NA | NA | NA | NA | NA | NA | NA | -0.0002 | NA | -0.0002 | -0.0002 | -0.0002 | 0.0009 | -2e-04 | -3e-04 | 3e-04 | 0.4809 | 0.4445 | 0.0377 | 0.0808 |
| INT\_sq\_Ratio\_SPECIES | LABO | 0.3022 | 0.0123 | -0.1762 | -0.4449 | NA | NA | NA | NA | NA | NA | NA | 0.0089 | NA | 0.0089 | 0.0088 | 0.0093 | -0.0455 | 0.0098 | 0.0119 | -0.0163 | NA | NA | NA | NA | NA | NA | NA | NA | NA | -0.0002 | NA | -0.0002 | -0.0002 | -0.0003 | 0.0023 | -0.0003 | -0.0004 | 0.0009 | -0.0001 | NA | -0.0001 | -0.0001 | -0.0001 | 0.0001 | -1e-04 | -1e-04 | -1e-04 | 0.5108 | 0.4599 | 0.0375 | 0.0799 |
| Ratio\_Bats | LACI | 0.4512 | 0.0212 | -0.2825 | -0.2602 | NA | NA | NA | NA | NA | NA | NA | NA | NA | NA | NA | NA | NA | NA | NA | NA | NA | NA | NA | NA | NA | NA | NA | NA | NA | NA | NA | NA | NA | NA | NA | NA | NA | NA | NA | NA | NA | NA | NA | NA | NA | NA | NA | 0.5552 | 0.5343 | 0.0513 | 0.1046 |
| log\_Ratio\_Bats | LACI | -0.6556 | 0.0151 | -0.1753 | 1.3567 | -0.3343 | NA | NA | NA | NA | NA | NA | NA | NA | NA | NA | NA | NA | NA | NA | NA | NA | NA | NA | NA | NA | NA | NA | NA | NA | NA | NA | NA | NA | NA | NA | NA | NA | NA | NA | NA | NA | NA | NA | NA | NA | NA | NA | 0.7172 | 0.6933 | 0.0438 | 0.0850 |
| INT\_log\_Ratio\_Bats | LACI | -0.7449 | 0.0183 | -0.1827 | 1.8386 | -0.3549 | -0.0225 | NA | NA | NA | NA | NA | NA | NA | NA | NA | NA | NA | NA | NA | NA | NA | NA | NA | NA | NA | NA | NA | NA | NA | NA | NA | NA | NA | NA | NA | NA | NA | NA | NA | NA | NA | NA | NA | NA | NA | NA | NA | 0.7195 | 0.6947 | 0.0439 | 0.0848 |
| sq\_Ratio\_Bats | LACI | 0.5333 | 0.0197 | -0.2450 | -2.0054 | NA | NA | 2.9024 | NA | NA | NA | NA | NA | NA | NA | NA | NA | NA | NA | NA | NA | NA | NA | NA | NA | NA | NA | NA | NA | NA | NA | NA | NA | NA | NA | NA | NA | NA | NA | NA | NA | NA | NA | NA | NA | NA | NA | NA | 0.6112 | 0.5722 | 0.0520 | 0.1004 |
| INT\_sq\_Ratio\_Bats | LACI | 0.5598 | 0.0115 | -0.2226 | -2.4044 | NA | 0.0546 | 2.0219 | NA | NA | NA | NA | NA | NA | NA | NA | NA | NA | NA | NA | NA | NA | NA | NA | NA | NA | NA | NA | NA | NA | NA | NA | NA | NA | NA | NA | NA | NA | NA | NA | NA | NA | NA | NA | NA | NA | NA | NA | 0.6201 | 0.5809 | 0.0505 | 0.0995 |
| Ratio\_High/Low | LACI | 0.6812 | 0.0227 | -0.2847 | 1.3984 | NA | NA | NA | -1.2840 | NA | NA | NA | NA | NA | NA | NA | NA | NA | NA | NA | NA | NA | NA | NA | NA | NA | NA | NA | NA | NA | NA | NA | NA | NA | NA | NA | NA | NA | NA | NA | NA | NA | NA | NA | NA | NA | NA | NA | 0.8032 | 0.7151 | 0.0519 | 0.0819 |
| log\_Ratio\_High/Low | LACI | -0.8562 | 0.0148 | -0.1796 | -0.3771 | NA | NA | NA | 1.4857 | -0.5858 | NA | NA | NA | NA | NA | NA | NA | NA | NA | NA | NA | NA | NA | NA | NA | NA | NA | NA | NA | NA | NA | NA | NA | NA | NA | NA | NA | NA | NA | NA | NA | NA | NA | NA | NA | NA | NA | NA | 0.9315 | 0.8972 | 0.0305 | 0.0493 |
| INT\_log\_Ratio\_High/Low | LACI | -0.7785 | 0.0120 | -0.1772 | -0.4044 | NA | NA | NA | 1.3296 | -0.5628 | 0.0077 | NA | NA | NA | NA | NA | NA | NA | NA | NA | NA | NA | NA | NA | NA | NA | NA | NA | NA | NA | NA | NA | NA | NA | NA | NA | NA | NA | NA | NA | NA | NA | NA | NA | NA | NA | NA | NA | 0.9308 | 0.8976 | 0.0307 | 0.0493 |
| sq\_Ratio\_High/Low | LACI | 0.9971 | 0.0172 | -0.1872 | -1.4346 | NA | NA | NA | -3.1920 | NA | NA | 4.0355 | NA | NA | NA | NA | NA | NA | NA | NA | NA | NA | NA | NA | NA | NA | NA | NA | NA | NA | NA | NA | NA | NA | NA | NA | NA | NA | NA | NA | NA | NA | NA | NA | NA | NA | NA | NA | 0.8784 | 0.8349 | 0.0414 | 0.0624 |
| INT\_sq\_Ratio\_High/Low | LACI | 1.0017 | 0.0066 | -0.1812 | -1.2598 | NA | NA | NA | -3.0075 | NA | 0.0284 | 3.2745 | NA | NA | NA | NA | NA | NA | NA | NA | NA | NA | NA | NA | NA | NA | NA | NA | NA | NA | NA | NA | NA | NA | NA | NA | NA | NA | NA | NA | NA | NA | NA | NA | NA | NA | NA | NA | 0.8814 | 0.8404 | 0.0401 | 0.0614 |
| Ratio\_SPECIES | LACI | 0.4574 | 0.0203 | -0.2697 | -0.7320 | NA | NA | NA | NA | NA | NA | NA | -0.0013 | 0.0040 | NA | 0.0005 | 0.0040 | 0.0040 | 0.0040 | 0.0040 | 0.0040 | NA | NA | NA | NA | NA | NA | NA | NA | NA | NA | NA | NA | NA | NA | NA | NA | NA | NA | NA | NA | NA | NA | NA | NA | NA | NA | NA | 0.5672 | 0.5421 | 0.0520 | 0.1038 |
| log\_Ratio\_SPECIES | LACI | 0.2244 | 0.0210 | -0.2588 | 1.1414 | NA | NA | NA | NA | NA | NA | NA | 0.0355 | -0.0072 | NA | 0.0229 | -0.0072 | -0.0072 | -0.0072 | -0.0072 | -0.0072 | -0.1893 | 0.0072 | NA | -0.1303 | 0.0070 | 0.0070 | 0.0071 | 0.0071 | 0.0071 | NA | NA | NA | NA | NA | NA | NA | NA | NA | NA | NA | NA | NA | NA | NA | NA | NA | NA | 0.8777 | 0.8105 | 0.0457 | 0.0673 |
| INT\_log\_Ratio\_SPECIES | LACI | 0.1952 | 0.0230 | -0.2609 | 1.0770 | NA | NA | NA | NA | NA | NA | NA | 0.0558 | -0.0077 | NA | 0.0399 | -0.0077 | -0.0077 | -0.0077 | -0.0077 | -0.0077 | -0.1978 | 0.0077 | NA | -0.1374 | 0.0076 | 0.0076 | 0.0076 | 0.0076 | 0.0076 | -0.0009 | 0.0000 | NA | -0.0008 | 0.0000 | 0.0000 | 0.0000 | 0.0000 | 0.0000 | NA | NA | NA | NA | NA | NA | NA | NA | NA | 0.8819 | 0.8141 | 0.0462 | 0.0667 |
| sq\_Ratio\_SPECIES | LACI | 0.4557 | 0.0202 | -0.2683 | -0.8056 | NA | NA | NA | NA | NA | NA | NA | -0.0225 | 0.0107 | NA | -0.0112 | 0.0107 | 0.0107 | 0.0107 | 0.0107 | 0.0107 | NA | NA | NA | NA | NA | NA | NA | NA | NA | NA | NA | NA | NA | NA | NA | NA | NA | NA | 0.0013 | -0.0004 | NA | 0.0007 | -0.0004 | -0.0004 | -4e-04 | -4e-04 | -4e-04 | 0.5811 | 0.5502 | 0.0531 | 0.1030 |
| INT\_sq\_Ratio\_SPECIES | LACI | 0.4816 | 0.0161 | -0.2561 | -0.8967 | NA | NA | NA | NA | NA | NA | NA | -0.0612 | 0.0168 | NA | -0.0355 | 0.0167 | 0.0167 | 0.0167 | 0.0167 | 0.0167 | NA | NA | NA | NA | NA | NA | NA | NA | NA | 0.0031 | -0.0004 | NA | 0.0020 | -0.0004 | -0.0004 | -0.0004 | -0.0004 | -0.0004 | 0.0000 | -0.0003 | NA | -0.0001 | -0.0003 | -0.0003 | -3e-04 | -3e-04 | -3e-04 | 0.6146 | 0.5690 | 0.0525 | 0.1013 |
| Ratio\_Bats | LANO | 0.9835 | 0.0057 | -0.3338 | -0.6908 | NA | NA | NA | NA | NA | NA | NA | NA | NA | NA | NA | NA | NA | NA | NA | NA | NA | NA | NA | NA | NA | NA | NA | NA | NA | NA | NA | NA | NA | NA | NA | NA | NA | NA | NA | NA | NA | NA | NA | NA | NA | NA | NA | 0.7391 | 0.7279 | 0.0934 | 0.1541 |
| log\_Ratio\_Bats | LANO | -0.1290 | -0.0005 | -0.2260 | 0.9346 | -0.3361 | NA | NA | NA | NA | NA | NA | NA | NA | NA | NA | NA | NA | NA | NA | NA | NA | NA | NA | NA | NA | NA | NA | NA | NA | NA | NA | NA | NA | NA | NA | NA | NA | NA | NA | NA | NA | NA | NA | NA | NA | NA | NA | 0.7942 | 0.7711 | 0.0867 | 0.1415 |
| INT\_log\_Ratio\_Bats | LANO | 0.2203 | -0.0130 | -0.1975 | -0.9498 | -0.2556 | 0.0879 | NA | NA | NA | NA | NA | NA | NA | NA | NA | NA | NA | NA | NA | NA | NA | NA | NA | NA | NA | NA | NA | NA | NA | NA | NA | NA | NA | NA | NA | NA | NA | NA | NA | NA | NA | NA | NA | NA | NA | NA | NA | 0.7963 | 0.7771 | 0.0873 | 0.1396 |
| sq\_Ratio\_Bats | LANO | 1.1427 | 0.0028 | -0.2610 | -4.0753 | NA | NA | 5.6287 | NA | NA | NA | NA | NA | NA | NA | NA | NA | NA | NA | NA | NA | NA | NA | NA | NA | NA | NA | NA | NA | NA | NA | NA | NA | NA | NA | NA | NA | NA | NA | NA | NA | NA | NA | NA | NA | NA | NA | NA | 0.7877 | 0.7662 | 0.0896 | 0.1430 |
| INT\_sq\_Ratio\_Bats | LANO | 1.1900 | -0.0118 | -0.2212 | -4.7850 | NA | 0.0971 | 4.0626 | NA | NA | NA | NA | NA | NA | NA | NA | NA | NA | NA | NA | NA | NA | NA | NA | NA | NA | NA | NA | NA | NA | NA | NA | NA | NA | NA | NA | NA | NA | NA | NA | NA | NA | NA | NA | NA | NA | NA | NA | 0.7900 | 0.7736 | 0.0894 | 0.1408 |
| Ratio\_High/Low | LANO | 1.3569 | 0.0081 | -0.3374 | 2.0016 | NA | NA | NA | -2.0844 | NA | NA | NA | NA | NA | NA | NA | NA | NA | NA | NA | NA | NA | NA | NA | NA | NA | NA | NA | NA | NA | NA | NA | NA | NA | NA | NA | NA | NA | NA | NA | NA | NA | NA | NA | NA | NA | NA | NA | 0.9116 | 0.8559 | 0.0749 | 0.1122 |
| log\_Ratio\_High/Low | LANO | 1.2250 | 0.0074 | -0.3284 | 1.8492 | NA | NA | NA | -1.8467 | -0.0503 | NA | NA | NA | NA | NA | NA | NA | NA | NA | NA | NA | NA | NA | NA | NA | NA | NA | NA | NA | NA | NA | NA | NA | NA | NA | NA | NA | NA | NA | NA | NA | NA | NA | NA | NA | NA | NA | NA | 0.9123 | 0.8563 | 0.0741 | 0.1122 |
| INT\_log\_Ratio\_High/Low | LANO | 2.4038 | -0.0357 | -0.2914 | 1.4354 | NA | NA | NA | -4.2144 | 0.2987 | 0.1163 | NA | NA | NA | NA | NA | NA | NA | NA | NA | NA | NA | NA | NA | NA | NA | NA | NA | NA | NA | NA | NA | NA | NA | NA | NA | NA | NA | NA | NA | NA | NA | NA | NA | NA | NA | NA | NA | 0.9226 | 0.8828 | 0.0648 | 0.1014 |
| sq\_Ratio\_High/Low | LANO | 1.6243 | 0.0034 | -0.2548 | -0.3965 | NA | NA | NA | -3.6994 | NA | NA | 3.4159 | NA | NA | NA | NA | NA | NA | NA | NA | NA | NA | NA | NA | NA | NA | NA | NA | NA | NA | NA | NA | NA | NA | NA | NA | NA | NA | NA | NA | NA | NA | NA | NA | NA | NA | NA | NA | 0.9341 | 0.8790 | 0.0648 | 0.1030 |
| INT\_sq\_Ratio\_High/Low | LANO | 1.6305 | -0.0107 | -0.2469 | -0.1641 | NA | NA | NA | -3.4543 | NA | 0.0377 | 2.4049 | NA | NA | NA | NA | NA | NA | NA | NA | NA | NA | NA | NA | NA | NA | NA | NA | NA | NA | NA | NA | NA | NA | NA | NA | NA | NA | NA | NA | NA | NA | NA | NA | NA | NA | NA | NA | 0.9329 | 0.8816 | 0.0637 | 0.1019 |
| Ratio\_SPECIES | LANO | 0.9970 | 0.0038 | -0.3064 | -1.7048 | NA | NA | NA | NA | NA | NA | NA | -0.0029 | 0.0091 | -0.0022 | NA | 0.0091 | 0.0091 | 0.0091 | 0.0091 | 0.0091 | NA | NA | NA | NA | NA | NA | NA | NA | NA | NA | NA | NA | NA | NA | NA | NA | NA | NA | NA | NA | NA | NA | NA | NA | NA | NA | NA | 0.7522 | 0.7385 | 0.0946 | 0.1512 |
| log\_Ratio\_SPECIES | LANO | 0.7468 | 0.0045 | -0.2946 | 0.3064 | NA | NA | NA | NA | NA | NA | NA | 0.0397 | -0.0061 | 0.0376 | NA | -0.0060 | -0.0060 | -0.0060 | -0.0060 | -0.0060 | -0.2155 | 0.0205 | -0.2038 | NA | 0.0201 | 0.0201 | 0.0203 | 0.0203 | 0.0203 | NA | NA | NA | NA | NA | NA | NA | NA | NA | NA | NA | NA | NA | NA | NA | NA | NA | NA | 0.9030 | 0.8464 | 0.0730 | 0.1164 |
| INT\_log\_Ratio\_SPECIES | LANO | 0.8053 | 0.0005 | -0.2902 | 0.4355 | NA | NA | NA | NA | NA | NA | NA | 0.0195 | -0.0111 | 0.0192 | NA | -0.0111 | -0.0109 | -0.0110 | -0.0109 | -0.0109 | -0.2073 | 0.0221 | -0.1965 | NA | 0.0217 | 0.0216 | 0.0218 | 0.0218 | 0.0218 | 0.0009 | 0.0002 | 0.0008 | NA | 0.0002 | 0.0002 | 0.0002 | 0.0002 | 0.0002 | NA | NA | NA | NA | NA | NA | NA | NA | NA | 0.9011 | 0.8491 | 0.0728 | 0.1157 |
| sq\_Ratio\_SPECIES | LANO | 1.0056 | 0.0044 | -0.3139 | -1.3347 | NA | NA | NA | NA | NA | NA | NA | -0.0484 | 0.0176 | -0.0442 | NA | 0.0174 | 0.0175 | 0.0175 | 0.0176 | 0.0176 | NA | NA | NA | NA | NA | NA | NA | NA | NA | NA | NA | NA | NA | NA | NA | NA | NA | NA | 0.0026 | -0.0006 | 0.0024 | NA | -0.0006 | -0.0006 | -6e-04 | -6e-04 | -6e-04 | 0.7694 | 0.7495 | 0.0941 | 0.1483 |
| INT\_sq\_Ratio\_SPECIES | LANO | 1.0751 | -0.0068 | -0.2814 | -1.5786 | NA | NA | NA | NA | NA | NA | NA | -0.1047 | 0.0239 | -0.0974 | NA | 0.0237 | 0.0238 | 0.0238 | 0.0239 | 0.0239 | NA | NA | NA | NA | NA | NA | NA | NA | NA | 0.0046 | -0.0003 | 0.0044 | NA | -0.0003 | -0.0003 | -0.0003 | -0.0003 | -0.0003 | 0.0007 | -0.0006 | 0.0006 | NA | -0.0006 | -0.0006 | -6e-04 | -6e-04 | -6e-04 | 0.7915 | 0.7706 | 0.0908 | 0.1428 |
| Ratio\_Bats | MYLE | 0.3789 | 0.0192 | -0.2465 | -0.2107 | NA | NA | NA | NA | NA | NA | NA | NA | NA | NA | NA | NA | NA | NA | NA | NA | NA | NA | NA | NA | NA | NA | NA | NA | NA | NA | NA | NA | NA | NA | NA | NA | NA | NA | NA | NA | NA | NA | NA | NA | NA | NA | NA | 0.5321 | 0.5059 | 0.0454 | 0.0935 |
| log\_Ratio\_Bats | MYLE | -0.6226 | 0.0136 | -0.1495 | 1.2525 | -0.3025 | NA | NA | NA | NA | NA | NA | NA | NA | NA | NA | NA | NA | NA | NA | NA | NA | NA | NA | NA | NA | NA | NA | NA | NA | NA | NA | NA | NA | NA | NA | NA | NA | NA | NA | NA | NA | NA | NA | NA | NA | NA | NA | 0.7090 | 0.6789 | 0.0395 | 0.0755 |
| INT\_log\_Ratio\_Bats | MYLE | -0.7454 | 0.0180 | -0.1596 | 1.9147 | -0.3308 | -0.0309 | NA | NA | NA | NA | NA | NA | NA | NA | NA | NA | NA | NA | NA | NA | NA | NA | NA | NA | NA | NA | NA | NA | NA | NA | NA | NA | NA | NA | NA | NA | NA | NA | NA | NA | NA | NA | NA | NA | NA | NA | NA | 0.7133 | 0.6826 | 0.0396 | 0.0750 |
| sq\_Ratio\_Bats | MYLE | 0.4468 | 0.0180 | -0.2155 | -1.6540 | NA | NA | 2.4003 | NA | NA | NA | NA | NA | NA | NA | NA | NA | NA | NA | NA | NA | NA | NA | NA | NA | NA | NA | NA | NA | NA | NA | NA | NA | NA | NA | NA | NA | NA | NA | NA | NA | NA | NA | NA | NA | NA | NA | NA | 0.5843 | 0.5404 | 0.0462 | 0.0903 |
| INT\_sq\_Ratio\_Bats | MYLE | 0.4685 | 0.0112 | -0.1972 | -1.9797 | NA | 0.0445 | 1.6816 | NA | NA | NA | NA | NA | NA | NA | NA | NA | NA | NA | NA | NA | NA | NA | NA | NA | NA | NA | NA | NA | NA | NA | NA | NA | NA | NA | NA | NA | NA | NA | NA | NA | NA | NA | NA | NA | NA | NA | NA | 0.5926 | 0.5481 | 0.0451 | 0.0896 |
| Ratio\_High/Low | MYLE | 0.4436 | 0.0198 | -0.2486 | 1.9041 | NA | NA | NA | -1.8175 | NA | NA | NA | NA | NA | NA | NA | NA | NA | NA | NA | NA | NA | NA | NA | NA | NA | NA | NA | NA | NA | NA | NA | NA | NA | NA | NA | NA | NA | NA | NA | NA | NA | NA | NA | NA | NA | NA | NA | 0.6040 | 0.5636 | 0.0458 | 0.0879 |
| log\_Ratio\_High/Low | MYLE | -0.7572 | 0.0130 | -0.1426 | -1.0202 | NA | NA | NA | 2.1137 | -0.3847 | NA | NA | NA | NA | NA | NA | NA | NA | NA | NA | NA | NA | NA | NA | NA | NA | NA | NA | NA | NA | NA | NA | NA | NA | NA | NA | NA | NA | NA | NA | NA | NA | NA | NA | NA | NA | NA | NA | 0.7641 | 0.7384 | 0.0358 | 0.0681 |
| INT\_log\_Ratio\_High/Low | MYLE | -0.8373 | 0.0163 | -0.1489 | -0.9044 | NA | NA | NA | 2.3533 | -0.4044 | -0.0159 | NA | NA | NA | NA | NA | NA | NA | NA | NA | NA | NA | NA | NA | NA | NA | NA | NA | NA | NA | NA | NA | NA | NA | NA | NA | NA | NA | NA | NA | NA | NA | NA | NA | NA | NA | NA | NA | 0.7661 | 0.7398 | 0.0359 | 0.0679 |
| sq\_Ratio\_High/Low | MYLE | 0.5255 | 0.0179 | -0.2074 | 0.1000 | NA | NA | NA | -1.9814 | NA | NA | 2.3365 | NA | NA | NA | NA | NA | NA | NA | NA | NA | NA | NA | NA | NA | NA | NA | NA | NA | NA | NA | NA | NA | NA | NA | NA | NA | NA | NA | NA | NA | NA | NA | NA | NA | NA | NA | NA | 0.6553 | 0.5978 | 0.0460 | 0.0845 |
| INT\_sq\_Ratio\_High/Low | MYLE | 0.5519 | 0.0089 | -0.1878 | -0.1028 | NA | NA | NA | -2.0368 | NA | 0.0421 | 1.6893 | NA | NA | NA | NA | NA | NA | NA | NA | NA | NA | NA | NA | NA | NA | NA | NA | NA | NA | NA | NA | NA | NA | NA | NA | NA | NA | NA | NA | NA | NA | NA | NA | NA | NA | NA | NA | 0.6627 | 0.6083 | 0.0440 | 0.0835 |
| Ratio\_SPECIES | MYLE | 0.3840 | 0.0185 | -0.2362 | -0.5922 | NA | NA | NA | NA | NA | NA | NA | 0.0033 | 0.0032 | 0.0033 | 0.0033 | NA | 0.0028 | 0.0006 | -0.0011 | 0.0033 | NA | NA | NA | NA | NA | NA | NA | NA | NA | NA | NA | NA | NA | NA | NA | NA | NA | NA | NA | NA | NA | NA | NA | NA | NA | NA | NA | 0.5428 | 0.5127 | 0.0459 | 0.0929 |
| log\_Ratio\_SPECIES | MYLE | 0.1769 | 0.0191 | -0.2264 | 1.0725 | NA | NA | NA | NA | NA | NA | NA | -0.0067 | -0.0062 | -0.0067 | -0.0067 | NA | -0.0022 | 0.0174 | 0.0302 | -0.0073 | 0.0064 | 0.0041 | 0.0064 | 0.0064 | NA | -0.0139 | -0.1030 | -0.1620 | 0.0093 | NA | NA | NA | NA | NA | NA | NA | NA | NA | NA | NA | NA | NA | NA | NA | NA | NA | NA | 0.8470 | 0.7746 | 0.0426 | 0.0637 |
| INT\_log\_Ratio\_SPECIES | MYLE | 0.1450 | 0.0213 | -0.2288 | 1.0022 | NA | NA | NA | NA | NA | NA | NA | -0.0070 | -0.0062 | -0.0070 | -0.0070 | NA | 0.0016 | 0.0327 | 0.0503 | -0.0082 | 0.0069 | 0.0045 | 0.0069 | 0.0069 | NA | -0.0151 | -0.1093 | -0.1704 | 0.0100 | 0.0000 | 0.0000 | 0.0000 | 0.0000 | NA | -0.0002 | -0.0007 | -0.0009 | 0.0000 | NA | NA | NA | NA | NA | NA | NA | NA | NA | 0.8522 | 0.7797 | 0.0431 | 0.0630 |
| sq\_Ratio\_SPECIES | MYLE | 0.3822 | 0.0184 | -0.2346 | -0.6684 | NA | NA | NA | NA | NA | NA | NA | 0.0091 | 0.0088 | 0.0091 | 0.0091 | NA | 0.0064 | -0.0075 | -0.0181 | 0.0095 | NA | NA | NA | NA | NA | NA | NA | NA | NA | NA | NA | NA | NA | NA | NA | NA | NA | NA | -0.0003 | -0.0003 | -0.0003 | -0.0003 | NA | -0.0002 | 5e-04 | 1e-03 | -3e-04 | 0.5545 | 0.5195 | 0.0467 | 0.0924 |
| INT\_sq\_Ratio\_SPECIES | MYLE | 0.4021 | 0.0152 | -0.2253 | -0.7385 | NA | NA | NA | NA | NA | NA | NA | 0.0146 | 0.0138 | 0.0146 | 0.0146 | NA | 0.0081 | -0.0256 | -0.0501 | 0.0156 | NA | NA | NA | NA | NA | NA | NA | NA | NA | -0.0004 | -0.0003 | -0.0004 | -0.0004 | NA | -0.0001 | 0.0015 | 0.0026 | -0.0004 | -0.0002 | -0.0002 | -0.0002 | -0.0002 | NA | -0.0002 | -1e-04 | 0e+00 | -2e-04 | 0.5840 | 0.5353 | 0.0465 | 0.0912 |
| Ratio\_Bats | MYLU | 0.8758 | 0.0217 | -0.4169 | -0.5516 | NA | NA | NA | NA | NA | NA | NA | NA | NA | NA | NA | NA | NA | NA | NA | NA | NA | NA | NA | NA | NA | NA | NA | NA | NA | NA | NA | NA | NA | NA | NA | NA | NA | NA | NA | NA | NA | NA | NA | NA | NA | NA | NA | 0.7213 | 0.7118 | 0.0710 | 0.1384 |
| log\_Ratio\_Bats | MYLU | -0.4109 | 0.0146 | -0.2923 | 1.3282 | -0.3887 | NA | NA | NA | NA | NA | NA | NA | NA | NA | NA | NA | NA | NA | NA | NA | NA | NA | NA | NA | NA | NA | NA | NA | NA | NA | NA | NA | NA | NA | NA | NA | NA | NA | NA | NA | NA | NA | NA | NA | NA | NA | NA | 0.8098 | 0.7877 | 0.0621 | 0.1189 |
| INT\_log\_Ratio\_Bats | MYLU | -0.1898 | 0.0067 | -0.2742 | 0.1353 | -0.3377 | 0.0556 | NA | NA | NA | NA | NA | NA | NA | NA | NA | NA | NA | NA | NA | NA | NA | NA | NA | NA | NA | NA | NA | NA | NA | NA | NA | NA | NA | NA | NA | NA | NA | NA | NA | NA | NA | NA | NA | NA | NA | NA | NA | 0.8102 | 0.7908 | 0.0620 | 0.1181 |
| sq\_Ratio\_Bats | MYLU | 1.0261 | 0.0190 | -0.3482 | -3.7486 | NA | NA | 5.3169 | NA | NA | NA | NA | NA | NA | NA | NA | NA | NA | NA | NA | NA | NA | NA | NA | NA | NA | NA | NA | NA | NA | NA | NA | NA | NA | NA | NA | NA | NA | NA | NA | NA | NA | NA | NA | NA | NA | NA | NA | 0.7802 | 0.7568 | 0.0699 | 0.1274 |
| INT\_sq\_Ratio\_Bats | MYLU | 1.0730 | 0.0045 | -0.3087 | -4.4525 | NA | 0.0963 | 3.7636 | NA | NA | NA | NA | NA | NA | NA | NA | NA | NA | NA | NA | NA | NA | NA | NA | NA | NA | NA | NA | NA | NA | NA | NA | NA | NA | NA | NA | NA | NA | NA | NA | NA | NA | NA | NA | NA | NA | NA | NA | 0.7859 | 0.7663 | 0.0669 | 0.1250 |
| Ratio\_High/Low | MYLU | 1.0030 | 0.0230 | -0.4210 | 3.6089 | NA | NA | NA | -3.5756 | NA | NA | NA | NA | NA | NA | NA | NA | NA | NA | NA | NA | NA | NA | NA | NA | NA | NA | NA | NA | NA | NA | NA | NA | NA | NA | NA | NA | NA | NA | NA | NA | NA | NA | NA | NA | NA | NA | NA | 0.7848 | 0.7711 | 0.0690 | 0.1233 |
| log\_Ratio\_High/Low | MYLU | -0.1759 | 0.0163 | -0.3170 | 0.7378 | NA | NA | NA | 0.2841 | -0.3777 | NA | NA | NA | NA | NA | NA | NA | NA | NA | NA | NA | NA | NA | NA | NA | NA | NA | NA | NA | NA | NA | NA | NA | NA | NA | NA | NA | NA | NA | NA | NA | NA | NA | NA | NA | NA | NA | NA | 0.8459 | 0.8159 | 0.0585 | 0.1108 |
| INT\_log\_Ratio\_High/Low | MYLU | 0.1504 | 0.0029 | -0.2914 | 0.2665 | NA | NA | NA | -0.6917 | -0.2974 | 0.0649 | NA | NA | NA | NA | NA | NA | NA | NA | NA | NA | NA | NA | NA | NA | NA | NA | NA | NA | NA | NA | NA | NA | NA | NA | NA | NA | NA | NA | NA | NA | NA | NA | NA | NA | NA | NA | NA | 0.8482 | 0.8221 | 0.0559 | 0.1090 |
| sq\_Ratio\_High/Low | MYLU | 1.1711 | 0.0191 | -0.3364 | -0.0951 | NA | NA | NA | -3.9122 | NA | NA | 4.7973 | NA | NA | NA | NA | NA | NA | NA | NA | NA | NA | NA | NA | NA | NA | NA | NA | NA | NA | NA | NA | NA | NA | NA | NA | NA | NA | NA | NA | NA | NA | NA | NA | NA | NA | NA | NA | 0.8397 | 0.8094 | 0.0634 | 0.1128 |
| INT\_sq\_Ratio\_High/Low | MYLU | 1.2193 | 0.0026 | -0.3009 | -0.4639 | NA | NA | NA | -4.0130 | NA | 0.0766 | 3.6204 | NA | NA | NA | NA | NA | NA | NA | NA | NA | NA | NA | NA | NA | NA | NA | NA | NA | NA | NA | NA | NA | NA | NA | NA | NA | NA | NA | NA | NA | NA | NA | NA | NA | NA | NA | NA | 0.8429 | 0.8186 | 0.0588 | 0.1101 |
| Ratio\_SPECIES | MYLU | 0.8889 | 0.0199 | -0.3902 | -1.5410 | NA | NA | NA | NA | NA | NA | NA | 0.0084 | -0.0013 | 0.0084 | 0.0084 | 0.0090 | NA | 0.0073 | 0.0040 | 0.0042 | NA | NA | NA | NA | NA | NA | NA | NA | NA | NA | NA | NA | NA | NA | NA | NA | NA | NA | NA | NA | NA | NA | NA | NA | NA | NA | NA | 0.7362 | 0.7231 | 0.0731 | 0.1358 |
| log\_Ratio\_SPECIES | MYLU | 0.6239 | 0.0207 | -0.3777 | 0.5893 | NA | NA | NA | NA | NA | NA | NA | -0.0099 | 0.0447 | -0.0099 | -0.0099 | -0.0131 | NA | -0.0009 | 0.0203 | 0.0181 | 0.0309 | -0.2319 | 0.0309 | 0.0309 | 0.0461 | NA | -0.0107 | -0.1105 | -0.1009 | NA | NA | NA | NA | NA | NA | NA | NA | NA | NA | NA | NA | NA | NA | NA | NA | NA | NA | 0.9119 | 0.8596 | 0.0581 | 0.0973 |
| INT\_log\_Ratio\_SPECIES | MYLU | 0.6385 | 0.0197 | -0.3766 | 0.6217 | NA | NA | NA | NA | NA | NA | NA | -0.0152 | 0.0438 | -0.0153 | -0.0152 | -0.0187 | NA | -0.0017 | 0.0246 | 0.0201 | 0.0330 | -0.2317 | 0.0331 | 0.0331 | 0.0484 | NA | -0.0105 | -0.1125 | -0.1020 | 0.0002 | 0.0000 | 0.0002 | 0.0002 | 0.0003 | NA | 0.0000 | -0.0002 | -0.0001 | NA | NA | NA | NA | NA | NA | NA | NA | NA | 0.9116 | 0.8599 | 0.0584 | 0.0974 |
| sq\_Ratio\_SPECIES | MYLU | 0.8866 | 0.0197 | -0.3882 | -1.6377 | NA | NA | NA | NA | NA | NA | NA | 0.0217 | -0.0371 | 0.0217 | 0.0217 | 0.0249 | NA | 0.0146 | -0.0054 | -0.0039 | NA | NA | NA | NA | NA | NA | NA | NA | NA | NA | NA | NA | NA | NA | NA | NA | NA | NA | -0.0008 | 0.0022 | -0.0008 | -0.0008 | -0.0009 | NA | -4e-04 | 6e-04 | 5e-04 | 0.7473 | 0.7307 | 0.0752 | 0.1341 |
| INT\_sq\_Ratio\_SPECIES | MYLU | 0.9434 | 0.0106 | -0.3617 | -1.8373 | NA | NA | NA | NA | NA | NA | NA | 0.0314 | -0.0926 | 0.0313 | 0.0314 | 0.0385 | NA | 0.0149 | -0.0291 | -0.0264 | NA | NA | NA | NA | NA | NA | NA | NA | NA | -0.0006 | 0.0045 | -0.0006 | -0.0006 | -0.0009 | NA | 0.0001 | 0.0020 | 0.0019 | -0.0006 | 0.0004 | -0.0006 | -0.0006 | -0.0006 | NA | -5e-04 | -3e-04 | -3e-04 | 0.7718 | 0.7501 | 0.0735 | 0.1300 |
| Ratio\_Bats | MYSE | 0.6109 | 0.0254 | -0.3602 | -0.3656 | NA | NA | NA | NA | NA | NA | NA | NA | NA | NA | NA | NA | NA | NA | NA | NA | NA | NA | NA | NA | NA | NA | NA | NA | NA | NA | NA | NA | NA | NA | NA | NA | NA | NA | NA | NA | NA | NA | NA | NA | NA | NA | NA | 0.6402 | 0.6242 | 0.0607 | 0.1171 |
| log\_Ratio\_Bats | MYSE | -0.6593 | 0.0183 | -0.2372 | 1.4901 | -0.3837 | NA | NA | NA | NA | NA | NA | NA | NA | NA | NA | NA | NA | NA | NA | NA | NA | NA | NA | NA | NA | NA | NA | NA | NA | NA | NA | NA | NA | NA | NA | NA | NA | NA | NA | NA | NA | NA | NA | NA | NA | NA | NA | 0.7827 | 0.7591 | 0.0503 | 0.0938 |
| INT\_log\_Ratio\_Bats | MYSE | -0.6567 | 0.0182 | -0.2370 | 1.4761 | -0.3831 | 0.0006 | NA | NA | NA | NA | NA | NA | NA | NA | NA | NA | NA | NA | NA | NA | NA | NA | NA | NA | NA | NA | NA | NA | NA | NA | NA | NA | NA | NA | NA | NA | NA | NA | NA | NA | NA | NA | NA | NA | NA | NA | NA | 0.7827 | 0.7591 | 0.0503 | 0.0939 |
| sq\_Ratio\_Bats | MYSE | 0.7221 | 0.0234 | -0.3093 | -2.7301 | NA | NA | 3.9324 | NA | NA | NA | NA | NA | NA | NA | NA | NA | NA | NA | NA | NA | NA | NA | NA | NA | NA | NA | NA | NA | NA | NA | NA | NA | NA | NA | NA | NA | NA | NA | NA | NA | NA | NA | NA | NA | NA | NA | NA | 0.7042 | 0.6691 | 0.0606 | 0.1100 |
| INT\_sq\_Ratio\_Bats | MYSE | 0.7581 | 0.0122 | -0.2790 | -3.2706 | NA | 0.0739 | 2.7398 | NA | NA | NA | NA | NA | NA | NA | NA | NA | NA | NA | NA | NA | NA | NA | NA | NA | NA | NA | NA | NA | NA | NA | NA | NA | NA | NA | NA | NA | NA | NA | NA | NA | NA | NA | NA | NA | NA | NA | NA | 0.7138 | 0.6794 | 0.0583 | 0.1085 |
| Ratio\_High/Low | MYSE | 0.7140 | 0.0264 | -0.3635 | 3.0067 | NA | NA | NA | -2.8981 | NA | NA | NA | NA | NA | NA | NA | NA | NA | NA | NA | NA | NA | NA | NA | NA | NA | NA | NA | NA | NA | NA | NA | NA | NA | NA | NA | NA | NA | NA | NA | NA | NA | NA | NA | NA | NA | NA | NA | 0.7235 | 0.6953 | 0.0594 | 0.1054 |
| log\_Ratio\_High/Low | MYSE | -0.6697 | 0.0185 | -0.2414 | -0.3632 | NA | NA | NA | 1.6320 | -0.4433 | NA | NA | NA | NA | NA | NA | NA | NA | NA | NA | NA | NA | NA | NA | NA | NA | NA | NA | NA | NA | NA | NA | NA | NA | NA | NA | NA | NA | NA | NA | NA | NA | NA | NA | NA | NA | NA | NA | 0.8351 | 0.8078 | 0.0450 | 0.0839 |
| INT\_log\_Ratio\_High/Low | MYSE | -0.5811 | 0.0149 | -0.2345 | -0.4911 | NA | NA | NA | 1.3670 | -0.4215 | 0.0176 | NA | NA | NA | NA | NA | NA | NA | NA | NA | NA | NA | NA | NA | NA | NA | NA | NA | NA | NA | NA | NA | NA | NA | NA | NA | NA | NA | NA | NA | NA | NA | NA | NA | NA | NA | NA | NA | 0.8347 | 0.8086 | 0.0448 | 0.0837 |
| sq\_Ratio\_High/Low | MYSE | 0.8434 | 0.0234 | -0.2984 | 0.1566 | NA | NA | NA | -3.1572 | NA | NA | 3.6913 | NA | NA | NA | NA | NA | NA | NA | NA | NA | NA | NA | NA | NA | NA | NA | NA | NA | NA | NA | NA | NA | NA | NA | NA | NA | NA | NA | NA | NA | NA | NA | NA | NA | NA | NA | NA | 0.7825 | 0.7366 | 0.0572 | 0.0982 |
| INT\_sq\_Ratio\_High/Low | MYSE | 0.8850 | 0.0092 | -0.2677 | -0.1623 | NA | NA | NA | -3.2443 | NA | 0.0663 | 2.6735 | NA | NA | NA | NA | NA | NA | NA | NA | NA | NA | NA | NA | NA | NA | NA | NA | NA | NA | NA | NA | NA | NA | NA | NA | NA | NA | NA | NA | NA | NA | NA | NA | NA | NA | NA | NA | 0.7901 | 0.7492 | 0.0535 | 0.0960 |
| Ratio\_SPECIES | MYSE | 0.6201 | 0.0241 | -0.3413 | -1.0655 | NA | NA | NA | NA | NA | NA | NA | 0.0059 | 0.0060 | 0.0059 | 0.0059 | 0.0014 | 0.0000 | NA | 0.0030 | 0.0061 | NA | NA | NA | NA | NA | NA | NA | NA | NA | NA | NA | NA | NA | NA | NA | NA | NA | NA | NA | NA | NA | NA | NA | NA | NA | NA | NA | 0.6554 | 0.6345 | 0.0616 | 0.1155 |
| log\_Ratio\_SPECIES | MYSE | 0.3645 | 0.0249 | -0.3292 | 0.9894 | NA | NA | NA | NA | NA | NA | NA | -0.0098 | -0.0097 | -0.0098 | -0.0098 | 0.0262 | 0.0349 | NA | 0.0148 | -0.0111 | 0.0217 | 0.0220 | 0.0217 | 0.0217 | -0.1437 | -0.1850 | NA | -0.0904 | 0.0278 | NA | NA | NA | NA | NA | NA | NA | NA | NA | NA | NA | NA | NA | NA | NA | NA | NA | NA | 0.9028 | 0.8460 | 0.0513 | 0.0756 |
| INT\_log\_Ratio\_SPECIES | MYSE | 0.3404 | 0.0265 | -0.3310 | 0.9362 | NA | NA | NA | NA | NA | NA | NA | -0.0123 | -0.0111 | -0.0123 | -0.0123 | 0.0404 | 0.0503 | NA | 0.0258 | -0.0145 | 0.0231 | 0.0229 | 0.0231 | 0.0231 | -0.1496 | -0.1915 | NA | -0.0949 | 0.0296 | 0.0001 | 0.0001 | 0.0001 | 0.0001 | -0.0006 | -0.0007 | NA | -0.0005 | 0.0002 | NA | NA | NA | NA | NA | NA | NA | NA | NA | 0.9054 | 0.8478 | 0.0517 | 0.0752 |
| sq\_Ratio\_SPECIES | MYSE | 0.6152 | 0.0238 | -0.3369 | -1.2783 | NA | NA | NA | NA | NA | NA | NA | 0.0172 | 0.0177 | 0.0172 | 0.0172 | -0.0111 | -0.0198 | NA | -0.0010 | 0.0182 | NA | NA | NA | NA | NA | NA | NA | NA | NA | NA | NA | NA | NA | NA | NA | NA | NA | NA | -0.0006 | -0.0006 | -0.0006 | -0.0006 | 0.0008 | 0.0012 | NA | 3e-04 | -7e-04 | 0.6665 | 0.6421 | 0.0631 | 0.1145 |
| INT\_sq\_Ratio\_SPECIES | MYSE | 0.6508 | 0.0180 | -0.3203 | -1.4035 | NA | NA | NA | NA | NA | NA | NA | 0.0262 | 0.0273 | 0.0262 | 0.0262 | -0.0400 | -0.0590 | NA | -0.0169 | 0.0285 | NA | NA | NA | NA | NA | NA | NA | NA | NA | -0.0006 | -0.0006 | -0.0006 | -0.0006 | 0.0024 | 0.0032 | NA | 0.0014 | -0.0007 | -0.0004 | -0.0004 | -0.0004 | -0.0004 | -0.0001 | 0.0000 | NA | -3e-04 | -4e-04 | 0.6957 | 0.6604 | 0.0624 | 0.1121 |
| Ratio\_Bats | MYSO | 0.9373 | 0.0180 | -0.4094 | -0.6091 | NA | NA | NA | NA | NA | NA | NA | NA | NA | NA | NA | NA | NA | NA | NA | NA | NA | NA | NA | NA | NA | NA | NA | NA | NA | NA | NA | NA | NA | NA | NA | NA | NA | NA | NA | NA | NA | NA | NA | NA | NA | NA | NA | 0.7307 | 0.7209 | 0.0805 | 0.1460 |
| log\_Ratio\_Bats | MYSO | -0.3134 | 0.0110 | -0.2883 | 1.2182 | -0.3778 | NA | NA | NA | NA | NA | NA | NA | NA | NA | NA | NA | NA | NA | NA | NA | NA | NA | NA | NA | NA | NA | NA | NA | NA | NA | NA | NA | NA | NA | NA | NA | NA | NA | NA | NA | NA | NA | NA | NA | NA | NA | NA | 0.8075 | 0.7832 | 0.0727 | 0.1288 |
| INT\_log\_Ratio\_Bats | MYSO | -0.0204 | 0.0005 | -0.2643 | -0.3625 | -0.3103 | 0.0737 | NA | NA | NA | NA | NA | NA | NA | NA | NA | NA | NA | NA | NA | NA | NA | NA | NA | NA | NA | NA | NA | NA | NA | NA | NA | NA | NA | NA | NA | NA | NA | NA | NA | NA | NA | NA | NA | NA | NA | NA | NA | 0.8092 | 0.7881 | 0.0732 | 0.1274 |
| sq\_Ratio\_Bats | MYSO | 1.0965 | 0.0151 | -0.3367 | -3.9929 | NA | NA | 5.6276 | NA | NA | NA | NA | NA | NA | NA | NA | NA | NA | NA | NA | NA | NA | NA | NA | NA | NA | NA | NA | NA | NA | NA | NA | NA | NA | NA | NA | NA | NA | NA | NA | NA | NA | NA | NA | NA | NA | NA | NA | 0.7877 | 0.7647 | 0.0785 | 0.1343 |
| INT\_sq\_Ratio\_Bats | MYSO | 1.1460 | -0.0002 | -0.2950 | -4.7362 | NA | 0.1017 | 3.9875 | NA | NA | NA | NA | NA | NA | NA | NA | NA | NA | NA | NA | NA | NA | NA | NA | NA | NA | NA | NA | NA | NA | NA | NA | NA | NA | NA | NA | NA | NA | NA | NA | NA | NA | NA | NA | NA | NA | NA | NA | 0.7925 | 0.7740 | 0.0765 | 0.1318 |
| Ratio\_High/Low | MYSO | 1.0740 | 0.0193 | -0.4138 | 3.8603 | NA | NA | NA | -3.8411 | NA | NA | NA | NA | NA | NA | NA | NA | NA | NA | NA | NA | NA | NA | NA | NA | NA | NA | NA | NA | NA | NA | NA | NA | NA | NA | NA | NA | NA | NA | NA | NA | NA | NA | NA | NA | NA | NA | NA | 0.7985 | 0.7804 | 0.0776 | 0.1295 |
| log\_Ratio\_High/Low | MYSO | 0.0138 | 0.0133 | -0.3203 | 1.2784 | NA | NA | NA | -0.3701 | -0.3396 | NA | NA | NA | NA | NA | NA | NA | NA | NA | NA | NA | NA | NA | NA | NA | NA | NA | NA | NA | NA | NA | NA | NA | NA | NA | NA | NA | NA | NA | NA | NA | NA | NA | NA | NA | NA | NA | NA | 0.8453 | 0.8119 | 0.0692 | 0.1201 |
| INT\_log\_Ratio\_High/Low | MYSO | 0.4143 | -0.0031 | -0.2889 | 0.7001 | NA | NA | NA | -1.5676 | -0.2411 | 0.0796 | NA | NA | NA | NA | NA | NA | NA | NA | NA | NA | NA | NA | NA | NA | NA | NA | NA | NA | NA | NA | NA | NA | NA | NA | NA | NA | NA | NA | NA | NA | NA | NA | NA | NA | NA | NA | NA | 0.8493 | 0.8201 | 0.0668 | 0.1175 |
| sq\_Ratio\_High/Low | MYSO | 1.2468 | 0.0153 | -0.3269 | 0.0536 | NA | NA | NA | -4.1870 | NA | NA | 4.9303 | NA | NA | NA | NA | NA | NA | NA | NA | NA | NA | NA | NA | NA | NA | NA | NA | NA | NA | NA | NA | NA | NA | NA | NA | NA | NA | NA | NA | NA | NA | NA | NA | NA | NA | NA | NA | 0.8492 | 0.8156 | 0.0713 | 0.1189 |
| INT\_sq\_Ratio\_High/Low | MYSO | 1.2964 | -0.0016 | -0.2903 | -0.3264 | NA | NA | NA | -4.2908 | NA | 0.0789 | 3.7178 | NA | NA | NA | NA | NA | NA | NA | NA | NA | NA | NA | NA | NA | NA | NA | NA | NA | NA | NA | NA | NA | NA | NA | NA | NA | NA | NA | NA | NA | NA | NA | NA | NA | NA | NA | NA | 0.8515 | 0.8241 | 0.0670 | 0.1163 |
| Ratio\_SPECIES | MYSO | 0.9514 | 0.0160 | -0.3807 | -1.6719 | NA | NA | NA | NA | NA | NA | NA | 0.0093 | 0.0092 | 0.0093 | 0.0093 | 0.0006 | 0.0006 | 0.0047 | NA | 0.0091 | NA | NA | NA | NA | NA | NA | NA | NA | NA | NA | NA | NA | NA | NA | NA | NA | NA | NA | NA | NA | NA | NA | NA | NA | NA | NA | NA | 0.7456 | 0.7324 | 0.0829 | 0.1431 |
| log\_Ratio\_SPECIES | MYSO | 0.6935 | 0.0168 | -0.3686 | 0.4014 | NA | NA | NA | NA | NA | NA | NA | -0.0105 | -0.0071 | -0.0105 | -0.0105 | 0.0364 | 0.0365 | 0.0179 | NA | -0.0089 | 0.0381 | 0.0239 | 0.0381 | 0.0381 | -0.1891 | -0.1897 | -0.0969 | NA | 0.0308 | NA | NA | NA | NA | NA | NA | NA | NA | NA | NA | NA | NA | NA | NA | NA | NA | NA | NA | 0.9091 | 0.8546 | 0.0662 | 0.1061 |
| INT\_log\_Ratio\_SPECIES | MYSO | 0.7230 | 0.0147 | -0.3664 | 0.4664 | NA | NA | NA | NA | NA | NA | NA | -0.0165 | -0.0087 | -0.0166 | -0.0165 | 0.0316 | 0.0321 | 0.0175 | NA | -0.0140 | 0.0404 | 0.0243 | 0.0404 | 0.0404 | -0.1873 | -0.1881 | -0.0971 | NA | 0.0327 | 0.0003 | 0.0001 | 0.0003 | 0.0003 | 0.0002 | 0.0002 | 0.0000 | NA | 0.0002 | NA | NA | NA | NA | NA | NA | NA | NA | NA | 0.9082 | 0.8553 | 0.0663 | 0.1061 |
| sq\_Ratio\_SPECIES | MYSO | 0.9503 | 0.0159 | -0.3798 | -1.7180 | NA | NA | NA | NA | NA | NA | NA | 0.0238 | 0.0227 | 0.0238 | 0.0238 | -0.0281 | -0.0282 | -0.0038 | NA | 0.0226 | NA | NA | NA | NA | NA | NA | NA | NA | NA | NA | NA | NA | NA | NA | NA | NA | NA | NA | -0.0009 | -0.0008 | -0.0009 | -0.0009 | 0.0017 | 0.0017 | 5e-04 | NA | -8e-04 | 0.7570 | 0.7403 | 0.0847 | 0.1413 |
| INT\_sq\_Ratio\_SPECIES | MYSO | 1.0133 | 0.0058 | -0.3504 | -1.9390 | NA | NA | NA | NA | NA | NA | NA | 0.0346 | 0.0314 | 0.0346 | 0.0347 | -0.0747 | -0.0747 | -0.0262 | NA | 0.0319 | NA | NA | NA | NA | NA | NA | NA | NA | NA | -0.0007 | -0.0005 | -0.0007 | -0.0007 | 0.0038 | 0.0038 | 0.0019 | NA | -0.0005 | -0.0007 | -0.0007 | -0.0007 | -0.0007 | 0.0002 | 0.0002 | -3e-04 | NA | -7e-04 | 0.7808 | 0.7605 | 0.0820 | 0.1365 |
| Ratio\_Bats | PESU | 0.8497 | 0.0163 | -0.3716 | -0.5322 | NA | NA | NA | NA | NA | NA | NA | NA | NA | NA | NA | NA | NA | NA | NA | NA | NA | NA | NA | NA | NA | NA | NA | NA | NA | NA | NA | NA | NA | NA | NA | NA | NA | NA | NA | NA | NA | NA | NA | NA | NA | NA | NA | 0.7016 | 0.6930 | 0.0697 | 0.1414 |
| log\_Ratio\_Bats | PESU | -0.3568 | 0.0096 | -0.2548 | 1.2305 | -0.3644 | NA | NA | NA | NA | NA | NA | NA | NA | NA | NA | NA | NA | NA | NA | NA | NA | NA | NA | NA | NA | NA | NA | NA | NA | NA | NA | NA | NA | NA | NA | NA | NA | NA | NA | NA | NA | NA | NA | NA | NA | NA | NA | 0.7785 | 0.7611 | 0.0628 | 0.1248 |
| INT\_log\_Ratio\_Bats | PESU | -0.1709 | 0.0030 | -0.2396 | 0.2274 | -0.3216 | 0.0468 | NA | NA | NA | NA | NA | NA | NA | NA | NA | NA | NA | NA | NA | NA | NA | NA | NA | NA | NA | NA | NA | NA | NA | NA | NA | NA | NA | NA | NA | NA | NA | NA | NA | NA | NA | NA | NA | NA | NA | NA | NA | 0.7781 | 0.7634 | 0.0634 | 0.1242 |
| sq\_Ratio\_Bats | PESU | 0.9892 | 0.0138 | -0.3078 | -3.4995 | NA | NA | 4.9349 | NA | NA | NA | NA | NA | NA | NA | NA | NA | NA | NA | NA | NA | NA | NA | NA | NA | NA | NA | NA | NA | NA | NA | NA | NA | NA | NA | NA | NA | NA | NA | NA | NA | NA | NA | NA | NA | NA | NA | NA | 0.7521 | 0.7326 | 0.0690 | 0.1321 |
| INT\_sq\_Ratio\_Bats | PESU | 1.0312 | 0.0008 | -0.2725 | -4.1296 | NA | 0.0862 | 3.5443 | NA | NA | NA | NA | NA | NA | NA | NA | NA | NA | NA | NA | NA | NA | NA | NA | NA | NA | NA | NA | NA | NA | NA | NA | NA | NA | NA | NA | NA | NA | NA | NA | NA | NA | NA | NA | NA | NA | NA | NA | 0.7555 | 0.7404 | 0.0678 | 0.1303 |
| Ratio\_High/Low | PESU | 0.9403 | 0.0172 | -0.3745 | 2.4325 | NA | NA | NA | -2.5478 | NA | NA | NA | NA | NA | NA | NA | NA | NA | NA | NA | NA | NA | NA | NA | NA | NA | NA | NA | NA | NA | NA | NA | NA | NA | NA | NA | NA | NA | NA | NA | NA | NA | NA | NA | NA | NA | NA | NA | 0.7331 | 0.7238 | 0.0721 | 0.1341 |
| log\_Ratio\_High/Low | PESU | -0.1753 | 0.0109 | -0.2761 | -0.2844 | NA | NA | NA | 1.1045 | -0.3574 | NA | NA | NA | NA | NA | NA | NA | NA | NA | NA | NA | NA | NA | NA | NA | NA | NA | NA | NA | NA | NA | NA | NA | NA | NA | NA | NA | NA | NA | NA | NA | NA | NA | NA | NA | NA | NA | NA | 0.7824 | 0.7647 | 0.0625 | 0.1239 |
| INT\_log\_Ratio\_High/Low | PESU | 0.0856 | 0.0002 | -0.2557 | -0.6610 | NA | NA | NA | 0.3246 | -0.2932 | 0.0518 | NA | NA | NA | NA | NA | NA | NA | NA | NA | NA | NA | NA | NA | NA | NA | NA | NA | NA | NA | NA | NA | NA | NA | NA | NA | NA | NA | NA | NA | NA | NA | NA | NA | NA | NA | NA | NA | 0.7836 | 0.7688 | 0.0625 | 0.1229 |
| sq\_Ratio\_High/Low | PESU | 1.1065 | 0.0134 | -0.2909 | -1.2273 | NA | NA | NA | -2.8805 | NA | NA | 4.7400 | NA | NA | NA | NA | NA | NA | NA | NA | NA | NA | NA | NA | NA | NA | NA | NA | NA | NA | NA | NA | NA | NA | NA | NA | NA | NA | NA | NA | NA | NA | NA | NA | NA | NA | NA | NA | 0.7818 | 0.7619 | 0.0666 | 0.1247 |
| INT\_sq\_Ratio\_High/Low | PESU | 1.1443 | 0.0004 | -0.2630 | -1.5173 | NA | NA | NA | -2.9597 | NA | 0.0602 | 3.8147 | NA | NA | NA | NA | NA | NA | NA | NA | NA | NA | NA | NA | NA | NA | NA | NA | NA | NA | NA | NA | NA | NA | NA | NA | NA | NA | NA | NA | NA | NA | NA | NA | NA | NA | NA | NA | 0.7819 | 0.7677 | 0.0647 | 0.1232 |
| Ratio\_SPECIES | PESU | 0.8607 | 0.0148 | -0.3491 | -1.3664 | NA | NA | NA | NA | NA | NA | NA | 0.0067 | -0.0051 | 0.0067 | 0.0067 | 0.0067 | 0.0058 | 0.0067 | 0.0068 | NA | NA | NA | NA | NA | NA | NA | NA | NA | NA | NA | NA | NA | NA | NA | NA | NA | NA | NA | NA | NA | NA | NA | NA | NA | NA | NA | NA | 0.7138 | 0.7023 | 0.0725 | 0.1393 |
| log\_Ratio\_SPECIES | PESU | 0.5836 | 0.0156 | -0.3360 | 0.8612 | NA | NA | NA | NA | NA | NA | NA | -0.0038 | 0.0543 | -0.0038 | -0.0038 | -0.0037 | 0.0004 | -0.0038 | -0.0043 | NA | -0.0032 | -0.2888 | -0.0032 | -0.0031 | -0.0036 | -0.0241 | -0.0029 | -0.0009 | NA | NA | NA | NA | NA | NA | NA | NA | NA | NA | NA | NA | NA | NA | NA | NA | NA | NA | NA | 0.9086 | 0.8492 | 0.0550 | 0.0997 |
| INT\_log\_Ratio\_SPECIES | PESU | 0.6103 | 0.0138 | -0.3340 | 0.9200 | NA | NA | NA | NA | NA | NA | NA | -0.0073 | 0.0485 | -0.0073 | -0.0073 | -0.0073 | -0.0035 | -0.0074 | -0.0080 | NA | -0.0019 | -0.2866 | -0.0019 | -0.0019 | -0.0023 | -0.0226 | -0.0016 | 0.0005 | NA | 0.0002 | 0.0003 | 0.0002 | 0.0002 | 0.0002 | 0.0002 | 0.0002 | 0.0002 | NA | NA | NA | NA | NA | NA | NA | NA | NA | NA | 0.9076 | 0.8499 | 0.0551 | 0.0997 |
| sq\_Ratio\_SPECIES | PESU | 0.8673 | 0.0152 | -0.3548 | -1.0842 | NA | NA | NA | NA | NA | NA | NA | 0.0108 | -0.0591 | 0.0109 | 0.0109 | 0.0107 | 0.0063 | 0.0109 | 0.0113 | NA | NA | NA | NA | NA | NA | NA | NA | NA | NA | NA | NA | NA | NA | NA | NA | NA | NA | NA | -0.0003 | 0.0031 | -0.0003 | -0.0003 | -0.0003 | -0.0001 | -3e-04 | -4e-04 | NA | 0.7318 | 0.7128 | 0.0732 | 0.1371 |
| INT\_sq\_Ratio\_SPECIES | PESU | 0.9253 | 0.0059 | -0.3277 | -1.2878 | NA | NA | NA | NA | NA | NA | NA | 0.0129 | -0.1284 | 0.0130 | 0.0130 | 0.0127 | 0.0025 | 0.0131 | 0.0139 | NA | NA | NA | NA | NA | NA | NA | NA | NA | NA | 0.0000 | 0.0056 | 0.0000 | 0.0000 | 0.0000 | 0.0005 | 0.0000 | 0.0000 | NA | -0.0004 | 0.0009 | -0.0004 | -0.0004 | -0.0004 | -0.0004 | -4e-04 | -4e-04 | NA | 0.7580 | 0.7334 | 0.0715 | 0.1329 |

## GLM Coefs p-values

p-values

| Vars | Species | (Intercept) | Ex\_Count | log\_Ex\_Count | Ratio\_Bats | log\_Ratio\_Bats | Ex\_Count\_Ratio\_Bats | sq\_Ratio\_Bats | Ratio\_High/Low | log\_Ratio\_High/Low | Ex\_Count\_Ratio\_High/Low | sq\_Ratio\_High/Low | Ratio\_EPFU | Ratio\_LABO | Ratio\_LACI | Ratio\_LANO | Ratio\_MYLE | Ratio\_MYLU | Ratio\_MYSE | Ratio\_MYSO | Ratio\_PESU | log\_Ratio\_EPFU | log\_Ratio\_LABO | log\_Ratio\_LACI | log\_Ratio\_LANO | log\_Ratio\_MYLE | log\_Ratio\_MYLU | log\_Ratio\_MYSE | log\_Ratio\_MYSO | log\_Ratio\_PESU | Ex\_Count\_Ratio\_EPFU | Ex\_Count\_Ratio\_LABO | Ex\_Count\_Ratio\_LACI | Ex\_Count\_Ratio\_LANO | Ex\_Count\_Ratio\_MYLE | Ex\_Count\_Ratio\_MYLU | Ex\_Count\_Ratio\_MYSE | Ex\_Count\_Ratio\_MYSO | Ex\_Count\_Ratio\_PESU | sq\_Ratio\_EPFU | sq\_Ratio\_LABO | sq\_Ratio\_LACI | sq\_Ratio\_LANO | sq\_Ratio\_MYLE | sq\_Ratio\_MYLU | sq\_Ratio\_MYSE | sq\_Ratio\_MYSO | sq\_Ratio\_PESU |
| --- | --- | --- | --- | --- | --- | --- | --- | --- | --- | --- | --- | --- | --- | --- | --- | --- | --- | --- | --- | --- | --- | --- | --- | --- | --- | --- | --- | --- | --- | --- | --- | --- | --- | --- | --- | --- | --- | --- | --- | --- | --- | --- | --- | --- | --- | --- | --- | --- |
| Ratio\_Bats | EPFU | 0.0000 | 0.0000 | 0 | 0.0000 | NA | NA | NA | NA | NA | NA | NA | NA | NA | NA | NA | NA | NA | NA | NA | NA | NA | NA | NA | NA | NA | NA | NA | NA | NA | NA | NA | NA | NA | NA | NA | NA | NA | NA | NA | NA | NA | NA | NA | NA | NA | NA | NA |
| log\_Ratio\_Bats | EPFU | 0.0000 | 0.0000 | 0 | 0.0000 | 0 | NA | NA | NA | NA | NA | NA | NA | NA | NA | NA | NA | NA | NA | NA | NA | NA | NA | NA | NA | NA | NA | NA | NA | NA | NA | NA | NA | NA | NA | NA | NA | NA | NA | NA | NA | NA | NA | NA | NA | NA | NA | NA |
| INT\_log\_Ratio\_Bats | EPFU | 0.0000 | 0.0000 | 0 | 0.0000 | 0 | 0.0599 | NA | NA | NA | NA | NA | NA | NA | NA | NA | NA | NA | NA | NA | NA | NA | NA | NA | NA | NA | NA | NA | NA | NA | NA | NA | NA | NA | NA | NA | NA | NA | NA | NA | NA | NA | NA | NA | NA | NA | NA | NA |
| sq\_Ratio\_Bats | EPFU | 0.0000 | 0.0000 | 0 | 0.0000 | NA | NA | 0 | NA | NA | NA | NA | NA | NA | NA | NA | NA | NA | NA | NA | NA | NA | NA | NA | NA | NA | NA | NA | NA | NA | NA | NA | NA | NA | NA | NA | NA | NA | NA | NA | NA | NA | NA | NA | NA | NA | NA | NA |
| INT\_sq\_Ratio\_Bats | EPFU | 0.0000 | 0.0000 | 0 | 0.0000 | NA | 0.0000 | 0 | NA | NA | NA | NA | NA | NA | NA | NA | NA | NA | NA | NA | NA | NA | NA | NA | NA | NA | NA | NA | NA | NA | NA | NA | NA | NA | NA | NA | NA | NA | NA | NA | NA | NA | NA | NA | NA | NA | NA | NA |
| Ratio\_High/Low | EPFU | 0.0000 | 0.0000 | 0 | 0.0000 | NA | NA | NA | 0.0000 | NA | NA | NA | NA | NA | NA | NA | NA | NA | NA | NA | NA | NA | NA | NA | NA | NA | NA | NA | NA | NA | NA | NA | NA | NA | NA | NA | NA | NA | NA | NA | NA | NA | NA | NA | NA | NA | NA | NA |
| log\_Ratio\_High/Low | EPFU | 0.0000 | 0.0000 | 0 | 0.0000 | NA | NA | NA | 0.0000 | 0e+00 | NA | NA | NA | NA | NA | NA | NA | NA | NA | NA | NA | NA | NA | NA | NA | NA | NA | NA | NA | NA | NA | NA | NA | NA | NA | NA | NA | NA | NA | NA | NA | NA | NA | NA | NA | NA | NA | NA |
| INT\_log\_Ratio\_High/Low | EPFU | 0.0000 | 0.4936 | 0 | 0.0158 | NA | NA | NA | 0.0000 | 0e+00 | 0 | NA | NA | NA | NA | NA | NA | NA | NA | NA | NA | NA | NA | NA | NA | NA | NA | NA | NA | NA | NA | NA | NA | NA | NA | NA | NA | NA | NA | NA | NA | NA | NA | NA | NA | NA | NA | NA |
| sq\_Ratio\_High/Low | EPFU | 0.0000 | 0.0000 | 0 | 0.0000 | NA | NA | NA | 0.0000 | NA | NA | 0 | NA | NA | NA | NA | NA | NA | NA | NA | NA | NA | NA | NA | NA | NA | NA | NA | NA | NA | NA | NA | NA | NA | NA | NA | NA | NA | NA | NA | NA | NA | NA | NA | NA | NA | NA | NA |
| INT\_sq\_Ratio\_High/Low | EPFU | 0.0000 | 0.0000 | 0 | 0.0000 | NA | NA | NA | 0.0000 | NA | 0 | 0 | NA | NA | NA | NA | NA | NA | NA | NA | NA | NA | NA | NA | NA | NA | NA | NA | NA | NA | NA | NA | NA | NA | NA | NA | NA | NA | NA | NA | NA | NA | NA | NA | NA | NA | NA | NA |
| Ratio\_SPECIES | EPFU | 0.0000 | 0.0000 | 0 | 0.0000 | NA | NA | NA | NA | NA | NA | NA | NA | 0.0000 | 0.1973 | 0.1071 | 0.0000 | 0.0000 | 0.0000 | 0.0000 | 0.0000 | NA | NA | NA | NA | NA | NA | NA | NA | NA | NA | NA | NA | NA | NA | NA | NA | NA | NA | NA | NA | NA | NA | NA | NA | NA | NA | NA |
| log\_Ratio\_SPECIES | EPFU | 0.0000 | 0.0000 | 0 | 0.0000 | NA | NA | NA | NA | NA | NA | NA | NA | 0.0000 | 0.0000 | 0.0000 | 0.0000 | 0.0000 | 0.0000 | 0.0000 | 0.0000 | NA | 0.0149 | 0.0000 | 0.0000 | 0.0029 | 0.0026 | 0.0028 | 0.0028 | 0.0026 | NA | NA | NA | NA | NA | NA | NA | NA | NA | NA | NA | NA | NA | NA | NA | NA | NA | NA |
| INT\_log\_Ratio\_SPECIES | EPFU | 0.0000 | 0.0000 | 0 | 0.0000 | NA | NA | NA | NA | NA | NA | NA | NA | 0.0483 | 0.0000 | 0.0000 | 0.0334 | 0.0327 | 0.0332 | 0.0332 | 0.0327 | NA | 0.0172 | 0.0000 | 0.0000 | 0.0038 | 0.0035 | 0.0037 | 0.0037 | 0.0035 | NA | 0.6764 | 0.0030 | 0.0027 | 0.6344 | 0.6321 | 0.6339 | 0.6340 | 0.6321 | NA | NA | NA | NA | NA | NA | NA | NA | NA |
| sq\_Ratio\_SPECIES | EPFU | 0.0000 | 0.0000 | 0 | 0.0000 | NA | NA | NA | NA | NA | NA | NA | NA | 0.0001 | 0.0043 | 0.0000 | 0.0001 | 0.0001 | 0.0001 | 0.0001 | 0.0001 | NA | NA | NA | NA | NA | NA | NA | NA | NA | NA | NA | NA | NA | NA | NA | NA | NA | NA | NA | 0.0128 | 0.0001 | 0.0000 | 0.0101 | 0.0100 | 0.0101 | 0.0101 | 0.0100 |
| INT\_sq\_Ratio\_SPECIES | EPFU | 0.0000 | 0.0000 | 0 | 0.0000 | NA | NA | NA | NA | NA | NA | NA | NA | 0.0002 | 0.0000 | 0.0000 | 0.0001 | 0.0001 | 0.0001 | 0.0001 | 0.0001 | NA | NA | NA | NA | NA | NA | NA | NA | NA | NA | 0.2722 | 0.0000 | 0.0000 | 0.2286 | 0.2263 | 0.2277 | 0.2277 | 0.2265 | NA | 0.0841 | 0.4915 | 0.2474 | 0.0822 | 0.0821 | 0.0821 | 0.0821 | 0.0821 |
| Ratio\_Bats | LABO | 0.0000 | 0.0000 | 0 | 0.0000 | NA | NA | NA | NA | NA | NA | NA | NA | NA | NA | NA | NA | NA | NA | NA | NA | NA | NA | NA | NA | NA | NA | NA | NA | NA | NA | NA | NA | NA | NA | NA | NA | NA | NA | NA | NA | NA | NA | NA | NA | NA | NA | NA |
| log\_Ratio\_Bats | LABO | 0.0000 | 0.0000 | 0 | 0.0000 | 0 | NA | NA | NA | NA | NA | NA | NA | NA | NA | NA | NA | NA | NA | NA | NA | NA | NA | NA | NA | NA | NA | NA | NA | NA | NA | NA | NA | NA | NA | NA | NA | NA | NA | NA | NA | NA | NA | NA | NA | NA | NA | NA |
| INT\_log\_Ratio\_Bats | LABO | 0.0000 | 0.0000 | 0 | 0.0000 | 0 | 0.0000 | NA | NA | NA | NA | NA | NA | NA | NA | NA | NA | NA | NA | NA | NA | NA | NA | NA | NA | NA | NA | NA | NA | NA | NA | NA | NA | NA | NA | NA | NA | NA | NA | NA | NA | NA | NA | NA | NA | NA | NA | NA |
| sq\_Ratio\_Bats | LABO | 0.0000 | 0.0000 | 0 | 0.0000 | NA | NA | 0 | NA | NA | NA | NA | NA | NA | NA | NA | NA | NA | NA | NA | NA | NA | NA | NA | NA | NA | NA | NA | NA | NA | NA | NA | NA | NA | NA | NA | NA | NA | NA | NA | NA | NA | NA | NA | NA | NA | NA | NA |
| INT\_sq\_Ratio\_Bats | LABO | 0.0000 | 0.0000 | 0 | 0.0000 | NA | 0.0000 | 0 | NA | NA | NA | NA | NA | NA | NA | NA | NA | NA | NA | NA | NA | NA | NA | NA | NA | NA | NA | NA | NA | NA | NA | NA | NA | NA | NA | NA | NA | NA | NA | NA | NA | NA | NA | NA | NA | NA | NA | NA |
| Ratio\_High/Low | LABO | 0.0000 | 0.0000 | 0 | 0.0000 | NA | NA | NA | 0.0000 | NA | NA | NA | NA | NA | NA | NA | NA | NA | NA | NA | NA | NA | NA | NA | NA | NA | NA | NA | NA | NA | NA | NA | NA | NA | NA | NA | NA | NA | NA | NA | NA | NA | NA | NA | NA | NA | NA | NA |
| log\_Ratio\_High/Low | LABO | 0.0000 | 0.0000 | 0 | 0.0000 | NA | NA | NA | 0.0000 | 0e+00 | NA | NA | NA | NA | NA | NA | NA | NA | NA | NA | NA | NA | NA | NA | NA | NA | NA | NA | NA | NA | NA | NA | NA | NA | NA | NA | NA | NA | NA | NA | NA | NA | NA | NA | NA | NA | NA | NA |
| INT\_log\_Ratio\_High/Low | LABO | 0.0000 | 0.0000 | 0 | 0.0000 | NA | NA | NA | 0.0000 | 0e+00 | 0 | NA | NA | NA | NA | NA | NA | NA | NA | NA | NA | NA | NA | NA | NA | NA | NA | NA | NA | NA | NA | NA | NA | NA | NA | NA | NA | NA | NA | NA | NA | NA | NA | NA | NA | NA | NA | NA |
| sq\_Ratio\_High/Low | LABO | 0.0000 | 0.0000 | 0 | 0.8518 | NA | NA | NA | 0.0000 | NA | NA | 0 | NA | NA | NA | NA | NA | NA | NA | NA | NA | NA | NA | NA | NA | NA | NA | NA | NA | NA | NA | NA | NA | NA | NA | NA | NA | NA | NA | NA | NA | NA | NA | NA | NA | NA | NA | NA |
| INT\_sq\_Ratio\_High/Low | LABO | 0.0000 | 0.0000 | 0 | 0.2107 | NA | NA | NA | 0.0000 | NA | 0 | 0 | NA | NA | NA | NA | NA | NA | NA | NA | NA | NA | NA | NA | NA | NA | NA | NA | NA | NA | NA | NA | NA | NA | NA | NA | NA | NA | NA | NA | NA | NA | NA | NA | NA | NA | NA | NA |
| Ratio\_SPECIES | LABO | 0.0000 | 0.0000 | 0 | 0.0000 | NA | NA | NA | NA | NA | NA | NA | 0.0208 | NA | 0.0208 | 0.0208 | 0.0193 | 0.1260 | 0.0175 | 0.0116 | 0.5740 | NA | NA | NA | NA | NA | NA | NA | NA | NA | NA | NA | NA | NA | NA | NA | NA | NA | NA | NA | NA | NA | NA | NA | NA | NA | NA | NA |
| log\_Ratio\_SPECIES | LABO | 0.0000 | 0.0000 | 0 | 0.0000 | NA | NA | NA | NA | NA | NA | NA | 0.0000 | NA | 0.0000 | 0.0000 | 0.0000 | 0.0000 | 0.0000 | 0.0000 | 0.0000 | 0.6468 | NA | 0.6456 | 0.6437 | 0.9854 | 0.0000 | 0.5519 | 0.0041 | 0.0000 | NA | NA | NA | NA | NA | NA | NA | NA | NA | NA | NA | NA | NA | NA | NA | NA | NA | NA |
| INT\_log\_Ratio\_SPECIES | LABO | 0.0000 | 0.0000 | 0 | 0.0000 | NA | NA | NA | NA | NA | NA | NA | 0.2018 | NA | 0.2020 | 0.2023 | 0.1496 | 0.0000 | 0.1051 | 0.0202 | 0.0000 | 0.6903 | NA | 0.6891 | 0.6872 | 0.9468 | 0.0000 | 0.5348 | 0.0051 | 0.0000 | 0.8909 | NA | 0.8906 | 0.8903 | 0.9552 | 0.0000 | 0.9816 | 0.7433 | 0.0002 | NA | NA | NA | NA | NA | NA | NA | NA | NA |
| sq\_Ratio\_SPECIES | LABO | 0.0000 | 0.0000 | 0 | 0.0000 | NA | NA | NA | NA | NA | NA | NA | 0.0308 | NA | 0.0308 | 0.0309 | 0.0260 | 0.0000 | 0.0206 | 0.0076 | 0.0394 | NA | NA | NA | NA | NA | NA | NA | NA | NA | NA | NA | NA | NA | NA | NA | NA | NA | NA | 0.1464 | NA | 0.1465 | 0.1465 | 0.1296 | 0.0000 | 0.1097 | 0.0532 | 0.0117 |
| INT\_sq\_Ratio\_SPECIES | LABO | 0.0000 | 0.0000 | 0 | 0.0000 | NA | NA | NA | NA | NA | NA | NA | 0.0163 | NA | 0.0164 | 0.0164 | 0.0120 | 0.0000 | 0.0079 | 0.0012 | 0.0000 | NA | NA | NA | NA | NA | NA | NA | NA | NA | 0.2836 | NA | 0.2837 | 0.2839 | 0.2465 | 0.0000 | 0.2032 | 0.0847 | 0.0000 | 0.4606 | NA | 0.4606 | 0.4606 | 0.4572 | 0.7400 | 0.4524 | 0.4334 | 0.7444 |
| Ratio\_Bats | LACI | 0.0000 | 0.0000 | 0 | 0.0000 | NA | NA | NA | NA | NA | NA | NA | NA | NA | NA | NA | NA | NA | NA | NA | NA | NA | NA | NA | NA | NA | NA | NA | NA | NA | NA | NA | NA | NA | NA | NA | NA | NA | NA | NA | NA | NA | NA | NA | NA | NA | NA | NA |
| log\_Ratio\_Bats | LACI | 0.0000 | 0.0000 | 0 | 0.0000 | 0 | NA | NA | NA | NA | NA | NA | NA | NA | NA | NA | NA | NA | NA | NA | NA | NA | NA | NA | NA | NA | NA | NA | NA | NA | NA | NA | NA | NA | NA | NA | NA | NA | NA | NA | NA | NA | NA | NA | NA | NA | NA | NA |
| INT\_log\_Ratio\_Bats | LACI | 0.0000 | 0.0000 | 0 | 0.0000 | 0 | 0.0000 | NA | NA | NA | NA | NA | NA | NA | NA | NA | NA | NA | NA | NA | NA | NA | NA | NA | NA | NA | NA | NA | NA | NA | NA | NA | NA | NA | NA | NA | NA | NA | NA | NA | NA | NA | NA | NA | NA | NA | NA | NA |
| sq\_Ratio\_Bats | LACI | 0.0000 | 0.0000 | 0 | 0.0000 | NA | NA | 0 | NA | NA | NA | NA | NA | NA | NA | NA | NA | NA | NA | NA | NA | NA | NA | NA | NA | NA | NA | NA | NA | NA | NA | NA | NA | NA | NA | NA | NA | NA | NA | NA | NA | NA | NA | NA | NA | NA | NA | NA |
| INT\_sq\_Ratio\_Bats | LACI | 0.0000 | 0.0000 | 0 | 0.0000 | NA | 0.0000 | 0 | NA | NA | NA | NA | NA | NA | NA | NA | NA | NA | NA | NA | NA | NA | NA | NA | NA | NA | NA | NA | NA | NA | NA | NA | NA | NA | NA | NA | NA | NA | NA | NA | NA | NA | NA | NA | NA | NA | NA | NA |
| Ratio\_High/Low | LACI | 0.0000 | 0.0000 | 0 | 0.0000 | NA | NA | NA | 0.0000 | NA | NA | NA | NA | NA | NA | NA | NA | NA | NA | NA | NA | NA | NA | NA | NA | NA | NA | NA | NA | NA | NA | NA | NA | NA | NA | NA | NA | NA | NA | NA | NA | NA | NA | NA | NA | NA | NA | NA |
| log\_Ratio\_High/Low | LACI | 0.0000 | 0.0000 | 0 | 0.0000 | NA | NA | NA | 0.0000 | 0e+00 | NA | NA | NA | NA | NA | NA | NA | NA | NA | NA | NA | NA | NA | NA | NA | NA | NA | NA | NA | NA | NA | NA | NA | NA | NA | NA | NA | NA | NA | NA | NA | NA | NA | NA | NA | NA | NA | NA |
| INT\_log\_Ratio\_High/Low | LACI | 0.0000 | 0.0000 | 0 | 0.0000 | NA | NA | NA | 0.0000 | 0e+00 | 0 | NA | NA | NA | NA | NA | NA | NA | NA | NA | NA | NA | NA | NA | NA | NA | NA | NA | NA | NA | NA | NA | NA | NA | NA | NA | NA | NA | NA | NA | NA | NA | NA | NA | NA | NA | NA | NA |
| sq\_Ratio\_High/Low | LACI | 0.0000 | 0.0000 | 0 | 0.0000 | NA | NA | NA | 0.0000 | NA | NA | 0 | NA | NA | NA | NA | NA | NA | NA | NA | NA | NA | NA | NA | NA | NA | NA | NA | NA | NA | NA | NA | NA | NA | NA | NA | NA | NA | NA | NA | NA | NA | NA | NA | NA | NA | NA | NA |
| INT\_sq\_Ratio\_High/Low | LACI | 0.0000 | 0.0000 | 0 | 0.0000 | NA | NA | NA | 0.0000 | NA | 0 | 0 | NA | NA | NA | NA | NA | NA | NA | NA | NA | NA | NA | NA | NA | NA | NA | NA | NA | NA | NA | NA | NA | NA | NA | NA | NA | NA | NA | NA | NA | NA | NA | NA | NA | NA | NA | NA |
| Ratio\_SPECIES | LACI | 0.0000 | 0.0000 | 0 | 0.0000 | NA | NA | NA | NA | NA | NA | NA | 0.2683 | 0.0009 | NA | 0.6817 | 0.0009 | 0.0009 | 0.0009 | 0.0009 | 0.0009 | NA | NA | NA | NA | NA | NA | NA | NA | NA | NA | NA | NA | NA | NA | NA | NA | NA | NA | NA | NA | NA | NA | NA | NA | NA | NA | NA |
| log\_Ratio\_SPECIES | LACI | 0.0000 | 0.0000 | 0 | 0.0000 | NA | NA | NA | NA | NA | NA | NA | 0.0000 | 0.0000 | NA | 0.0000 | 0.0000 | 0.0000 | 0.0000 | 0.0000 | 0.0000 | 0.0000 | 0.0210 | NA | 0.0000 | 0.0239 | 0.0240 | 0.0232 | 0.0232 | 0.0231 | NA | NA | NA | NA | NA | NA | NA | NA | NA | NA | NA | NA | NA | NA | NA | NA | NA | NA |
| INT\_log\_Ratio\_SPECIES | LACI | 0.0000 | 0.0000 | 0 | 0.0000 | NA | NA | NA | NA | NA | NA | NA | 0.0000 | 0.0437 | NA | 0.0000 | 0.0452 | 0.0453 | 0.0449 | 0.0449 | 0.0448 | 0.0000 | 0.0260 | NA | 0.0000 | 0.0293 | 0.0295 | 0.0285 | 0.0285 | 0.0284 | 0.0000 | 0.8381 | NA | 0.0000 | 0.8431 | 0.8434 | 0.8424 | 0.8424 | 0.8421 | NA | NA | NA | NA | NA | NA | NA | NA | NA |
| sq\_Ratio\_SPECIES | LACI | 0.0000 | 0.0000 | 0 | 0.0000 | NA | NA | NA | NA | NA | NA | NA | 0.0000 | 0.0006 | NA | 0.0003 | 0.0006 | 0.0006 | 0.0006 | 0.0006 | 0.0006 | NA | NA | NA | NA | NA | NA | NA | NA | NA | NA | NA | NA | NA | NA | NA | NA | NA | NA | 0.0000 | 0.0225 | NA | 0.0000 | 0.0228 | 0.0229 | 0.0227 | 0.0227 | 0.0227 |
| INT\_sq\_Ratio\_SPECIES | LACI | 0.0000 | 0.0000 | 0 | 0.0000 | NA | NA | NA | NA | NA | NA | NA | 0.0000 | 0.0003 | NA | 0.0000 | 0.0003 | 0.0003 | 0.0003 | 0.0003 | 0.0003 | NA | NA | NA | NA | NA | NA | NA | NA | NA | 0.0000 | 0.1537 | NA | 0.0000 | 0.1563 | 0.1564 | 0.1556 | 0.1555 | 0.1555 | 0.8024 | 0.1950 | NA | 0.6756 | 0.1953 | 0.1953 | 0.1952 | 0.1952 | 0.1952 |
| Ratio\_Bats | LANO | 0.0000 | 0.0000 | 0 | 0.0000 | NA | NA | NA | NA | NA | NA | NA | NA | NA | NA | NA | NA | NA | NA | NA | NA | NA | NA | NA | NA | NA | NA | NA | NA | NA | NA | NA | NA | NA | NA | NA | NA | NA | NA | NA | NA | NA | NA | NA | NA | NA | NA | NA |
| log\_Ratio\_Bats | LANO | 0.0002 | 0.4875 | 0 | 0.0000 | 0 | NA | NA | NA | NA | NA | NA | NA | NA | NA | NA | NA | NA | NA | NA | NA | NA | NA | NA | NA | NA | NA | NA | NA | NA | NA | NA | NA | NA | NA | NA | NA | NA | NA | NA | NA | NA | NA | NA | NA | NA | NA | NA |
| INT\_log\_Ratio\_Bats | LANO | 0.0000 | 0.0000 | 0 | 0.0000 | 0 | 0.0000 | NA | NA | NA | NA | NA | NA | NA | NA | NA | NA | NA | NA | NA | NA | NA | NA | NA | NA | NA | NA | NA | NA | NA | NA | NA | NA | NA | NA | NA | NA | NA | NA | NA | NA | NA | NA | NA | NA | NA | NA | NA |
| sq\_Ratio\_Bats | LANO | 0.0000 | 0.0001 | 0 | 0.0000 | NA | NA | 0 | NA | NA | NA | NA | NA | NA | NA | NA | NA | NA | NA | NA | NA | NA | NA | NA | NA | NA | NA | NA | NA | NA | NA | NA | NA | NA | NA | NA | NA | NA | NA | NA | NA | NA | NA | NA | NA | NA | NA | NA |
| INT\_sq\_Ratio\_Bats | LANO | 0.0000 | 0.0000 | 0 | 0.0000 | NA | 0.0000 | 0 | NA | NA | NA | NA | NA | NA | NA | NA | NA | NA | NA | NA | NA | NA | NA | NA | NA | NA | NA | NA | NA | NA | NA | NA | NA | NA | NA | NA | NA | NA | NA | NA | NA | NA | NA | NA | NA | NA | NA | NA |
| Ratio\_High/Low | LANO | 0.0000 | 0.0000 | 0 | 0.0000 | NA | NA | NA | 0.0000 | NA | NA | NA | NA | NA | NA | NA | NA | NA | NA | NA | NA | NA | NA | NA | NA | NA | NA | NA | NA | NA | NA | NA | NA | NA | NA | NA | NA | NA | NA | NA | NA | NA | NA | NA | NA | NA | NA | NA |
| log\_Ratio\_High/Low | LANO | 0.0000 | 0.0000 | 0 | 0.0000 | NA | NA | NA | 0.0000 | 2e-04 | NA | NA | NA | NA | NA | NA | NA | NA | NA | NA | NA | NA | NA | NA | NA | NA | NA | NA | NA | NA | NA | NA | NA | NA | NA | NA | NA | NA | NA | NA | NA | NA | NA | NA | NA | NA | NA | NA |
| INT\_log\_Ratio\_High/Low | LANO | 0.0000 | 0.0000 | 0 | 0.0000 | NA | NA | NA | 0.0000 | 0e+00 | 0 | NA | NA | NA | NA | NA | NA | NA | NA | NA | NA | NA | NA | NA | NA | NA | NA | NA | NA | NA | NA | NA | NA | NA | NA | NA | NA | NA | NA | NA | NA | NA | NA | NA | NA | NA | NA | NA |
| sq\_Ratio\_High/Low | LANO | 0.0000 | 0.0000 | 0 | 0.0000 | NA | NA | NA | 0.0000 | NA | NA | 0 | NA | NA | NA | NA | NA | NA | NA | NA | NA | NA | NA | NA | NA | NA | NA | NA | NA | NA | NA | NA | NA | NA | NA | NA | NA | NA | NA | NA | NA | NA | NA | NA | NA | NA | NA | NA |
| INT\_sq\_Ratio\_High/Low | LANO | 0.0000 | 0.0000 | 0 | 0.0604 | NA | NA | NA | 0.0000 | NA | 0 | 0 | NA | NA | NA | NA | NA | NA | NA | NA | NA | NA | NA | NA | NA | NA | NA | NA | NA | NA | NA | NA | NA | NA | NA | NA | NA | NA | NA | NA | NA | NA | NA | NA | NA | NA | NA | NA |
| Ratio\_SPECIES | LANO | 0.0000 | 0.0000 | 0 | 0.0000 | NA | NA | NA | NA | NA | NA | NA | 0.0987 | 0.0000 | 0.2165 | NA | 0.0000 | 0.0000 | 0.0000 | 0.0000 | 0.0000 | NA | NA | NA | NA | NA | NA | NA | NA | NA | NA | NA | NA | NA | NA | NA | NA | NA | NA | NA | NA | NA | NA | NA | NA | NA | NA | NA |
| log\_Ratio\_SPECIES | LANO | 0.0000 | 0.0000 | 0 | 0.0004 | NA | NA | NA | NA | NA | NA | NA | 0.0000 | 0.0012 | 0.0000 | NA | 0.0014 | 0.0015 | 0.0014 | 0.0014 | 0.0014 | 0.0000 | 0.0001 | 0.0000 | NA | 0.0002 | 0.0002 | 0.0002 | 0.0002 | 0.0002 | NA | NA | NA | NA | NA | NA | NA | NA | NA | NA | NA | NA | NA | NA | NA | NA | NA | NA |
| INT\_log\_Ratio\_SPECIES | LANO | 0.0000 | 0.4697 | 0 | 0.0000 | NA | NA | NA | NA | NA | NA | NA | 0.0035 | 0.0958 | 0.0041 | NA | 0.0967 | 0.1031 | 0.1005 | 0.1014 | 0.1011 | 0.0000 | 0.0003 | 0.0000 | NA | 0.0003 | 0.0003 | 0.0003 | 0.0003 | 0.0003 | 0.0021 | 0.4711 | 0.0052 | NA | 0.4665 | 0.4848 | 0.4795 | 0.4830 | 0.4827 | NA | NA | NA | NA | NA | NA | NA | NA | NA |
| sq\_Ratio\_SPECIES | LANO | 0.0000 | 0.0000 | 0 | 0.0000 | NA | NA | NA | NA | NA | NA | NA | 0.0000 | 0.0001 | 0.0000 | NA | 0.0001 | 0.0001 | 0.0001 | 0.0001 | 0.0001 | NA | NA | NA | NA | NA | NA | NA | NA | NA | NA | NA | NA | NA | NA | NA | NA | NA | NA | 0.0000 | 0.0101 | 0.0000 | NA | 0.0109 | 0.0104 | 0.0104 | 0.0102 | 0.0102 |
| INT\_sq\_Ratio\_SPECIES | LANO | 0.0000 | 0.0000 | 0 | 0.0000 | NA | NA | NA | NA | NA | NA | NA | 0.0000 | 0.0003 | 0.0000 | NA | 0.0003 | 0.0003 | 0.0003 | 0.0003 | 0.0003 | NA | NA | NA | NA | NA | NA | NA | NA | NA | 0.0000 | 0.4633 | 0.0000 | NA | 0.4782 | 0.4682 | 0.4665 | 0.4637 | 0.4627 | 0.0080 | 0.0353 | 0.0247 | NA | 0.0361 | 0.0355 | 0.0358 | 0.0354 | 0.0354 |
| Ratio\_Bats | MYLE | 0.0000 | 0.0000 | 0 | 0.0000 | NA | NA | NA | NA | NA | NA | NA | NA | NA | NA | NA | NA | NA | NA | NA | NA | NA | NA | NA | NA | NA | NA | NA | NA | NA | NA | NA | NA | NA | NA | NA | NA | NA | NA | NA | NA | NA | NA | NA | NA | NA | NA | NA |
| log\_Ratio\_Bats | MYLE | 0.0000 | 0.0000 | 0 | 0.0000 | 0 | NA | NA | NA | NA | NA | NA | NA | NA | NA | NA | NA | NA | NA | NA | NA | NA | NA | NA | NA | NA | NA | NA | NA | NA | NA | NA | NA | NA | NA | NA | NA | NA | NA | NA | NA | NA | NA | NA | NA | NA | NA | NA |
| INT\_log\_Ratio\_Bats | MYLE | 0.0000 | 0.0000 | 0 | 0.0000 | 0 | 0.0000 | NA | NA | NA | NA | NA | NA | NA | NA | NA | NA | NA | NA | NA | NA | NA | NA | NA | NA | NA | NA | NA | NA | NA | NA | NA | NA | NA | NA | NA | NA | NA | NA | NA | NA | NA | NA | NA | NA | NA | NA | NA |
| sq\_Ratio\_Bats | MYLE | 0.0000 | 0.0000 | 0 | 0.0000 | NA | NA | 0 | NA | NA | NA | NA | NA | NA | NA | NA | NA | NA | NA | NA | NA | NA | NA | NA | NA | NA | NA | NA | NA | NA | NA | NA | NA | NA | NA | NA | NA | NA | NA | NA | NA | NA | NA | NA | NA | NA | NA | NA |
| INT\_sq\_Ratio\_Bats | MYLE | 0.0000 | 0.0000 | 0 | 0.0000 | NA | 0.0000 | 0 | NA | NA | NA | NA | NA | NA | NA | NA | NA | NA | NA | NA | NA | NA | NA | NA | NA | NA | NA | NA | NA | NA | NA | NA | NA | NA | NA | NA | NA | NA | NA | NA | NA | NA | NA | NA | NA | NA | NA | NA |
| Ratio\_High/Low | MYLE | 0.0000 | 0.0000 | 0 | 0.0000 | NA | NA | NA | 0.0000 | NA | NA | NA | NA | NA | NA | NA | NA | NA | NA | NA | NA | NA | NA | NA | NA | NA | NA | NA | NA | NA | NA | NA | NA | NA | NA | NA | NA | NA | NA | NA | NA | NA | NA | NA | NA | NA | NA | NA |
| log\_Ratio\_High/Low | MYLE | 0.0000 | 0.0000 | 0 | 0.0000 | NA | NA | NA | 0.0000 | 0e+00 | NA | NA | NA | NA | NA | NA | NA | NA | NA | NA | NA | NA | NA | NA | NA | NA | NA | NA | NA | NA | NA | NA | NA | NA | NA | NA | NA | NA | NA | NA | NA | NA | NA | NA | NA | NA | NA | NA |
| INT\_log\_Ratio\_High/Low | MYLE | 0.0000 | 0.0000 | 0 | 0.0000 | NA | NA | NA | 0.0000 | 0e+00 | 0 | NA | NA | NA | NA | NA | NA | NA | NA | NA | NA | NA | NA | NA | NA | NA | NA | NA | NA | NA | NA | NA | NA | NA | NA | NA | NA | NA | NA | NA | NA | NA | NA | NA | NA | NA | NA | NA |
| sq\_Ratio\_High/Low | MYLE | 0.0000 | 0.0000 | 0 | 0.3784 | NA | NA | NA | 0.0000 | NA | NA | 0 | NA | NA | NA | NA | NA | NA | NA | NA | NA | NA | NA | NA | NA | NA | NA | NA | NA | NA | NA | NA | NA | NA | NA | NA | NA | NA | NA | NA | NA | NA | NA | NA | NA | NA | NA | NA |
| INT\_sq\_Ratio\_High/Low | MYLE | 0.0000 | 0.0000 | 0 | 0.3639 | NA | NA | NA | 0.0000 | NA | 0 | 0 | NA | NA | NA | NA | NA | NA | NA | NA | NA | NA | NA | NA | NA | NA | NA | NA | NA | NA | NA | NA | NA | NA | NA | NA | NA | NA | NA | NA | NA | NA | NA | NA | NA | NA | NA | NA |
| Ratio\_SPECIES | MYLE | 0.0000 | 0.0000 | 0 | 0.0000 | NA | NA | NA | NA | NA | NA | NA | 0.0025 | 0.0029 | 0.0025 | 0.0025 | NA | 0.0083 | 0.5754 | 0.3118 | 0.0020 | NA | NA | NA | NA | NA | NA | NA | NA | NA | NA | NA | NA | NA | NA | NA | NA | NA | NA | NA | NA | NA | NA | NA | NA | NA | NA | NA |
| log\_Ratio\_SPECIES | MYLE | 0.0000 | 0.0000 | 0 | 0.0000 | NA | NA | NA | NA | NA | NA | NA | 0.0000 | 0.0000 | 0.0000 | 0.0000 | NA | 0.0337 | 0.0000 | 0.0000 | 0.0000 | 0.0298 | 0.1601 | 0.0298 | 0.0298 | NA | 0.0000 | 0.0000 | 0.0000 | 0.0017 | NA | NA | NA | NA | NA | NA | NA | NA | NA | NA | NA | NA | NA | NA | NA | NA | NA | NA |
| INT\_log\_Ratio\_SPECIES | MYLE | 0.0000 | 0.0000 | 0 | 0.0000 | NA | NA | NA | NA | NA | NA | NA | 0.0521 | 0.0870 | 0.0521 | 0.0521 | NA | 0.6654 | 0.0000 | 0.0000 | 0.0244 | 0.0356 | 0.1711 | 0.0355 | 0.0355 | NA | 0.0000 | 0.0000 | 0.0000 | 0.0024 | 0.8664 | 0.9434 | 0.8664 | 0.8664 | NA | 0.3160 | 0.0000 | 0.0000 | 0.7588 | NA | NA | NA | NA | NA | NA | NA | NA | NA |
| sq\_Ratio\_SPECIES | MYLE | 0.0000 | 0.0000 | 0 | 0.0000 | NA | NA | NA | NA | NA | NA | NA | 0.0011 | 0.0017 | 0.0011 | 0.0011 | NA | 0.0210 | 0.0069 | 0.0000 | 0.0007 | NA | NA | NA | NA | NA | NA | NA | NA | NA | NA | NA | NA | NA | NA | NA | NA | NA | NA | 0.0290 | 0.0384 | 0.0290 | 0.0290 | NA | 0.1960 | 0.0006 | 0.0000 | 0.0203 |
| INT\_sq\_Ratio\_SPECIES | MYLE | 0.0000 | 0.0000 | 0 | 0.0000 | NA | NA | NA | NA | NA | NA | NA | 0.0005 | 0.0010 | 0.0005 | 0.0005 | NA | 0.0544 | 0.0000 | 0.0000 | 0.0002 | NA | NA | NA | NA | NA | NA | NA | NA | NA | 0.1351 | 0.1794 | 0.1351 | 0.1351 | NA | 0.7852 | 0.0000 | 0.0000 | 0.0932 | 0.2522 | 0.2571 | 0.2522 | 0.2522 | NA | 0.2862 | 0.6273 | 0.9205 | 0.2467 |
| Ratio\_Bats | MYLU | 0.0000 | 0.0000 | 0 | 0.0000 | NA | NA | NA | NA | NA | NA | NA | NA | NA | NA | NA | NA | NA | NA | NA | NA | NA | NA | NA | NA | NA | NA | NA | NA | NA | NA | NA | NA | NA | NA | NA | NA | NA | NA | NA | NA | NA | NA | NA | NA | NA | NA | NA |
| log\_Ratio\_Bats | MYLU | 0.0000 | 0.0000 | 0 | 0.0000 | 0 | NA | NA | NA | NA | NA | NA | NA | NA | NA | NA | NA | NA | NA | NA | NA | NA | NA | NA | NA | NA | NA | NA | NA | NA | NA | NA | NA | NA | NA | NA | NA | NA | NA | NA | NA | NA | NA | NA | NA | NA | NA | NA |
| INT\_log\_Ratio\_Bats | MYLU | 0.0000 | 0.0000 | 0 | 0.3320 | 0 | 0.0000 | NA | NA | NA | NA | NA | NA | NA | NA | NA | NA | NA | NA | NA | NA | NA | NA | NA | NA | NA | NA | NA | NA | NA | NA | NA | NA | NA | NA | NA | NA | NA | NA | NA | NA | NA | NA | NA | NA | NA | NA | NA |
| sq\_Ratio\_Bats | MYLU | 0.0000 | 0.0000 | 0 | 0.0000 | NA | NA | 0 | NA | NA | NA | NA | NA | NA | NA | NA | NA | NA | NA | NA | NA | NA | NA | NA | NA | NA | NA | NA | NA | NA | NA | NA | NA | NA | NA | NA | NA | NA | NA | NA | NA | NA | NA | NA | NA | NA | NA | NA |
| INT\_sq\_Ratio\_Bats | MYLU | 0.0000 | 0.0001 | 0 | 0.0000 | NA | 0.0000 | 0 | NA | NA | NA | NA | NA | NA | NA | NA | NA | NA | NA | NA | NA | NA | NA | NA | NA | NA | NA | NA | NA | NA | NA | NA | NA | NA | NA | NA | NA | NA | NA | NA | NA | NA | NA | NA | NA | NA | NA | NA |
| Ratio\_High/Low | MYLU | 0.0000 | 0.0000 | 0 | 0.0000 | NA | NA | NA | 0.0000 | NA | NA | NA | NA | NA | NA | NA | NA | NA | NA | NA | NA | NA | NA | NA | NA | NA | NA | NA | NA | NA | NA | NA | NA | NA | NA | NA | NA | NA | NA | NA | NA | NA | NA | NA | NA | NA | NA | NA |
| log\_Ratio\_High/Low | MYLU | 0.0000 | 0.0000 | 0 | 0.0000 | NA | NA | NA | 0.0340 | 0e+00 | NA | NA | NA | NA | NA | NA | NA | NA | NA | NA | NA | NA | NA | NA | NA | NA | NA | NA | NA | NA | NA | NA | NA | NA | NA | NA | NA | NA | NA | NA | NA | NA | NA | NA | NA | NA | NA | NA |
| INT\_log\_Ratio\_High/Low | MYLU | 0.0001 | 0.0075 | 0 | 0.0413 | NA | NA | NA | 0.0000 | 0e+00 | 0 | NA | NA | NA | NA | NA | NA | NA | NA | NA | NA | NA | NA | NA | NA | NA | NA | NA | NA | NA | NA | NA | NA | NA | NA | NA | NA | NA | NA | NA | NA | NA | NA | NA | NA | NA | NA | NA |
| sq\_Ratio\_High/Low | MYLU | 0.0000 | 0.0000 | 0 | 0.5303 | NA | NA | NA | 0.0000 | NA | NA | 0 | NA | NA | NA | NA | NA | NA | NA | NA | NA | NA | NA | NA | NA | NA | NA | NA | NA | NA | NA | NA | NA | NA | NA | NA | NA | NA | NA | NA | NA | NA | NA | NA | NA | NA | NA | NA |
| INT\_sq\_Ratio\_High/Low | MYLU | 0.0000 | 0.0189 | 0 | 0.0019 | NA | NA | NA | 0.0000 | NA | 0 | 0 | NA | NA | NA | NA | NA | NA | NA | NA | NA | NA | NA | NA | NA | NA | NA | NA | NA | NA | NA | NA | NA | NA | NA | NA | NA | NA | NA | NA | NA | NA | NA | NA | NA | NA | NA | NA |
| Ratio\_SPECIES | MYLU | 0.0000 | 0.0000 | 0 | 0.0000 | NA | NA | NA | NA | NA | NA | NA | 0.0000 | 0.4168 | 0.0000 | 0.0000 | 0.0000 | NA | 0.0000 | 0.0105 | 0.0081 | NA | NA | NA | NA | NA | NA | NA | NA | NA | NA | NA | NA | NA | NA | NA | NA | NA | NA | NA | NA | NA | NA | NA | NA | NA | NA | NA |
| log\_Ratio\_SPECIES | MYLU | 0.0000 | 0.0000 | 0 | 0.0000 | NA | NA | NA | NA | NA | NA | NA | 0.0000 | 0.0000 | 0.0000 | 0.0000 | 0.0000 | NA | 0.5836 | 0.0000 | 0.0000 | 0.0000 | 0.0000 | 0.0000 | 0.0000 | 0.0000 | NA | 0.0177 | 0.0000 | 0.0000 | NA | NA | NA | NA | NA | NA | NA | NA | NA | NA | NA | NA | NA | NA | NA | NA | NA | NA |
| INT\_log\_Ratio\_SPECIES | MYLU | 0.0000 | 0.0000 | 0 | 0.0000 | NA | NA | NA | NA | NA | NA | NA | 0.0067 | 0.0000 | 0.0065 | 0.0067 | 0.0008 | NA | 0.7554 | 0.0000 | 0.0003 | 0.0000 | 0.0000 | 0.0000 | 0.0000 | 0.0000 | NA | 0.0393 | 0.0000 | 0.0000 | 0.3306 | 0.8883 | 0.3267 | 0.3312 | 0.3034 | NA | 0.8850 | 0.4081 | 0.6945 | NA | NA | NA | NA | NA | NA | NA | NA | NA |
| sq\_Ratio\_SPECIES | MYLU | 0.0000 | 0.0000 | 0 | 0.0000 | NA | NA | NA | NA | NA | NA | NA | 0.0000 | 0.0000 | 0.0000 | 0.0000 | 0.0000 | NA | 0.0003 | 0.1854 | 0.3317 | NA | NA | NA | NA | NA | NA | NA | NA | NA | NA | NA | NA | NA | NA | NA | NA | NA | NA | 0.0004 | 0.0000 | 0.0004 | 0.0004 | 0.0000 | NA | 0.0597 | 0.0063 | 0.0175 |
| INT\_sq\_Ratio\_SPECIES | MYLU | 0.0000 | 0.0000 | 0 | 0.0000 | NA | NA | NA | NA | NA | NA | NA | 0.0000 | 0.0000 | 0.0000 | 0.0000 | 0.0000 | NA | 0.0128 | 0.0000 | 0.0000 | NA | NA | NA | NA | NA | NA | NA | NA | NA | 0.0999 | 0.0000 | 0.1006 | 0.0991 | 0.0121 | NA | 0.6777 | 0.0000 | 0.0000 | 0.0169 | 0.1592 | 0.0169 | 0.0168 | 0.0110 | NA | 0.0355 | 0.2941 | 0.2321 |
| Ratio\_Bats | MYSE | 0.0000 | 0.0000 | 0 | 0.0000 | NA | NA | NA | NA | NA | NA | NA | NA | NA | NA | NA | NA | NA | NA | NA | NA | NA | NA | NA | NA | NA | NA | NA | NA | NA | NA | NA | NA | NA | NA | NA | NA | NA | NA | NA | NA | NA | NA | NA | NA | NA | NA | NA |
| log\_Ratio\_Bats | MYSE | 0.0000 | 0.0000 | 0 | 0.0000 | 0 | NA | NA | NA | NA | NA | NA | NA | NA | NA | NA | NA | NA | NA | NA | NA | NA | NA | NA | NA | NA | NA | NA | NA | NA | NA | NA | NA | NA | NA | NA | NA | NA | NA | NA | NA | NA | NA | NA | NA | NA | NA | NA |
| INT\_log\_Ratio\_Bats | MYSE | 0.0000 | 0.0000 | 0 | 0.0000 | 0 | 0.8922 | NA | NA | NA | NA | NA | NA | NA | NA | NA | NA | NA | NA | NA | NA | NA | NA | NA | NA | NA | NA | NA | NA | NA | NA | NA | NA | NA | NA | NA | NA | NA | NA | NA | NA | NA | NA | NA | NA | NA | NA | NA |
| sq\_Ratio\_Bats | MYSE | 0.0000 | 0.0000 | 0 | 0.0000 | NA | NA | 0 | NA | NA | NA | NA | NA | NA | NA | NA | NA | NA | NA | NA | NA | NA | NA | NA | NA | NA | NA | NA | NA | NA | NA | NA | NA | NA | NA | NA | NA | NA | NA | NA | NA | NA | NA | NA | NA | NA | NA | NA |
| INT\_sq\_Ratio\_Bats | MYSE | 0.0000 | 0.0000 | 0 | 0.0000 | NA | 0.0000 | 0 | NA | NA | NA | NA | NA | NA | NA | NA | NA | NA | NA | NA | NA | NA | NA | NA | NA | NA | NA | NA | NA | NA | NA | NA | NA | NA | NA | NA | NA | NA | NA | NA | NA | NA | NA | NA | NA | NA | NA | NA |
| Ratio\_High/Low | MYSE | 0.0000 | 0.0000 | 0 | 0.0000 | NA | NA | NA | 0.0000 | NA | NA | NA | NA | NA | NA | NA | NA | NA | NA | NA | NA | NA | NA | NA | NA | NA | NA | NA | NA | NA | NA | NA | NA | NA | NA | NA | NA | NA | NA | NA | NA | NA | NA | NA | NA | NA | NA | NA |
| log\_Ratio\_High/Low | MYSE | 0.0000 | 0.0000 | 0 | 0.0002 | NA | NA | NA | 0.0000 | 0e+00 | NA | NA | NA | NA | NA | NA | NA | NA | NA | NA | NA | NA | NA | NA | NA | NA | NA | NA | NA | NA | NA | NA | NA | NA | NA | NA | NA | NA | NA | NA | NA | NA | NA | NA | NA | NA | NA | NA |
| INT\_log\_Ratio\_High/Low | MYSE | 0.0000 | 0.0000 | 0 | 0.0000 | NA | NA | NA | 0.0000 | 0e+00 | 0 | NA | NA | NA | NA | NA | NA | NA | NA | NA | NA | NA | NA | NA | NA | NA | NA | NA | NA | NA | NA | NA | NA | NA | NA | NA | NA | NA | NA | NA | NA | NA | NA | NA | NA | NA | NA | NA |
| sq\_Ratio\_High/Low | MYSE | 0.0000 | 0.0000 | 0 | 0.2354 | NA | NA | NA | 0.0000 | NA | NA | 0 | NA | NA | NA | NA | NA | NA | NA | NA | NA | NA | NA | NA | NA | NA | NA | NA | NA | NA | NA | NA | NA | NA | NA | NA | NA | NA | NA | NA | NA | NA | NA | NA | NA | NA | NA | NA |
| INT\_sq\_Ratio\_High/Low | MYSE | 0.0000 | 0.0000 | 0 | 0.2124 | NA | NA | NA | 0.0000 | NA | 0 | 0 | NA | NA | NA | NA | NA | NA | NA | NA | NA | NA | NA | NA | NA | NA | NA | NA | NA | NA | NA | NA | NA | NA | NA | NA | NA | NA | NA | NA | NA | NA | NA | NA | NA | NA | NA | NA |
| Ratio\_SPECIES | MYSE | 0.0000 | 0.0000 | 0 | 0.0000 | NA | NA | NA | NA | NA | NA | NA | 0.0000 | 0.0000 | 0.0000 | 0.0000 | 0.3037 | 0.9889 | NA | 0.0244 | 0.0000 | NA | NA | NA | NA | NA | NA | NA | NA | NA | NA | NA | NA | NA | NA | NA | NA | NA | NA | NA | NA | NA | NA | NA | NA | NA | NA | NA |
| log\_Ratio\_SPECIES | MYSE | 0.0000 | 0.0000 | 0 | 0.0000 | NA | NA | NA | NA | NA | NA | NA | 0.0000 | 0.0000 | 0.0000 | 0.0000 | 0.0000 | 0.0000 | NA | 0.0000 | 0.0000 | 0.0000 | 0.0000 | 0.0000 | 0.0000 | 0.0000 | 0.0000 | NA | 0.0000 | 0.0000 | NA | NA | NA | NA | NA | NA | NA | NA | NA | NA | NA | NA | NA | NA | NA | NA | NA | NA |
| INT\_log\_Ratio\_SPECIES | MYSE | 0.0000 | 0.0000 | 0 | 0.0000 | NA | NA | NA | NA | NA | NA | NA | 0.0044 | 0.0103 | 0.0044 | 0.0044 | 0.0000 | 0.0000 | NA | 0.0000 | 0.0008 | 0.0000 | 0.0000 | 0.0000 | 0.0000 | 0.0000 | 0.0000 | NA | 0.0000 | 0.0000 | 0.5056 | 0.7080 | 0.5053 | 0.5056 | 0.0007 | 0.0002 | NA | 0.0091 | 0.3898 | NA | NA | NA | NA | NA | NA | NA | NA | NA |
| sq\_Ratio\_SPECIES | MYSE | 0.0000 | 0.0000 | 0 | 0.0000 | NA | NA | NA | NA | NA | NA | NA | 0.0000 | 0.0000 | 0.0000 | 0.0000 | 0.0013 | 0.0000 | NA | 0.7799 | 0.0000 | NA | NA | NA | NA | NA | NA | NA | NA | NA | NA | NA | NA | NA | NA | NA | NA | NA | NA | 0.0010 | 0.0006 | 0.0010 | 0.0009 | 0.0000 | 0.0000 | NA | 0.1020 | 0.0004 |
| INT\_sq\_Ratio\_SPECIES | MYSE | 0.0000 | 0.0000 | 0 | 0.0000 | NA | NA | NA | NA | NA | NA | NA | 0.0000 | 0.0000 | 0.0000 | 0.0000 | 0.0000 | 0.0000 | NA | 0.0011 | 0.0000 | NA | NA | NA | NA | NA | NA | NA | NA | NA | 0.0509 | 0.0362 | 0.0509 | 0.0508 | 0.0000 | 0.0000 | NA | 0.0000 | 0.0219 | 0.0545 | 0.0498 | 0.0545 | 0.0545 | 0.4953 | 0.8976 | NA | 0.2216 | 0.0506 |
| Ratio\_Bats | MYSO | 0.0000 | 0.0000 | 0 | 0.0000 | NA | NA | NA | NA | NA | NA | NA | NA | NA | NA | NA | NA | NA | NA | NA | NA | NA | NA | NA | NA | NA | NA | NA | NA | NA | NA | NA | NA | NA | NA | NA | NA | NA | NA | NA | NA | NA | NA | NA | NA | NA | NA | NA |
| log\_Ratio\_Bats | MYSO | 0.0000 | 0.0000 | 0 | 0.0000 | 0 | NA | NA | NA | NA | NA | NA | NA | NA | NA | NA | NA | NA | NA | NA | NA | NA | NA | NA | NA | NA | NA | NA | NA | NA | NA | NA | NA | NA | NA | NA | NA | NA | NA | NA | NA | NA | NA | NA | NA | NA | NA | NA |
| INT\_log\_Ratio\_Bats | MYSO | 0.6158 | 0.6319 | 0 | 0.0161 | 0 | 0.0000 | NA | NA | NA | NA | NA | NA | NA | NA | NA | NA | NA | NA | NA | NA | NA | NA | NA | NA | NA | NA | NA | NA | NA | NA | NA | NA | NA | NA | NA | NA | NA | NA | NA | NA | NA | NA | NA | NA | NA | NA | NA |
| sq\_Ratio\_Bats | MYSO | 0.0000 | 0.0000 | 0 | 0.0000 | NA | NA | 0 | NA | NA | NA | NA | NA | NA | NA | NA | NA | NA | NA | NA | NA | NA | NA | NA | NA | NA | NA | NA | NA | NA | NA | NA | NA | NA | NA | NA | NA | NA | NA | NA | NA | NA | NA | NA | NA | NA | NA | NA |
| INT\_sq\_Ratio\_Bats | MYSO | 0.0000 | 0.8497 | 0 | 0.0000 | NA | 0.0000 | 0 | NA | NA | NA | NA | NA | NA | NA | NA | NA | NA | NA | NA | NA | NA | NA | NA | NA | NA | NA | NA | NA | NA | NA | NA | NA | NA | NA | NA | NA | NA | NA | NA | NA | NA | NA | NA | NA | NA | NA | NA |
| Ratio\_High/Low | MYSO | 0.0000 | 0.0000 | 0 | 0.0000 | NA | NA | NA | 0.0000 | NA | NA | NA | NA | NA | NA | NA | NA | NA | NA | NA | NA | NA | NA | NA | NA | NA | NA | NA | NA | NA | NA | NA | NA | NA | NA | NA | NA | NA | NA | NA | NA | NA | NA | NA | NA | NA | NA | NA |
| log\_Ratio\_High/Low | MYSO | 0.6947 | 0.0000 | 0 | 0.0000 | NA | NA | NA | 0.0109 | 0e+00 | NA | NA | NA | NA | NA | NA | NA | NA | NA | NA | NA | NA | NA | NA | NA | NA | NA | NA | NA | NA | NA | NA | NA | NA | NA | NA | NA | NA | NA | NA | NA | NA | NA | NA | NA | NA | NA | NA |
| INT\_log\_Ratio\_High/Low | MYSO | 0.0000 | 0.0101 | 0 | 0.0000 | NA | NA | NA | 0.0000 | 0e+00 | 0 | NA | NA | NA | NA | NA | NA | NA | NA | NA | NA | NA | NA | NA | NA | NA | NA | NA | NA | NA | NA | NA | NA | NA | NA | NA | NA | NA | NA | NA | NA | NA | NA | NA | NA | NA | NA | NA |
| sq\_Ratio\_High/Low | MYSO | 0.0000 | 0.0000 | 0 | 0.7376 | NA | NA | NA | 0.0000 | NA | NA | 0 | NA | NA | NA | NA | NA | NA | NA | NA | NA | NA | NA | NA | NA | NA | NA | NA | NA | NA | NA | NA | NA | NA | NA | NA | NA | NA | NA | NA | NA | NA | NA | NA | NA | NA | NA | NA |
| INT\_sq\_Ratio\_High/Low | MYSO | 0.0000 | 0.1653 | 0 | 0.0387 | NA | NA | NA | 0.0000 | NA | 0 | 0 | NA | NA | NA | NA | NA | NA | NA | NA | NA | NA | NA | NA | NA | NA | NA | NA | NA | NA | NA | NA | NA | NA | NA | NA | NA | NA | NA | NA | NA | NA | NA | NA | NA | NA | NA | NA |
| Ratio\_SPECIES | MYSO | 0.0000 | 0.0000 | 0 | 0.0000 | NA | NA | NA | NA | NA | NA | NA | 0.0000 | 0.0000 | 0.0000 | 0.0000 | 0.7267 | 0.7261 | 0.0050 | NA | 0.0000 | NA | NA | NA | NA | NA | NA | NA | NA | NA | NA | NA | NA | NA | NA | NA | NA | NA | NA | NA | NA | NA | NA | NA | NA | NA | NA | NA |
| log\_Ratio\_SPECIES | MYSO | 0.0000 | 0.0000 | 0 | 0.0000 | NA | NA | NA | NA | NA | NA | NA | 0.0000 | 0.0000 | 0.0000 | 0.0000 | 0.0000 | 0.0000 | 0.0000 | NA | 0.0000 | 0.0000 | 0.0000 | 0.0000 | 0.0000 | 0.0000 | 0.0000 | 0.0000 | NA | 0.0000 | NA | NA | NA | NA | NA | NA | NA | NA | NA | NA | NA | NA | NA | NA | NA | NA | NA | NA |
| INT\_log\_Ratio\_SPECIES | MYSO | 0.0000 | 0.0000 | 0 | 0.0000 | NA | NA | NA | NA | NA | NA | NA | 0.0069 | 0.1538 | 0.0067 | 0.0069 | 0.0000 | 0.0000 | 0.0042 | NA | 0.0223 | 0.0000 | 0.0000 | 0.0000 | 0.0000 | 0.0000 | 0.0000 | 0.0000 | NA | 0.0000 | 0.3210 | 0.8181 | 0.3162 | 0.3214 | 0.4360 | 0.4713 | 0.9778 | NA | 0.4077 | NA | NA | NA | NA | NA | NA | NA | NA | NA |
| sq\_Ratio\_SPECIES | MYSO | 0.0000 | 0.0000 | 0 | 0.0000 | NA | NA | NA | NA | NA | NA | NA | 0.0000 | 0.0000 | 0.0000 | 0.0000 | 0.0000 | 0.0000 | 0.3749 | NA | 0.0000 | NA | NA | NA | NA | NA | NA | NA | NA | NA | NA | NA | NA | NA | NA | NA | NA | NA | NA | 0.0002 | 0.0005 | 0.0002 | 0.0002 | 0.0000 | 0.0000 | 0.0226 | NA | 0.0005 |
| INT\_sq\_Ratio\_SPECIES | MYSO | 0.0000 | 0.0000 | 0 | 0.0000 | NA | NA | NA | NA | NA | NA | NA | 0.0000 | 0.0000 | 0.0000 | 0.0000 | 0.0000 | 0.0000 | 0.0000 | NA | 0.0000 | NA | NA | NA | NA | NA | NA | NA | NA | NA | 0.0775 | 0.1897 | 0.0784 | 0.0770 | 0.0000 | 0.0000 | 0.0000 | NA | 0.1504 | 0.0116 | 0.0103 | 0.0116 | 0.0115 | 0.5297 | 0.5161 | 0.2447 | NA | 0.0130 |
| Ratio\_Bats | PESU | 0.0000 | 0.0000 | 0 | 0.0000 | NA | NA | NA | NA | NA | NA | NA | NA | NA | NA | NA | NA | NA | NA | NA | NA | NA | NA | NA | NA | NA | NA | NA | NA | NA | NA | NA | NA | NA | NA | NA | NA | NA | NA | NA | NA | NA | NA | NA | NA | NA | NA | NA |
| log\_Ratio\_Bats | PESU | 0.0000 | 0.0000 | 0 | 0.0000 | 0 | NA | NA | NA | NA | NA | NA | NA | NA | NA | NA | NA | NA | NA | NA | NA | NA | NA | NA | NA | NA | NA | NA | NA | NA | NA | NA | NA | NA | NA | NA | NA | NA | NA | NA | NA | NA | NA | NA | NA | NA | NA | NA |
| INT\_log\_Ratio\_Bats | PESU | 0.0000 | 0.0071 | 0 | 0.1216 | 0 | 0.0000 | NA | NA | NA | NA | NA | NA | NA | NA | NA | NA | NA | NA | NA | NA | NA | NA | NA | NA | NA | NA | NA | NA | NA | NA | NA | NA | NA | NA | NA | NA | NA | NA | NA | NA | NA | NA | NA | NA | NA | NA | NA |
| sq\_Ratio\_Bats | PESU | 0.0000 | 0.0000 | 0 | 0.0000 | NA | NA | 0 | NA | NA | NA | NA | NA | NA | NA | NA | NA | NA | NA | NA | NA | NA | NA | NA | NA | NA | NA | NA | NA | NA | NA | NA | NA | NA | NA | NA | NA | NA | NA | NA | NA | NA | NA | NA | NA | NA | NA | NA |
| INT\_sq\_Ratio\_Bats | PESU | 0.0000 | 0.4986 | 0 | 0.0000 | NA | 0.0000 | 0 | NA | NA | NA | NA | NA | NA | NA | NA | NA | NA | NA | NA | NA | NA | NA | NA | NA | NA | NA | NA | NA | NA | NA | NA | NA | NA | NA | NA | NA | NA | NA | NA | NA | NA | NA | NA | NA | NA | NA | NA |
| Ratio\_High/Low | PESU | 0.0000 | 0.0000 | 0 | 0.0000 | NA | NA | NA | 0.0000 | NA | NA | NA | NA | NA | NA | NA | NA | NA | NA | NA | NA | NA | NA | NA | NA | NA | NA | NA | NA | NA | NA | NA | NA | NA | NA | NA | NA | NA | NA | NA | NA | NA | NA | NA | NA | NA | NA | NA |
| log\_Ratio\_High/Low | PESU | 0.0000 | 0.0000 | 0 | 0.0478 | NA | NA | NA | 0.0000 | 0e+00 | NA | NA | NA | NA | NA | NA | NA | NA | NA | NA | NA | NA | NA | NA | NA | NA | NA | NA | NA | NA | NA | NA | NA | NA | NA | NA | NA | NA | NA | NA | NA | NA | NA | NA | NA | NA | NA | NA |
| INT\_log\_Ratio\_High/Low | PESU | 0.0552 | 0.8560 | 0 | 0.0000 | NA | NA | NA | 0.0536 | 0e+00 | 0 | NA | NA | NA | NA | NA | NA | NA | NA | NA | NA | NA | NA | NA | NA | NA | NA | NA | NA | NA | NA | NA | NA | NA | NA | NA | NA | NA | NA | NA | NA | NA | NA | NA | NA | NA | NA | NA |
| sq\_Ratio\_High/Low | PESU | 0.0000 | 0.0000 | 0 | 0.0000 | NA | NA | NA | 0.0000 | NA | NA | 0 | NA | NA | NA | NA | NA | NA | NA | NA | NA | NA | NA | NA | NA | NA | NA | NA | NA | NA | NA | NA | NA | NA | NA | NA | NA | NA | NA | NA | NA | NA | NA | NA | NA | NA | NA | NA |
| INT\_sq\_Ratio\_High/Low | PESU | 0.0000 | 0.7295 | 0 | 0.0000 | NA | NA | NA | 0.0000 | NA | 0 | 0 | NA | NA | NA | NA | NA | NA | NA | NA | NA | NA | NA | NA | NA | NA | NA | NA | NA | NA | NA | NA | NA | NA | NA | NA | NA | NA | NA | NA | NA | NA | NA | NA | NA | NA | NA | NA |
| Ratio\_SPECIES | PESU | 0.0000 | 0.0000 | 0 | 0.0000 | NA | NA | NA | NA | NA | NA | NA | 0.0000 | 0.0017 | 0.0000 | 0.0000 | 0.0000 | 0.0003 | 0.0000 | 0.0000 | NA | NA | NA | NA | NA | NA | NA | NA | NA | NA | NA | NA | NA | NA | NA | NA | NA | NA | NA | NA | NA | NA | NA | NA | NA | NA | NA | NA |
| log\_Ratio\_SPECIES | PESU | 0.0000 | 0.0000 | 0 | 0.0000 | NA | NA | NA | NA | NA | NA | NA | 0.0181 | 0.0000 | 0.0180 | 0.0177 | 0.0205 | 0.7860 | 0.0164 | 0.0076 | NA | 0.4920 | 0.0000 | 0.4943 | 0.5014 | 0.4398 | 0.0000 | 0.5361 | 0.8541 | NA | NA | NA | NA | NA | NA | NA | NA | NA | NA | NA | NA | NA | NA | NA | NA | NA | NA | NA |
| INT\_log\_Ratio\_SPECIES | PESU | 0.0000 | 0.0000 | 0 | 0.0000 | NA | NA | NA | NA | NA | NA | NA | 0.2033 | 0.0000 | 0.2029 | 0.2029 | 0.2038 | 0.5374 | 0.2010 | 0.1648 | NA | 0.7092 | 0.0000 | 0.7117 | 0.7186 | 0.6575 | 0.0000 | 0.7535 | 0.9286 | NA | 0.5472 | 0.3087 | 0.5469 | 0.5480 | 0.5394 | 0.4937 | 0.5499 | 0.5257 | NA | NA | NA | NA | NA | NA | NA | NA | NA | NA |
| sq\_Ratio\_SPECIES | PESU | 0.0000 | 0.0000 | 0 | 0.0000 | NA | NA | NA | NA | NA | NA | NA | 0.0089 | 0.0000 | 0.0089 | 0.0088 | 0.0095 | 0.1310 | 0.0084 | 0.0066 | NA | NA | NA | NA | NA | NA | NA | NA | NA | NA | NA | NA | NA | NA | NA | NA | NA | NA | NA | 0.1312 | 0.0000 | 0.1311 | 0.1303 | 0.1368 | 0.6108 | 0.1273 | 0.1092 | NA |
| INT\_sq\_Ratio\_SPECIES | PESU | 0.0000 | 0.0000 | 0 | 0.0000 | NA | NA | NA | NA | NA | NA | NA | 0.0342 | 0.0000 | 0.0341 | 0.0337 | 0.0377 | 0.6885 | 0.0319 | 0.0227 | NA | NA | NA | NA | NA | NA | NA | NA | NA | NA | 0.9754 | 0.0000 | 0.9762 | 0.9792 | 0.9512 | 0.1926 | 0.9926 | 0.9275 | NA | 0.1170 | 0.0004 | 0.1171 | 0.1169 | 0.1175 | 0.1595 | 0.1167 | 0.1142 | NA |

## MAE Boxplots

Boxplots for each model. Contains values for each of the nine
Examined Species. Black horizontal line shows median. Outliers defined
as datapoints > 1.5 IQR below Q1 or above
Q3.

## RMSE Boxplots

Boxplots for each model. Contains values for each of the nine
Examined Species. Black horizontal line shows median. Outliers defined
as datapoints > 1.5 IQR below Q1 or above
Q3.

## R2 Boxplots

Boxplots for each model. Contains values for each of the nine
Examined Species. Black horizontal line shows median. Outliers defined
as datapoints > 1.5 IQR below Q1 or above
Q3.

## *Ratio\_Bats*

### *Count and Species Ratio Plot*

See text for details on plot
formation.

### *Fort Drum Military Installation Case Study Plots*

Plots using Species Ratio from Nocera 2019 with varying total audio
file counts. Error bars show +/- 2 MAE. See text for details.

#### *Total Files = 75*

#### *Total Files = 175*

#### *Total Files = 275*

#### *Total Files = 475*

### *MLE Plots*

Each simulated night’s model predicted MLE and the actual MLE from
the program.

Histogram of errors for each simulated night’s modeled MLE. Model MLE
- Program MLE = Error.

## *log\_Ratio\_Bats*

### *Count and Species Ratio Plot*

See text for details on plot
formation.

### *Fort Drum Military Installation Case Study Plots*

Plots using Species Ratio from Nocera 2019 with varying total audio
file counts. Error bars show +/- 2 MAE. See text for details.

#### *Total Files = 75*

#### *Total Files = 175*

#### *Total Files = 275*

#### *Total Files = 475*

### *MLE Plots*

Each simulated night’s model predicted MLE and the actual MLE from
the program.

Histogram of errors for each simulated night’s modeled MLE. Model MLE
- Program MLE = Error.

## *INT\_log\_Ratio\_Bats*

### *Count and Species Ratio Plot*

See text for details on plot
formation.

### *Fort Drum Military Installation Case Study Plots*

Plots using Species Ratio from Nocera 2019 with varying total audio
file counts. Error bars show +/- 2 MAE. See text for details.

#### *Total Files = 75*

#### *Total Files = 175*

#### *Total Files = 275*

#### *Total Files = 475*

### *MLE Plots*

Each simulated night’s model predicted MLE and the actual MLE from
the program.

Histogram of errors for each simulated night’s modeled MLE. Model MLE
- Program MLE = Error.

## *sq\_Ratio\_Bats*

### *Count and Species Ratio Plot*

See text for details on plot
formation.

### *Fort Drum Military Installation Case Study Plots*

Plots using Species Ratio from Nocera 2019 with varying total audio
file counts. Error bars show +/- 2 MAE. See text for details.

#### *Total Files = 75*

#### *Total Files = 175*

#### *Total Files = 275*

#### *Total Files = 475*

### *MLE Plots*

Each simulated night’s model predicted MLE and the actual MLE from
the program.

Histogram of errors for each simulated night’s modeled MLE. Model MLE
- Program MLE = Error.

## *INT\_sq\_Ratio\_Bats*

### *Count and Species Ratio Plot*

See text for details on plot
formation.

### *Fort Drum Military Installation Case Study Plots*

Plots using Species Ratio from Nocera 2019 with varying total audio
file counts. Error bars show +/- 2 MAE. See text for details.

#### *Total Files = 75*

#### *Total Files = 175*

#### *Total Files = 275*

#### *Total Files = 475*

### *MLE Plots*

Each simulated night’s model predicted MLE and the actual MLE from
the program.

Histogram of errors for each simulated night’s modeled MLE. Model MLE
- Program MLE = Error.

## *Ratio\_High/Low*

### *Count and Species Ratio Plot*

See text for details on plot
formation.

### *Fort Drum Military Installation Case Study Plots*

Plots using Species Ratio from Nocera 2019 with varying total audio
file counts. Error bars show +/- 2 MAE. See text for details.

#### *Total Files = 75*

#### *Total Files = 175*

#### *Total Files = 275*

#### *Total Files = 475*

### *MLE Plots*

Each simulated night’s model predicted MLE and the actual MLE from
the program.

Histogram of errors for each simulated night’s modeled MLE. Model MLE
- Program MLE = Error.

## *log\_Ratio\_High/Low*

### *Count and Species Ratio Plot*

See text for details on plot
formation.

### *Fort Drum Military Installation Case Study Plots*

Plots using Species Ratio from Nocera 2019 with varying total audio
file counts. Error bars show +/- 2 MAE. See text for details.

#### *Total Files = 75*

#### *Total Files = 175*

#### *Total Files = 275*

#### *Total Files = 475*

### *MLE Plots*

Each simulated night’s model predicted MLE and the actual MLE from
the program.

Histogram of errors for each simulated night’s modeled MLE. Model MLE
- Program MLE = Error.

## *INT\_log\_Ratio\_High/Low*

### *Count and Species Ratio Plot*

See text for details on plot
formation.

### *Fort Drum Military Installation Case Study Plots*

Plots using Species Ratio from Nocera 2019 with varying total audio
file counts. Error bars show +/- 2 MAE. See text for details.

#### *Total Files = 75*

#### *Total Files = 175*

#### *Total Files = 275*

#### *Total Files = 475*

### *MLE Plots*

Each simulated night’s model predicted MLE and the actual MLE from
the program.

Histogram of errors for each simulated night’s modeled MLE. Model MLE
- Program MLE = Error.

## *sq\_Ratio\_High/Low*

### *Count and Species Ratio Plot*

See text for details on plot
formation.

### *Fort Drum Military Installation Case Study Plots*

Plots using Species Ratio from Nocera 2019 with varying total audio
file counts. Error bars show +/- 2 MAE. See text for details.

#### *Total Files = 75*

#### *Total Files = 175*

#### *Total Files = 275*

#### *Total Files = 475*

### *MLE Plots*

Each simulated night’s model predicted MLE and the actual MLE from
the program.

Histogram of errors for each simulated night’s modeled MLE. Model MLE
- Program MLE = Error.

## *INT\_sq\_Ratio\_High/Low*

### *Count and Species Ratio Plot*

See text for details on plot
formation.

### *Fort Drum Military Installation Case Study Plots*

Plots using Species Ratio from Nocera 2019 with varying total audio
file counts. Error bars show +/- 2 MAE. See text for details.

#### *Total Files = 75*

#### *Total Files = 175*

#### *Total Files = 275*

#### *Total Files = 475*

### *MLE Plots*

Each simulated night’s model predicted MLE and the actual MLE from
the program.

Histogram of errors for each simulated night’s modeled MLE. Model MLE
- Program MLE = Error.

## *Ratio\_SPECIES*

### *Count and Species Ratio Plot*

See text for details on plot
formation.

### *Fort Drum Military Installation Case Study Plots*

Plots using Species Ratio from Nocera 2019 with varying total audio
file counts. Error bars show +/- 2 MAE. See text for details.

#### *Total Files = 75*

#### *Total Files = 175*

#### *Total Files = 275*

#### *Total Files = 475*

### *MLE Plots*

Each simulated night’s model predicted MLE and the actual MLE from
the program.

Histogram of errors for each simulated night’s modeled MLE. Model MLE
- Program MLE = Error.

## *log\_Ratio\_SPECIES*

### *Count and Species Ratio Plot*

See text for details on plot
formation.

### *Fort Drum Military Installation Case Study Plots*

Plots using Species Ratio from Nocera 2019 with varying total audio
file counts. Error bars show +/- 2 MAE. See text for details.

#### *Total Files = 75*

#### *Total Files = 175*

#### *Total Files = 275*

#### *Total Files = 475*

### *MLE Plots*

Each simulated night’s model predicted MLE and the actual MLE from
the program.

Histogram of errors for each simulated night’s modeled MLE. Model MLE
- Program MLE = Error.

## *INT\_log\_Ratio\_SPECIES*

### *Count and Species Ratio Plot*

See text for details on plot
formation.

### *Fort Drum Military Installation Case Study Plots*

Plots using Species Ratio from Nocera 2019 with varying total audio
file counts. Error bars show +/- 2 MAE. See text for details.

#### *Total Files = 75*

#### *Total Files = 175*

#### *Total Files = 275*

#### *Total Files = 475*

### *MLE Plots*

Each simulated night’s model predicted MLE and the actual MLE from
the program.

Histogram of errors for each simulated night’s modeled MLE. Model MLE
- Program MLE = Error.

## *sq\_Ratio\_SPECIES*

### *Count and Species Ratio Plot*

See text for details on plot
formation.

### *Fort Drum Military Installation Case Study Plots*

Plots using Species Ratio from Nocera 2019 with varying total audio
file counts. Error bars show +/- 2 MAE. See text for details.

#### *Total Files = 75*

#### *Total Files = 175*

#### *Total Files = 275*

#### *Total Files = 475*

### *MLE Plots*

Each simulated night’s model predicted MLE and the actual MLE from
the program.

Histogram of errors for each simulated night’s modeled MLE. Model MLE
- Program MLE = Error.

## *INT\_sq\_Ratio\_SPECIES*

### *Count and Species Ratio Plot*

See text for details on plot
formation.

### *Fort Drum Military Installation Case Study Plots*

Plots using Species Ratio from Nocera 2019 with varying total audio
file counts. Error bars show +/- 2 MAE. See text for details.

#### *Total Files = 75*

#### *Total Files = 175*

#### *Total Files = 275*

#### *Total Files = 475*

### *MLE Plots*

Each simulated night’s model predicted MLE and the actual MLE from
the program.

Histogram of errors for each simulated night’s modeled MLE. Model MLE
- Program MLE = Error.
